# Binary Switching of Calendar Cells in the Pituitary Defines the Phase of the Circannual Cycle in Mammals

**DOI:** 10.1016/j.cub.2015.09.014

**Published:** 2015-10-19

**Authors:** Shona H. Wood, Helen C. Christian, Katarzyna Miedzinska, Ben R.C. Saer, Mark Johnson, Bob Paton, Le Yu, Judith McNeilly, Julian R.E. Davis, Alan S. McNeilly, David W. Burt, Andrew S.I. Loudon

**Affiliations:** 1 Faculty of Life Sciences, University of Manchester, Manchester M13 9PT, UK; 2 Department of Physiology, Anatomy, and Genetics, Le Gros Clark Building, University of Oxford, South Parks Road, Oxford OX1 3QX, UK; 3 The Roslin Institute and Royal (Dick) School of Veterinary Studies, University of Edinburgh, Roslin, Midlothian EH25 9PRG, UK; 4 MRC Centre for Reproductive Health, Queen’s Medical Research Institute, Edinburgh EH16 4TJ, UK; 5 Faculty of Medical and Human Science, University of Manchester, Manchester, M13 9PT, UK

## Abstract

Persistent free-running circannual (approximately year-long) rhythms have evolved in animals to regulate hormone cycles, drive metabolic rhythms (including hibernation), and time annual reproduction. Recent studies have defined the photoperiodic input to this rhythm, wherein melatonin acts on thyrotroph cells of the pituitary pars tuberalis (PT), leading to seasonal changes in the control of thyroid hormone metabolism in the hypothalamus. However, seasonal rhythms persist in constant conditions in many species in the absence of a changing photoperiod signal, leading to the generation of circannual cycles. It is not known which cells, tissues, and pathways generate these remarkable long-term rhythmic processes. We show that individual PT thyrotrophs can be in one of two binary states reflecting either a long (EYA3^+^) or short (CHGA^+^) photoperiod, with the relative proportion in each state defining the phase of the circannual cycle. We also show that a morphogenic cycle driven by the PT leads to extensive re-modeling of the PT and hypothalamus over the circannual cycle. We propose that the PT may employ a recapitulated developmental pathway to drive changes in morphology of tissues and cells. Our data are consistent with the hypothesis that the circannual timer may reside within the PT thyrotroph and is encoded by a binary switch timing mechanism, which may regulate the generation of circannual neuroendocrine rhythms, leading to dynamic re-modeling of the hypothalamic interface. In summary, the PT-ventral hypothalamus now appears to be a prime structure involved in long-term rhythm generation.

## Highlights

• A circannual timer may reside in the pituitary pars tuberalis thyrotroph
• This is defined by a digital switching mechanism controlling EYA3 expression
• The circannual clockwork drives a morphogenic cycle in the PT and hypothalamus
• This involves recapitulation of a developmental program

## Graphical Abstract

**Figure:**
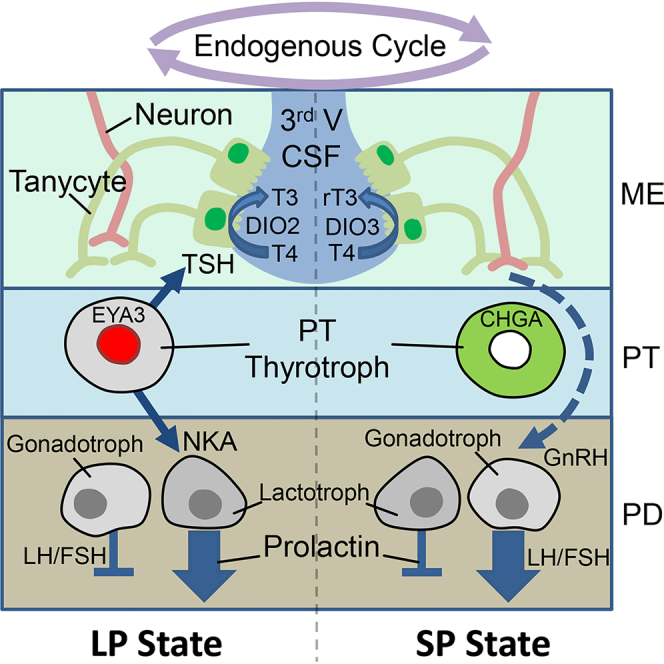

## Teaser

Circannual rhythms have evolved to regulate and time annual changes in physiology. Wood et al. report that the pars tuberalis generates the circannual rhythm in mammals through the digital switching of EYA3 expression. A recapitulated developmental pathway is used by the circannual clock to drive a morphogenic cycle in the PT and hypothalamus.

## Introduction

A complex repertoire of adaptive physiological cycles has evolved in vertebrates as a critical survival strategy for life in seasonal environments. These cycles are normally entrained by annual changes in day length (photoperiod), allowing animals to use a predictable changing signal to initiate complex physiological changes over the course of the year [1, 2]. This photoperiodic mechanism is known to entrain an underlying circannual clock, in which endogenous long-term free-running rhythms of physiology and behavior are tuned by an environmental signal. Circannual (approximately year-long) cycles are dominant features of the seasonal biology of mammals; they drive processes such as hibernation, metabolism, fattening, and reproductive activity [1–7] and persist when animals are kept in constant fixed photoperiods for many years [8–10].

The photoperiodic mechanism in vertebrates is known to involve seasonal regulation of thyroid hormones (THs), mediated in mammals via specialized cells (thyrotrophs) in the pituitary pars tuberalis (PT). It is not established whether this TH-input mechanism is also involved in the generation of long-term circannual cycles or whether separate molecular pathways and anatomical sites are involved. To address this, we examined the role of the circadian-controlled transcriptional co-activator EYA3 as the key upstream regulator of photoperiodic responses. Classic early studies [11] proposed that the circadian clock is co-opted for photoperiodic time measurement (external co-incidence hypothesis). It is posited that the core circadian clockwork drives a rhythmic circadian-regulated output gene(s), which then regulates a seasonal reproductive response. EYA3 is a strong candidate in mammals for this critical circadian-regulated gene [12]. A current model proposes that the rhythmic melatonin signal sets a local circadian oscillation in the PT via induction of the clock gene Cry1 [1, 13–15]. On short winter photoperiods (SPs), the peak phase of EYA3 is 12 hr after dark onset, coincident with nocturnal melatonin, which suppresses EYA3 expression [16]. On long photoperiods (LPs), the phase of expression is now coincident with light, and the gene is de-inhibited. This leads to co-activation of the TSHβ promoter in the PT thyrotoph, driving seasonal ependymal de-iodinase enzyme (DIO) signaling and TH metabolism [12]. Two de-iodinase enzyme genes (DIO2 and DIO3) reciprocally regulate to determine the local concentrations of the biologically active form of TH, triodothyronine (T3). TSHβ stimulation of DIO2 on LPs elevates T3 in specific neural sites, and this change has been shown experimentally to activate a summer-type physiology. The nature of the neuroendocrine cascade triggered by the summer brain T3 has been extensively reviewed recently (in birds and mammals [17, 18], bony fish [19], and seasonal hypothalamic relays [1, 20]), thus linking a proposed circadian mechanism to a seasonal output.

Importantly, we now show that the EYA3/TSHβ relay is not just a “slave” to photoperiod, but may be centrally involved in circannual timing. The PT operates a novel binary switching mechanism in which the relative proportion of individual EYA3-expressing cells reflects the circannual phase. We also reveal long-term morphogenic changes in neuroendocrine tissues and extensive remodeling of developmental programs over the circannual cycle. The PT is thus a prime candidate site in mammals for the generation of circannual rhythms.

## Results

### Expression of EYA3 and Chromogranin Protein in the PT

**Figure 1:**
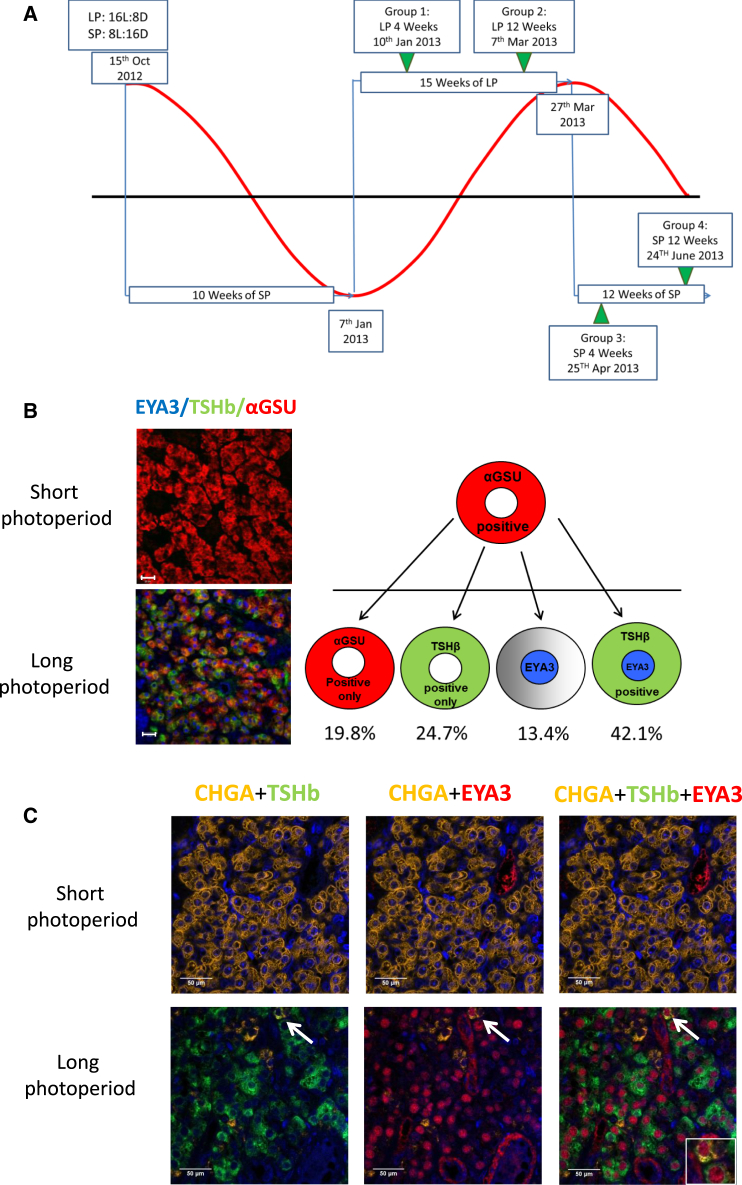
Co-localization of EYA3 and TSHβ to the PT Thyotroph on LPs and Identification of CHGA as a SP Marker (A) Diagram of the photoperiodic treatment for experiment 1. Short photoperiod (SP): 8 hr light, 16 hr dark; long photoperiod (LP), 16 hr light, 8 hr dark. Tissues were collected at 4 and 12 weeks in SPs and LPs, in the mid-light phase, zeitgeber time (ZT; time after lights on): ZT4 in SPs and ZT8 in LPs. Collection points are represented by green arrows. The red line illustrates the natural photoperiod, and the blue lines represent the photoperiod imposed in light-controlled rooms (Figure S1A). (B) Triple immunofluorescence showing expression of αGSU (red), TSHβ (green), and EYA3 (blue) in the PT on SPs and LPs (Figure S2). Scale bars, 20 μm. Quantification of EYA3 and TSHβ co-expression (Table S1 and Figure S3B) is shown as a schematic representing the variety of phenotypes that αGSU-expressing cells show in response to LPs. (C) Triple immunofluorescence showing expression of EYA3 (red), CHGA (yellow), and TSHβ (green) in the PT on SPs and LPs (blue, DAPI) (Figure S3C and Table S3). Scale bars, 50 μm. The white arrow shows a cell co-expressing EYA3, CHGA, and TSHβ; this is one of two cells found in over 17,000 that co-express all three proteins. See also Figures S1–S3.

We characterized photoperiod-regulated proteins in the PT, using tissues collected from castrate male sheep maintained in controlled light-dark cycles, following exposure to LP or SP conditions (Figures 1A and S1A). To define photoperiod-responsive cell types in the PT, we screened with antibodies raised against all of the major pars distalis (PD) endocrine cell types (Figure S2). In the PT, we only detected the common subunit αGSU, characteristic of thyrotroph cells, despite previous studies reporting a gonadotroph population in the PT [21–25] (Figure S2A). The other major cell type was S100^+^ cells (folliculostellate [FS] cells; Figure S3A). The PT is heavily vascularized, with arterioles and venules, but this gross structure and morphology did not change with photoperiod (Figure S3A). In the PT, on LPs, TSHβ protein was strongly co-expressed with αGSU-expressing cells (Figure 1B) but was non-detectable on SPs. In contrast, TSHβ protein did not change with photoperiod in the main PD thyrotroph population (Figure S2B).

We raised a novel ovine-specific EYA3 antibody (Supplemental Experimental Procedures), which revealed strong LP-specific co-localization of EYA3 to the αGSU-expressing “putative” PT thyrotroph (ZT4; Figure 1B). This supports a model for EYA3 co-activation of TSHβ expression, specifically within the putative PT thyrotroph [12, 16, 26]. To quantify EYA3 and TSHβ co-expression, we screened >4,000 αGSU^+^ cells from LP PT tissue (Table S1). We detected cells with αGSU alone (19.8% of cells), as well as TSHβ/αGSU^+^ (24.7%), EYA3/αGSU^+^ (13.4%), and TSHβ/EYA3/αGSU^+^ (42.1%) cells (Figures 1B and S3B). Thus, EYA3 is induced in a subpopulation of putative PT thyrotrophs after 4 weeks of LP exposure. We were unable to detect EYA3 protein in thyrotrophs from SP-derived PT tissue (Figure 1B); however, low-level mRNA expression is detectable on SPs [12, 16].

We next conducted RNA-sequencing (RNA-seq) studies of the PT, comparing tissues between LPs and SPs (Figure S1A, experiments 2 and 4; GEO: GSE65901). This identified a number of genes increased under SPs (Table S2). In order to identify a robust SP marker, we compared from different conditions RNA-seq datasets and identified five consistent SP-marker genes (PAQR6, NPSR1, C1orf110, chromogranin A [CHGA], and SOX14). Only one, CHGA, was highly expressed, showing a 3-fold upregulation with SPs (Table S2). We screened >2,000 PT cells with a CHGA-specific antibody and showed that CHGA protein expression in the PT is exclusive to the putative PT thyrotroph (Figures S3C and S3D and Table S3). Thus, CHGA provides a robust SP marker for the activity of the putative PT thyrotroph. Using CHGA, TSHβ, and EYA3, antibodies we observed that CHGA expression was greatly enhanced after 4-week exposure to SPs and suppressed by LPs (Figure 1C). Of note, αGSU expression was unchanged (Figures S2A and S3E).

Thus, in the PT, EYA3 and CHGA are expressed specifically in putative PT thyrotroph cells and map to LP and SP conditions, respectively.

### A Binary Cell-Based Timer over the Circannual Cycle

We next addressed whether these components exhibit long-term dynamic changes independently of photoperiod change. Many vertebrate species, including sheep [9], exhibit cycles of neuroendocrine activity when maintained on prolonged LPs [27, 28]. To achieve this, we kept cohorts of castrate male sheep (Figure S1A, experiment 3; born March/April) on natural photoperiods through to mid-October and then housed them in SP conditions for 12 weeks. Animals were then exposed to LPs for 29 weeks (LP29).

**Figure 2:**
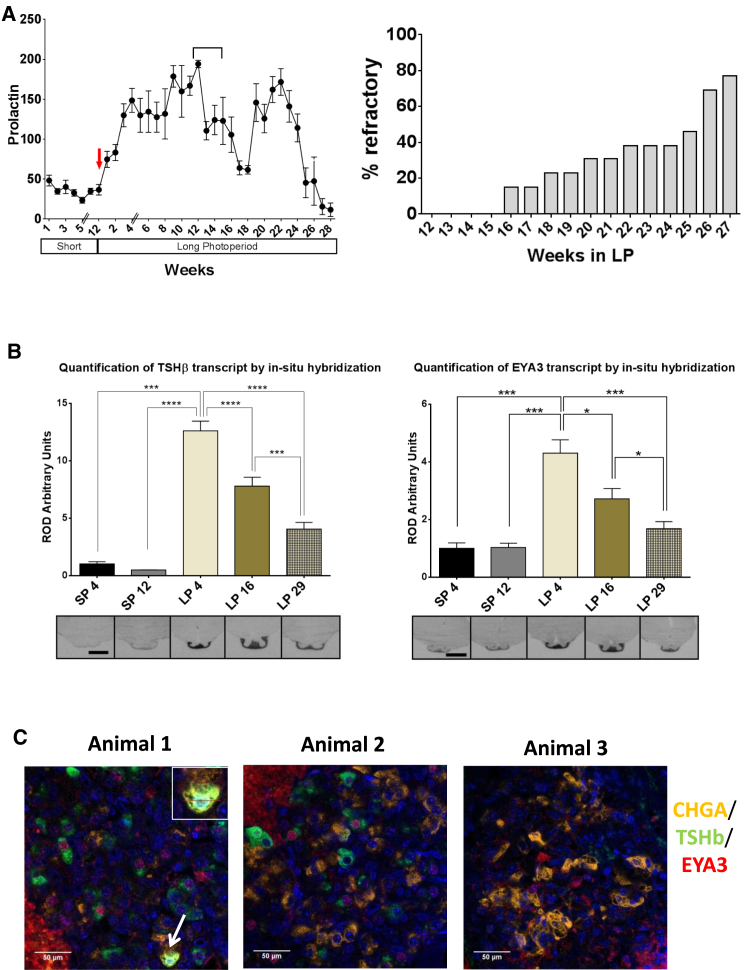
Binary Cell-Based Timing Mechanism over the Circannual Cycle (A) Prolactin concentrations in plasma for 32 animals in SPs for 12 weeks transitioning into LPs for 4 weeks (red arrow marks this). The first double line in the graph indicates the gap in weeks between sampling during SPs, and the second double line represents a change in the cohort of animals sampled (as the previous group culled at 4 weeks). The dip in prolactin concentration between 13 to 18 weeks may be related to wool shearing (indicated on the graph by a bar spanning the affected period). The percentage of animals that show three consecutive weeks of suppressed prolactin (LP refractory) is shown. Error bars represent the SEM. (B) In situ hybridization and quantification for TSHβ and EYA3 mRNA at SP4, SP12, LP4, LP16, and LP29. Representative images are shown (n = 3). Error bars represent the SD. (C) Triple immunohistochemistry showing protein expression of EYA3 (red), TSHβ (green), CHGA (yellow), and DAPI (blue) at LP29 in three individuals (Figures S3C and S4A and Table S4). Scale bars, 50 μm. See also Figures S2–S4.

In order to track a robust endocrine output, which maps to the phase of the circannual cycle [7–10, 29], we developed a novel competitive ELISA to assay changing prolactin concentrations (Figure 2A and the Supplemental Experimental Procedures). Reversion to the SP-like state (the LP-refractory response) was defined as a drop in circulating prolactin concentrations to SP levels in an individual animal for 3 weeks or more. By this criterion, 77% of animals were in a LP-refractory state by week 27 (Figure 2A).

Quantification of *EYA3* mRNA expression by in situ hybridization showed a strong 5-fold induction at LP4, a decline to approximately 50% of peak values by LP16, and a reversion of PT expression to SP-like levels by LP29 (Figure 2B). *TSHβ* mRNA shows a similar trend, with a significant decline at LP29 (Figure 2B). These changes reflected the underlying endocrine prolactin rhythm.

We assessed expression of CHGA, TSHβ, and EYA3 protein in putative PT thyrotrophs at LP29, using the prolactin history of each animal to assess its relative circannual phase, and found that all had declined to low SP prolactin concentrations for at least 3 weeks prior to sampling (Figure S2C). The extent to which EYA3 and TSHβ protein declined and CHGA increased to a SP-like pattern appeared to be related to the length of time that an animal exhibited sustained periods of low prolactin concentrations (Figures 2C and S4A and Table S4). Thus, the EYA3 expression pattern reflects the endogenous endocrine phase of individual animals and undergoes dynamic changes as they revert to a winter-like physiology in the LP-refractory state.

Critically, quantification of >17,000 individual cells at SP4, LP4, and LP29 only identified two cells (0.01%) that co-expressed EYA3, TSHβ, and CHGA (Figure 2C and Table S4). As we had already established that αGSU expression was unchanged (Figures S2A and S3E), we considered whether a specialized subset of αGSU-expressing cells might define either the LP or SP phenotype, respectively. However, cell counts revealed that in SPs, virtually all (91.6%) αGSU-expressing cells were CHGA^+^, whereas in LPs, 65.3% of αGSU-expressing cells were EYA3^+^. Thus, we propose that the most likely explanation is that within a population of thyrotrophs, individual cells rapidly switch phenotype coding for either the SP (CHGA^+^/EYA3^−^) or LP (EYA3^+^/CHGA^−^) state, with the relative proportion of these cell types changing over the circannual cycle.

### Photoperiod Drives a Morphogenic Transcriptional Program in the PT

We next set out to establish the full extent of transcriptional re-programming within the PT, in response to acute changes after transfer from SP to LP conditions (Figure S1A, experiment 2). There were significant changes in expression at days 1, 7, and 28 of LPs, with an increasing transcriptional response with time (40, 269, and 424 genes, respectively; Table S2). Enrichment analysis using DAVID revealed that secreted signal, cell motion/adhesion, and cell-cell signaling were all enriched at days 1, 7, and 28 (Table S2). Approximately half of all genes changing in each comparison were shared, with an additional unique set of genes specific to each time point (Figure S4B). Therefore, there are continuous dynamic changes during acute LP exposure over 4 weeks.

We next addressed whether these dynamic changes occur over a longer timescale (4 and 16 weeks of SPs or 4 and 22 weeks of LPs; Figure S1A, experiment 4; GEO: GSE65901) and revealed profound transcriptional changes in response to exposure to fixed LPs or SPs (Figure S4B and Table S2). Within each photoperiod, there were marked dynamic changes in expression with an independent set of genes contingent on the duration of exposure (Figure S4B). For example, there were 251 genes differentially expressed (DE) for SP4/SP16 comparisons, with strong enrichment for secreted glycoprotein and cell adhesion (Table S2).

**Figure 3:**
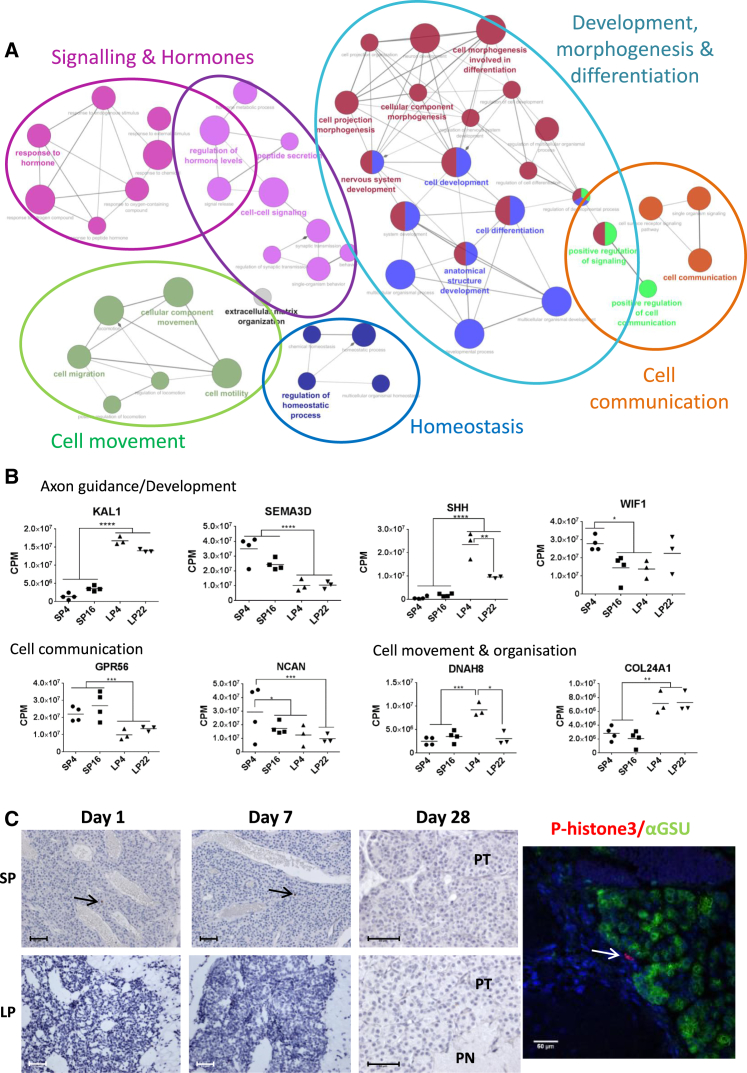
Transcriptional Response to Photoperiod and Histogenesis (A) A simplified network of related statistically significant GO terms using the Cytoscape add-on ClueGO [30, 31]. The network comparing SP4 versus LP4 is shown. The filled colored circles (nodes) represent each statistically significant parent GO term. The lines (edges) between the nodes show that there are overlapping genes within each term. The colored ovals group these parent GO terms into more generic functional descriptions (Figure S4B). (B) Graphs of average TMM normalized read counts per million (CPM) [32] from RNA-seq data from SP4, SP16, LP4, and LP22 (experiment 4). KAL1, SEMA3D, SHH, and WIF1 are within the following GO term categories: development, morphogenesis, and differentiation. GPR56 and NCAN are related to cell communication, and DNAH8 and COL2A1 are related to cell movement. False discovery rate (FDR)-corrected p values calculated by EdgeR are indicated as follows: ^∗^p < 0.05, ^∗∗^p < 0.005, ^∗∗∗^p < 0.0005, and ^∗∗∗∗^p < 0.0001 (Figures S4C and S5A). (C) Detection of dividing cells by phospho-histone H3 (p-histone3) and hematoxylin staining in the sheep pars tuberalis (PT) and pars nervosa (PN) under SP and LP at days 1, 7, and 28. Arrows show dividing cells. Scale bars, 50 μm. Double immunofluorescence staining of αGSU (green), phospho-histone H3 (red), and DAPI (blue) in ovine PT is shown (Figures S5B and S6). See also Figures S4–S6.

Although each time point was characterized by a unique subset of genes, we noted a marked similarity in function (Table S2). To assess this, we combined the DE genes from all comparisons (experiments 2 and 4) and assigned Gene Ontology (GO) terms to create a simplified network of related statistically significant GO terms [30, 31] (Figure 3A) and a heatmap for all comparisons (Table S5). These data emphasize enrichment for development, morphogenesis, cell communication, movement, signaling, and hormones. Finally, we plotted the expression profiles for a select panel of genes falling within our enriched terms, showing dynamic regulation over all the time points. Of note are genes involved in axon guidance (KAL1, SEMA3D [33]) and a morphogen involved in pituitary and nervous system development (sonic hedgehog, SHH) [34] (Figure 3B). We validated these RNA-seq changes using qPCR (Figure S5A) and also show SHH expression in the putative PT thyrotrophs by immunohistochemistry (Figure S4C).

Since our RNA-seq data revealed a consistent pattern of changes implicating cellular re-modeling and development, we tested whether photoperiodic change might initiate cellular division in the PT, using two markers, Ki67 (Figure S5B) and phospho-histone H3 (Figure 3C), to identify de-novo mitotic events (experiment 3). A similar number of a few dividing cells (<0.2%) were detected in each photoperiod condition, but none were co-expressed with αGSU (Figures 3C and S5B), and we were unable to identify significant evidence of mitotic events. We next selected a panel of four well-established cell-cycle-control genes (CDK6, CDC45, CDC25c, and CDT1), but none revealed altered expression with photoperiod (Figure S6). As a further check, we mined the RNA-seq data for DE genes that shared the GO term category “cell proliferation”; in all cases, these genes were associated with dominant parent terms such as development or morphogenesis (Table S6). Thus, our RNA-seq analyses and subsequent histological studies failed to identify direct evidence for de novo seasonal histogenesis in the PT thyrotroph, but we did detect significant enrichment for photoperiod-regulated genes involved in cellular re-modeling. These underwent dynamic changes, both in response to acute changes in photoperiod and after prolonged exposure to both SP and LP conditions. The extent to which the extensive transcriptional changes that we report are driven by a common EYA3-dependent mechanism or other upstream transcriptional activators remains to be determined.

### Cellular Re-modeling of the PT and Ventral Hypothalamus

**Figure 4:**
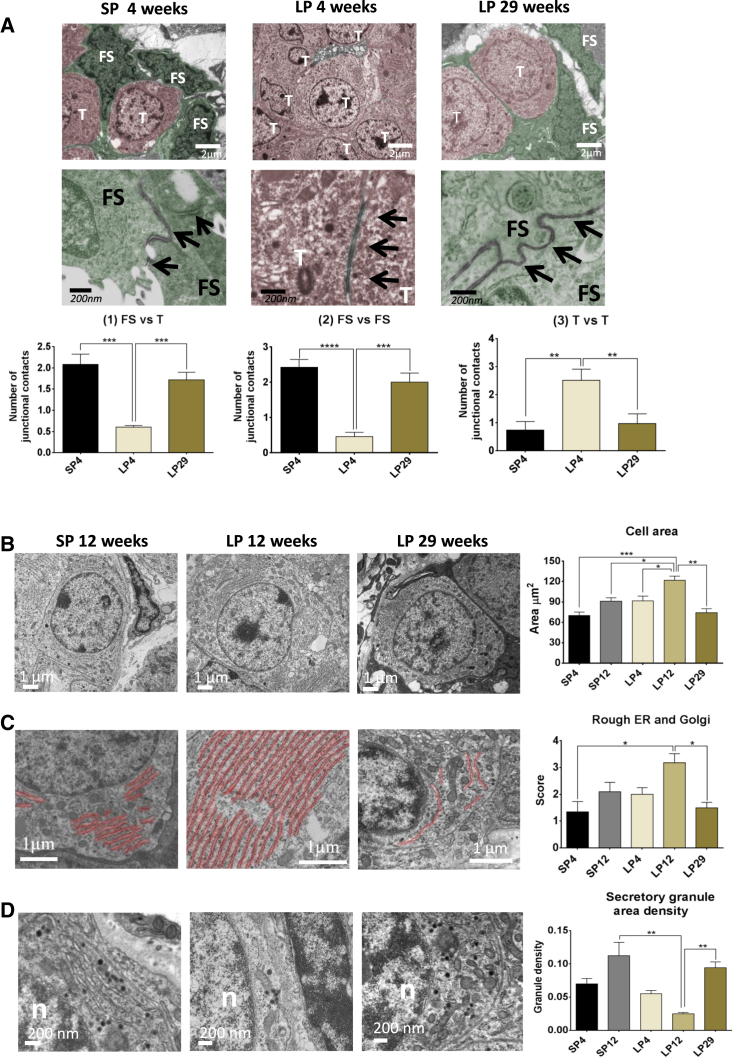
Cellular Remodeling in the PT (A) EM images for LP4/SP 4 and LP29. FS, folliculostellate cells (green); T, thyrotroph (pink). Arrows indicate intercellular junctions. n = 3. Representative images are shown. Quantification of cell contacts is shown as follows: (1) thyrotroph/FS contacts, (2) FS/FS contacts, and (3) thyrotroph/thyrotroph contacts. Cell contacts were identified on the basis of electron dense morphology between cells at the plasma membrane. The morphology represents a potential mix of junctions—zona adherens, desmosomes, and gap junctions. One-way ANOVA was performed with multiple testing corrections, with adjusted p values as follows: ^∗^p < 0.05, ^∗∗^p < 0.005, ^∗∗∗^p < 0.0005, and ^∗∗∗∗^p < 0.0001; n = 3. Error bars represent the SEM. (B) EM images of a PT thyrotroph at LP12, SP12, and LP29. Quantification of cell size is shown (μm^2^). One-way ANOVA was performed with multiple testing corrections, with adjusted p values as follows: ^∗^p < 0.05, ^∗∗^p < 0.005, ^∗∗∗^p < 0.0005, and ^∗∗∗∗^p < 0.0001; n = 3. Error bars represent the SEM. (C) Rough ER (RER) and Golgi false colored red and quantification of RER/Golgi. One-way ANOVA was performed with multiple testing corrections, with adjusted p values as follows: ^∗^p < 0.05, ^∗∗^p < 0.005, ^∗∗∗^p < 0.0005, and ^∗∗∗∗^p < 0.0001; n = 3. Error bars represent the SEM. (D) Dark spots show the granules present and quantification of secretory areal granule density. In all cases, SP4, SP12, LP4, LP12, and LP29 are presented with n = 3; representative images are shown. One-way ANOVA was performed with multiple testing corrections, with adjusted p values as follows: ^∗^p < 0.05, ^∗∗^p < 0.005, ^∗∗∗^p < 0.0005, and ^∗∗∗∗^p < 0.0001. Error bars represent the SEM. See also Figure S5.

The strong RNA-seq enrichment of developmental and morphogenic pathways suggested that a wider program of continuous re-modeling may occur. We therefore assessed ultrastructure of the PT using electron microscopy (EM) at SP4, SP12, LP4, LP12, and LP29 (Figure S1A, experiments 1 and 3). This revealed substantial photoperiod-driven changes in morphology of both thyrotrophs and FS cells in the PT. In SP conditions (SP4), putative PT thyrotrophs were dispersed with relatively few cellular contacts, surrounded by a network of FS cells, forming close cellular contacts (Figure 4A). Exposure to LPs (LP4) caused tissue re-organization, with thyrotrophs forming close cell contacts and dispersal of FS cells. By LP29, the thyrotroph/FS cellular morphology reverted to SP-like morphology, with dispersed thyrotrophs and close FS cell contacts (Figure 4A). To quantify these differences, we scored the number of junctional contacts between FS and thyrotroph cells at SP4, LP4, and LP29 (Figure 4A). In each case, the pattern of changes reverted at LP29 to a SP-like morphology. These data are in line with the strong enrichment within the PT transcriptome for cell-to-cell communication (Figure 3).

We next assessed PT thyrotroph morphology. There was a significant increase in size after LP exposure, with >2- and 3-fold increases in area and volume, respectively (Figure 4B). However, by LP29, the morphology of the thyrotroph had reverted to a SP-like state. These photoperiodic changes were accompanied by a marked increase in RER and Golgi on LPs (Figure 4C) and were inversely correlated with the density of secretory granule density, which was minimal at LP12 (Figure 4D). Staining for CHGA revealed strong co-localization to secretory granules on SPs (Figure S5C); however, immunogold labeling also revealed that CHGA was present in the cytoplasm and granules (data not shown). Again, these morphometric observations are supported by the RNA-seq results, which showed marked LP enrichment of genes involved in ER, Golgi, and vesicle formation (Table S5). Changes in RER, Golgi, secretory granules, and CHGA co-expression indicate an underlying complex and dynamic repertoire of secretory activities in these cells, which is significantly modified by photoperiod history. Importantly, these revert to a SP-like state in LP29 animals. Thus, the PT undergoes dynamic re-modeling over the seasonal cycle.

**Figure 5:**
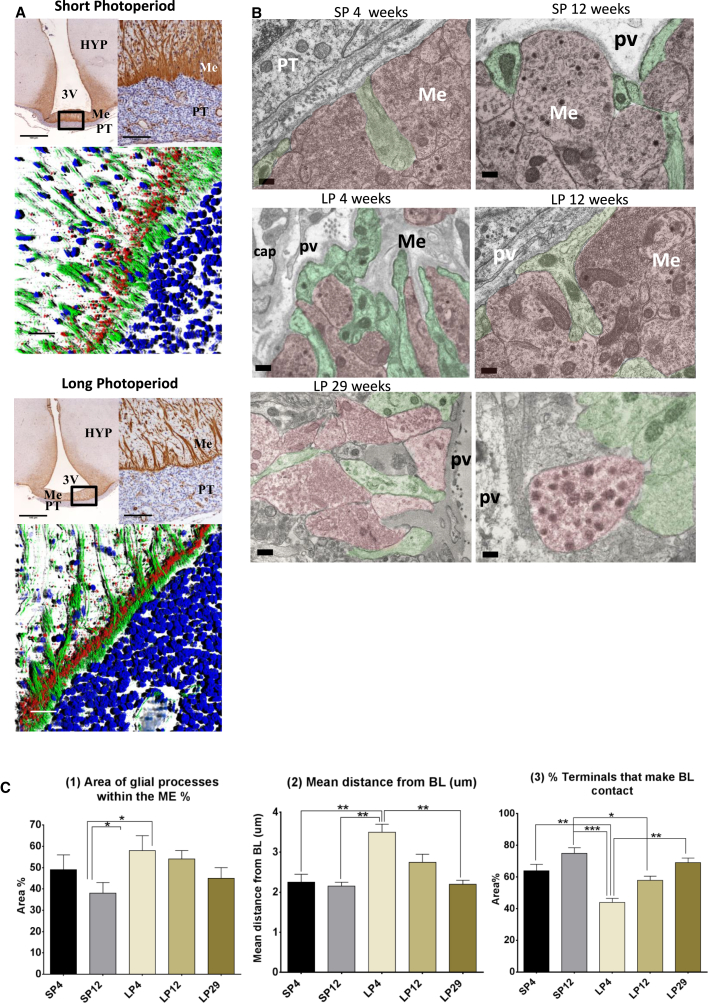
Remodeling of the Neural-Glial Interface of the Median Eminence (A) Vimentin immunostaining for tanycytes (brown) of coronal section of the sheep mediobasal hypothalamus (top). Scale bars, 100 μm and 20 μm, respectively. PT, pars tuberalis; Me, median eminence; 3V, third ventricle; HYP, hypothalamus. 3D render series of IHC images showing GnRH (red), vimentin (green), and DAPI (blue) in SPs and LPs are also shown (bottom). Scale bar, 50 μm. (B) EM images of ME nerve terminals (pink) and tanycytes (green) in SP4, SP12, LP4, LP12, and LP29. pv, perivascular space; cap, capillary. Scale bar, 200 nm. n = 3; representative images are shown. (C) Quantification of the (1) percentage area of glial process (tanycytic end foot) within the ME, (2) mean distance of the nerve terminal from the basal lamina (μm), and (3) percentage of nerve terminals in contact with basal lamina. One-way ANOVA was performed with multiple testing corrections, with adjusted p values as follows: ^∗^p < 0.05, ^∗∗^p < 0.005, ^∗∗∗^p < 0.0005, and ^∗∗∗∗^p < 0.0001; n = 3. Error bars represent the SEM. See also Figure S1.

**Figure 6:**
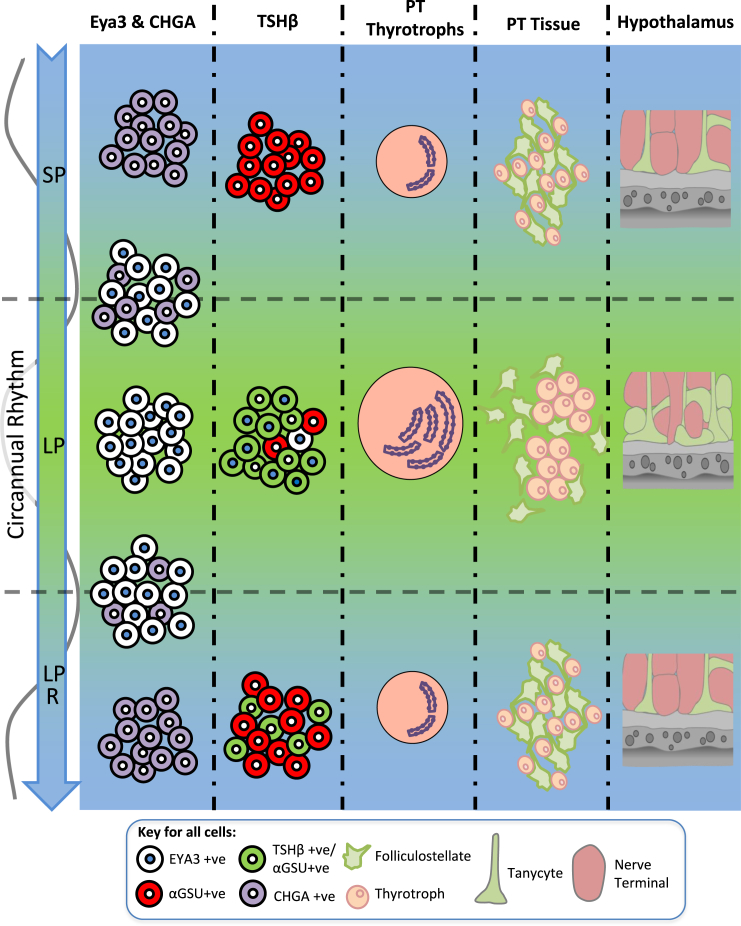
Summary of the Changes in the PT and Median Eminence throughout a Circannual Cycle The model proposes that an endogenous timer switches EYA3 expression in the PT thyrotroph cells, driving TSH and hypothalamic thyroid hormone metabolism independently of melatonin. Individual PT thyrotroph cells are either in a long (EYA3^+^) or short (CHGA^+^) state, and the relative proportion of these binary-state cells determines the phase of the circannual cycle. Re-modeling of the morphology of the PT sees changes in cell size and RER. Within the PT, networks of either thyrotrophs (LP) or FS cells (SP) form. Re-modeling of the hypothalamic interface in the ME leads to encasement of neuronal synapses by tanycyte end feet in the non-breeding season (LP), suggesting a physical mechanism for control of GnRH secretion. Collectively, this suggests that the PT thyrotroph operates as a calendar cell, generating long-term neuroendocrine rhythms in both the hypothalamus and pituitary gland.

The current model for photoperiodic regulation proposes that TSH of PT origin acts on adjacent ependymal cells in the hypothalamus to regulate the activity of the GnRH neurone [1, 35]. Seasonal changes in the neuro-glial interactions in the median eminence (ME) have been reported in the Japanese quail [36], but to date, there are no reports of similar changes in mammals. Vimentin staining revealed an extensive tanycyte network at the ME interface with the PT, which terminates in the basal lamina (Figure 5A). Staining for GnRH revealed a pattern of terminal fields within the tanycyte network. On LPs, there was a marked tanycyte barrier between GnRH terminals and the basal lamina (BL), with strong evident GnRH staining. In contrast, on SPs, GnRH staining extended through to the BL, and the tanycyte barrier was more dispersed. Further studies at the EM level revealed that there was a significant increase in contacts of the nerve terminals with the BL on SPs, whereas on LPs, the long tanycytic processes extended throughout the BL of the ME (Figures 5B and 5C). As a consequence, there was a highly significant reduction in the proportion of nerve terminals that made BL contact on LPs and a reciprocal increase in mean distance from the BL (Figure 5C). By LP29, these phenotypes had fully reverted to a SP state (Figure 5C). Thus, in addition to the substantial changes reported within the PT, we also reveal dynamic extensive re-modeling events at the neural-glial interface over the circannual cycle (Figure 6).

## Discussion

We employed the paradigm in a photoperiodic species in which the innate nature of the circannual timing mechanism is revealed by exposure to constant long day length over prolonged periods. Using this model, we addressed what role the photoperiodic relay mechanism might play in the initiation of a circannual rhythm. Two broad possible outcomes can be considered. First, the photoperiodic relay might faithfully record the environmental light cycle but does not contribute to circannual rhythm generation. Second, the input relay may itself also be part of the circannual timing mechanism, in which case it would be predicted to change spontaneously over the circannual cycle and map closely to the underlying physiology.

We now show that EYA3 protein is strongly induced specifically in PT thyrotrophs by LPs. Using in situ hybridization, we also show that prolonged exposure (6 months) to LPs leads to a marked suppression of both EYA3 and TSHβ mRNA in the PT. Immunohistochemical studies revealed a dramatic reduction in the number of cells expressing both proteins in the PT, the extent of which relates to the history of prolactin secretion in individual photo-refractory animals. Thus, using prolactin as an endocrine marker for the underlying circannual phase [7–10], our data support a model in which the photoperiodic-input mechanism within the PT is involved in the generation of the circannual cycle. The PT is also directly involved in seasonal prolactin regulation via an intra-pituitary mechanism involving the PT production of a paracrine signal independently of neural input [8, 16, 37], the nature of which is known to change spontaneously in photo-refractory animals [38]. Remarkably, hypothalamo-pituitary surgical disconnection (HPD) of the pituitary from the hypothalamus does not disrupt seasonal or circannual control of prolactin rhythms, strongly implicating a non-neural autonomous local mechanism in the regulation of this hormone [8, 9].

Recent studies have revealed dynamic circannual changes in DIO regulation in the ventral hypothalamic tanycyte cells of Soay sheep and Siberian and European hamsters [6, 39, 40] and PT TSHβ mRNA in European hamsters and Soay sheep [6, 39]. As EYA3 is known to regulate TSHβ, this further implicates the PT thyrotroph as being centrally involved in circannual rhythm generation. The changes in EYA3 expression that we report are unlikely to be driven by alterations in the duration of the nocturnal melatonin signal, as this hormone continues to report the prevailing photoperiod, irrespective of circannual phase [10]. Nor is it likely that altered phasing of the core circadian clockwork is involved, as core clock genes (*Per1* and *2*, *Cry1*, *Reverbα*, and *Bmal1*) in the PT do not change phase in photo-refractory sheep [10, 41].

Using CHGA as a short-day marker within the putative PT thyrotrophs, we showed that both the mRNA and protein are strongly induced in response to SPs. CHGA is essential for the formation of dense-core secretory granules and for regulating the secretion of pro-hormones in the gonadotrophs and somatotrophs of the PD [42, 43], and it reflects the physiological state of the SP thyrotroph. Remarkably, we were only able to detect two cells (0.01%) co-expressing TSHβ, EYA3, and CHGA. This suggests that the PT may record photoperiod history in the form of a binary code within individual thyrotroph cells, which rapidly flips from either a LP or SP state, but cannot be in both. Accordingly, the relative proportion of cells in each state may determine the overall activity of the PT, and hence the phase of the circannual cycle, as the PT reverts to a winter-like state after prolonged LP exposure, the proportion of CHGA^+^ cells increases as EYA3 and TSHβ decline. Whether the two processes are causally linked (i.e., decline in EYA3 directly initiates expression of CHGA) is not yet established. We are unaware of similar models driving seasonal biology in other animals; however, mathematical modeling has predicted a switching phenotype in response to photoperiod [44]. Furthermore, studies of vernalization mechanisms in plants propose a similar cell-based binary switching mechanism, which provides a memory of winter cold exposure and involves epigenetic control of a flowering repressor [45].

It is attractive to speculate that the dynamic continuous transcriptomic changes observed are driven by the changing ratios of cells expressing CHGA (SP) and EYA3 (LP). A conceptually similar mechanism exists in prolactin gene regulation within the rat anterior pituitary gland, in which the overall tissue response is generated by the activity of individual stochastically switching units [46].

A recent hypothesis has proposed that the circannual cycle may be generated by an underlying seasonal histogenesis in the PT, involving the reactivation of a latent stem cell population and an annual regenerative cycle [47–49]. We were unable to find evidence for significant de novo cell division. However, earlier studies have reported low level changes in cell division in the PT in response to photoperiod change; one reported an increase in division in LPs [49], but the other reported a decrease [48]. The latter study reported that these dividing cells were CD45^+^, suggesting that infiltrating immune cells may be the source of these dividing cells [48]. In addition, a recent study identified a very small number of dividing cells in the mouse PT [50]. In our study, we were unable to assess the extent to which histogenesis might be involved in circannual rhythm generation, but the very small numbers of dividing cells detected were certainly not thyrotrophs. In contrast, our morphological studies revealed extensive re-modeling within the PT and the thyrotrophs over the circannual cycle, with the SP state being characterized by elevated CHGA, secretory granule formation, and storage of pro-hormones [51], and the LP state by a secretory phenotype (i.e., increases in RER). Changes in the morphology and exocytotic activity in the PT-specific thyrotrophs have been reported in seasonal Djungarian hamsters and hibernating hedgehogs [52–54]. Thus, the PT may employ a recapitulated developmental pathway to drive changes in morphology of tissues and cells, which does not necessarily extend to activation of a latent stem cell population. Similar mechanisms have been documented in anterior pituitary cells, with hyperplasic changes in response to hormone stimulus, independently of cell division [55]. In this regard, other members of the EYA family are known to be key developmental regulators of the retina and other tissues [56–58], with EYA3 being co-opted for photoperiodic time measurement in the PT.

Our data suggest that the PT may establish morphogenic gradients, which extend to the ME, leading to retrograde signaling, and seasonal re-modeling of neuronal terminals and the associated tanycyte end feet. We identified enrichment for genes involved in establishing morphogenic gradients for GnRH neuron guidance in the developing brain in the PT (KAL1 and SEMA3D) [33]. In this model, the PT may both regulate DIO signaling in the hypothalamus and also drive changes in neuroendocrine morphology. In laboratory rodents, the plexin/semphorin (SEMA) signaling system is tightly controlled by gonadal steroids, leading to extensive re-modeling of tanycyte/GnRH neurones over the oestrous cycle [59, 60]. Alterations in the access of GnRH nerve terminals to the basal lamina of the ME have been reported in hamsters maintained in constant darkness [61]. Seasonal changes in GnRH accessibility to the have been shown in Japanese quail [36] and are of a similar magnitude to those we report here, but, in contrast to our data, this occurred on opposing photoperiods (i.e., tanycyte enclosure of neuronal synapses occurred on SPs in quail, rather than on LPs in sheep). Since the TSH/DIO/T3 hormone pathway is activated by LPs in a broadly similar manner in both seasonal birds and mammals [4, 62] irrespective of phase of breeding season, it would appear that local thyroid hormone changes cannot directly account for these differences. Intriguingly, this suggests that re-modeling of the neuroendocrine synapse may be linked to the phase of the reproductive cycle rather than the prevailing photoperiod. Since our studies were undertaken in castrated animals, changes in circulating sex steroids are unlikely to be involved.

We propose that the PT thyrotroph acts as a seasonal calendar cell with the capacity to generate long-term rhythms in mammals, driving both hypothalamic and pituitary endocrine circuits (Figure 6). Our studies cannot exclude the possibility that other structures (i.e., ependymal tanycytes) might also be involved. However, we implicate the same molecular mechanisms involved in the photoperiodic readout in the generation of long-term circannual cycles. The discovery of a role for thyrotroph cells in seasonal photoperiodism in birds and salmonid fishes [19, 63] suggests that this cell type may have a hitherto-unexpected evolutionarily conserved general role both in seasonal endocrine control and circannual rhythm generation across the vertebrate class. In summary, the PT-ventral hypothalamus now appears to be a prime structure involved in long-term rhythm generation.

## Experimental Procedures

All studies involving animals were licensed by the appropriate UK regulatory authority (Animals, Scientific Procedures Act, 1986), under a project license held by A.L. and approved by the local ethics committee. Scottish blackface sheep were housed in artificial light/dark cycles—either an 8:16 hr light/dark cycle for SPs or a 16:8 hr light/dark cycle for LPs. All animals were killed by an overdose of barbiturate (Euthatal; Rhone Merieux) administered intravenously, and sampling time points varied; see the Supplemental Experimental Procedures and figures for details. Hypothalamic blocks with the PT and pituitary attached were collected for immunohistochemistry, EM, and transcriptomics. Bioinformatic analysis was performed using Bowtie, SAMtools, HTSeq count, EdgeR, GSEA, DAVID, and Cytoscape. Ovine prolactin (oPRL) was measured using a newly developed competitive ELISA using purified oPRL (NIDDK-oPRL-21, AFP10692C; from Dr. A. Parlow, National Hormone and Peptide Program, Harbor-UCLA) and a highly specific rabbit anti-ovine prolactin (ASM-R50; produced by ASM) used previously in the specific radioimmunoassay [64]. In situ hybridization was performed as previously described [12]. The *OaTSHβ* plasmid (NCBI Gene: XM_004002368.2) was kindly provided by David Hazlerigg. The *OaEya3* plasmid (NCBI Gene: NM_001161733.1) was cloned as previously described [12]. Full details are provided in the Supplemental Experimental Procedures.

## Author Contributions

S.W. designed the experiments, collected samples, performed bioinformatic analysis and qPCR, and prepared the manuscript. H.C. performed the EM and analysis. K.M. performed IHC and analysis. B.S. collected samples and performed the in situ hybridization. M.J. performed EM and analysis. B.P. performed RNA preparation and sequencing. L.Y. performed bioinformatic analysis. J.M. developed the novel prolactin assay and prepared antibodies. J.D. designed experiments and revised the manuscript. A.M. collected samples, designed experiments, developed the prolactin assay, and revised the manuscript. D.B. designed experiments and revised the manuscript. A.L. designed experiments, collected samples, and prepared the manuscript.

## Accession Numbers

The accession number for the RNA-seq data reported in this paper is GEO: GSE65901.

## Supplemental Information

Document S1. Supplemental Experimental Procedures, Figures S1–S6, and Tables S1, S3, S4, and S6

Table S2. Statistically Significant Differentially Expressed Genes from the RNA-Seq Experiments

Table S5. Fully Annotated Heatmap for All RNA-Seq Comparisons

Document S2. Article plus Supplemental Information
