# Supplementary material for: Binary Switching of Calendar Cells in the Pituitary Defines the Phase of the Circannual Cycle in Mammals
Source: Curr Biol. 2015 Oct 19;25(20):2651–62. doi: 10.1016/j.cub.2015.09.014 (PMC4612467; doi:10.1016/j.cub.2015.09.014)
Supplement: Document S1. Supplemental Experimental Procedures, Figures S1–S6, and Tables S1, S3, S4, and S6 [file mmc1.pdf]

Current Biology

Supplemental Information

Binary Switching of Calendar Cells in the Pituitary Defines the Phase of the Circannual Cycle in Mammals

Shona H. Wood, Helen C. Christian, Katarzyna Miedzinska, Ben R.C. Saer, Mark Johnson, Bob Paton, Le Yu, Judith McNeilly, Julian R.E. Davis, Alan S. McNeilly, David W. Burt, and Andrew S.I. Loudon

Figure S1: Photoperiodic treatments and the gross morphology

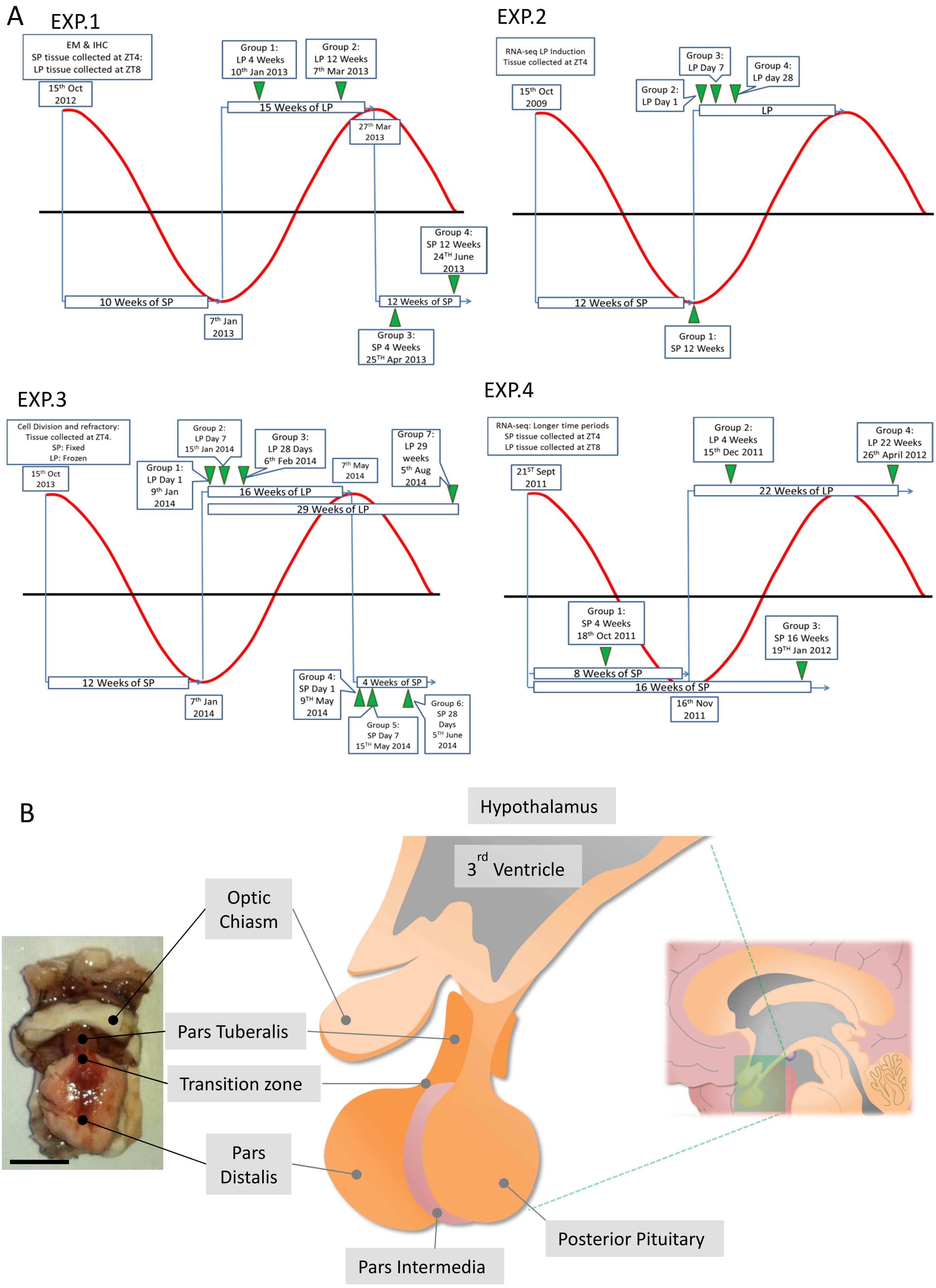

Figure S2: Characterisation of the cell types in the ovine pars tuberalis (PT) and pars distalis (PD) in a long and short photoperiod

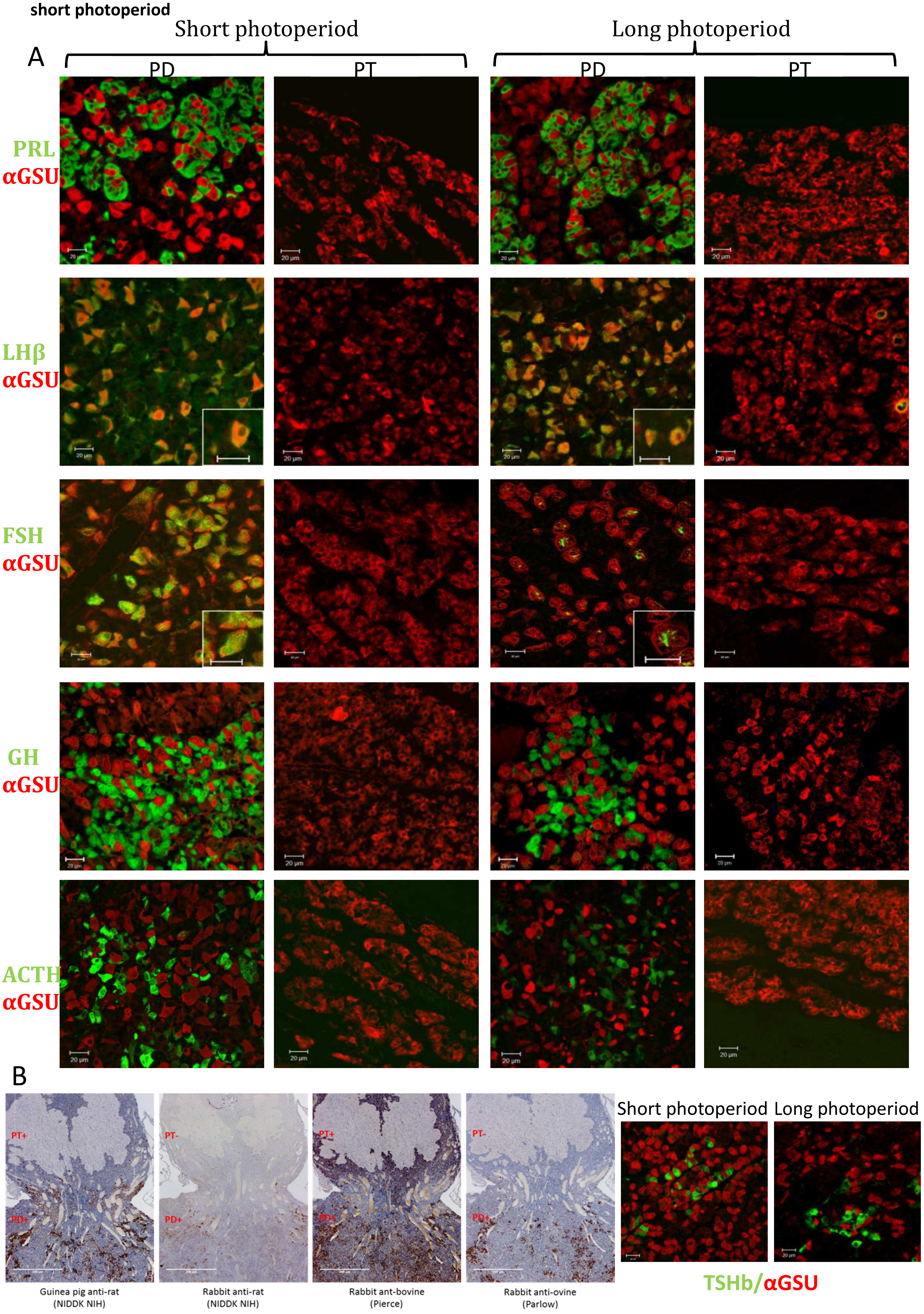

Figure S3: Major cell types of the PT and protein characterization

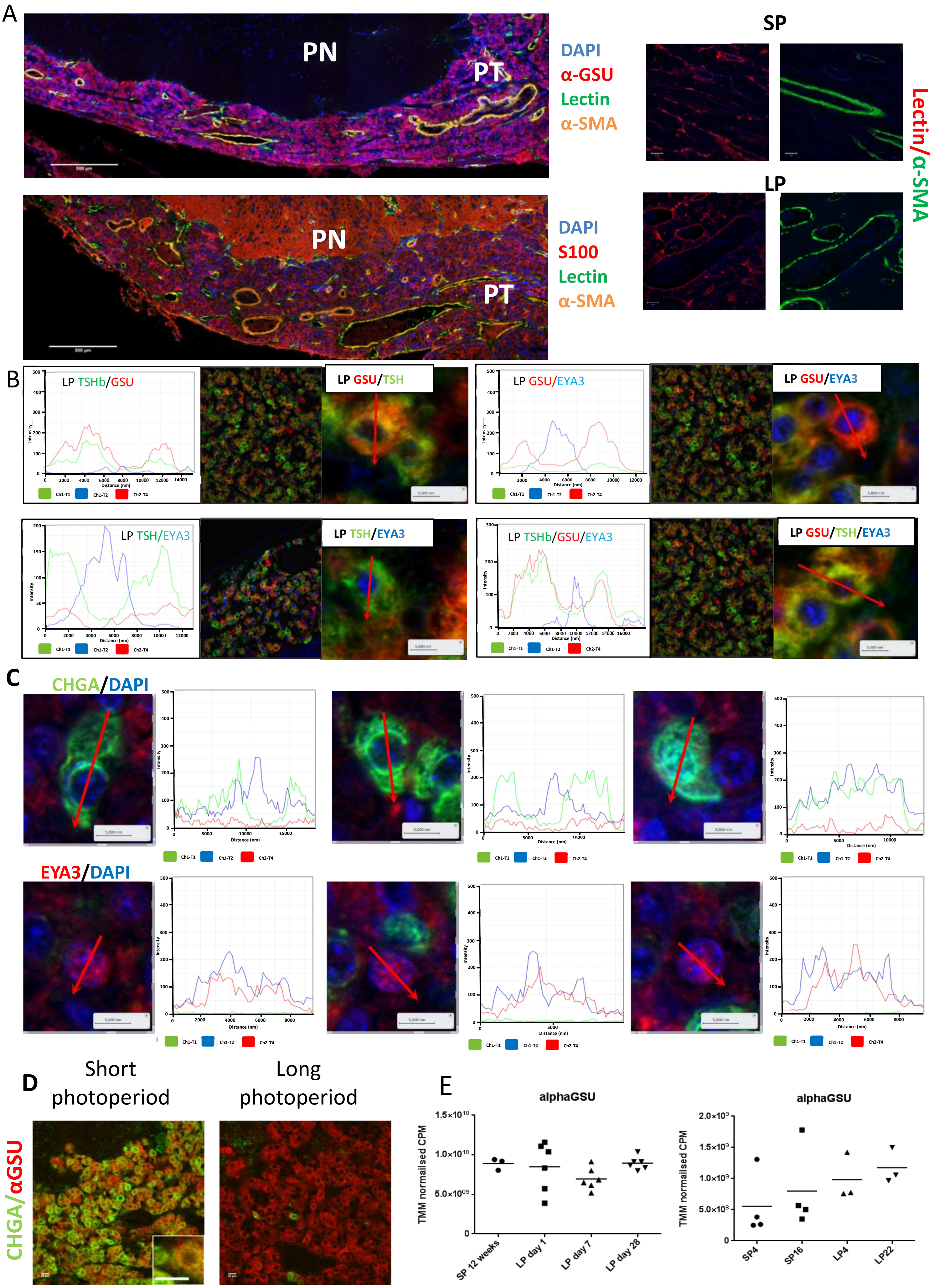

Figure S4: CHGA and EYA3 protein expression with individual prolactin concentrations, transcriptional characterisation and validation of SHH protein expression

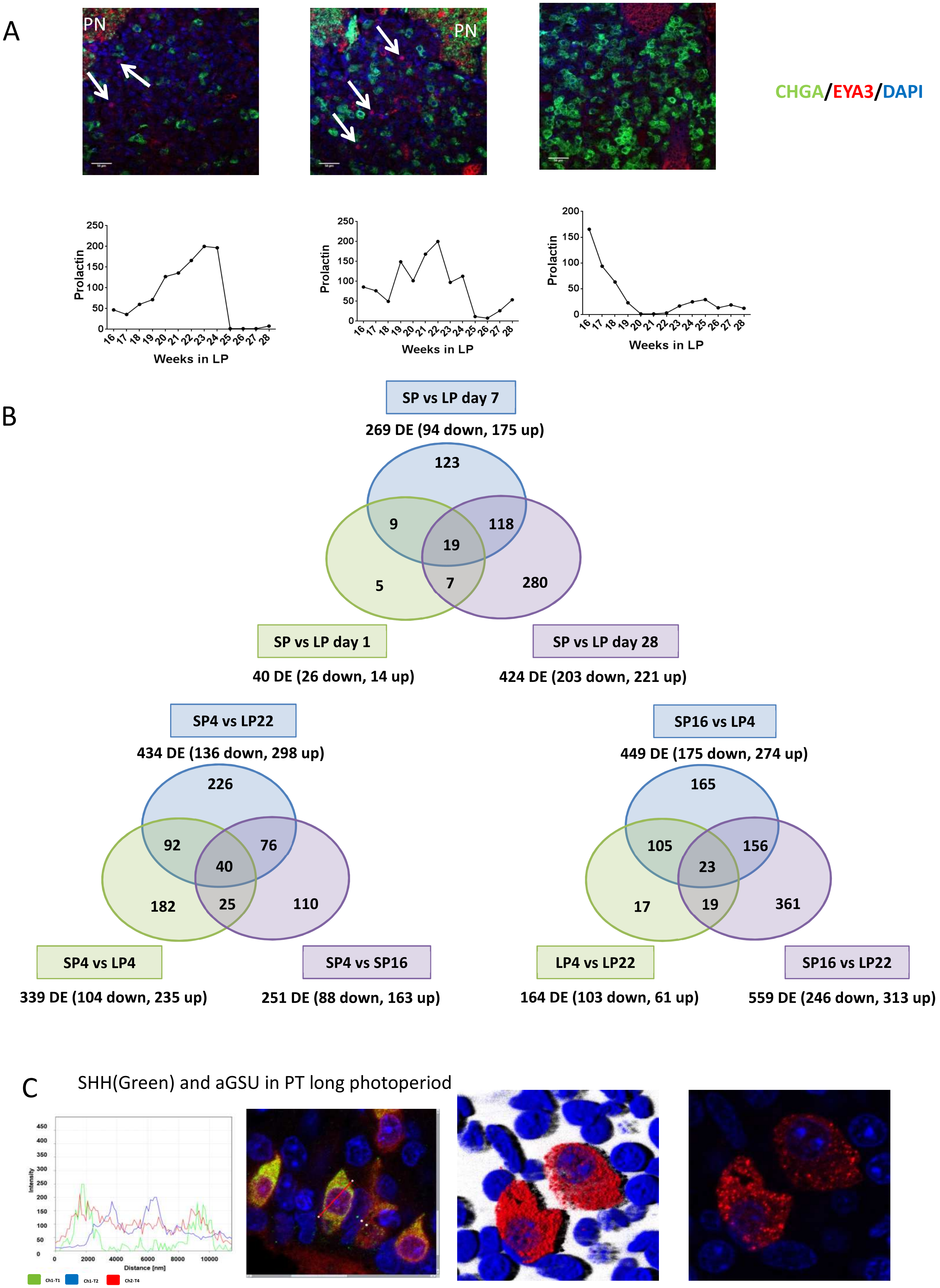

Figure S5: qPCR validation of RNA-seq, cell division marker Ki67 and CHGA co-localisation to granules

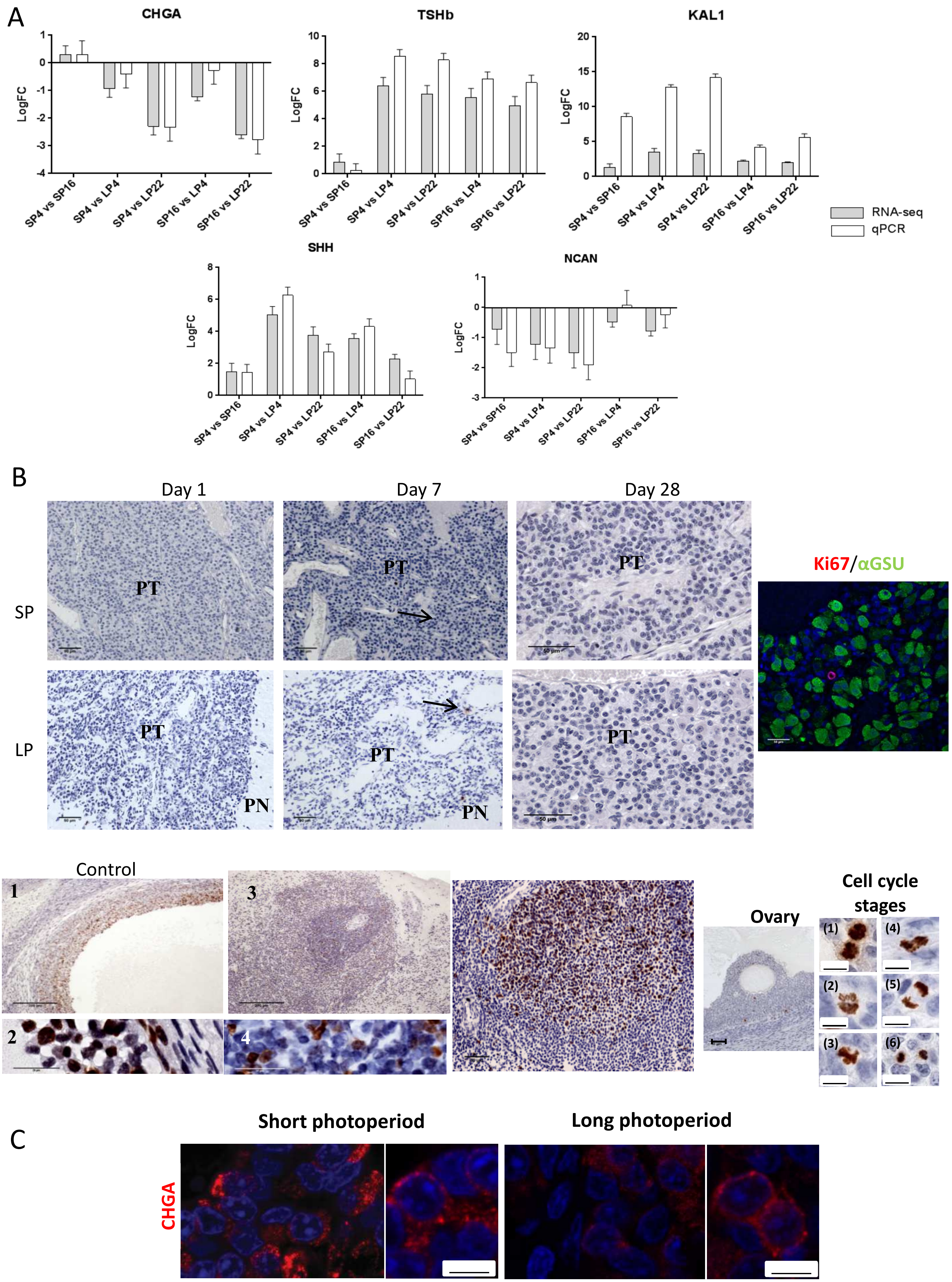

Figure S6: Cell cycle genes from RNA-seq

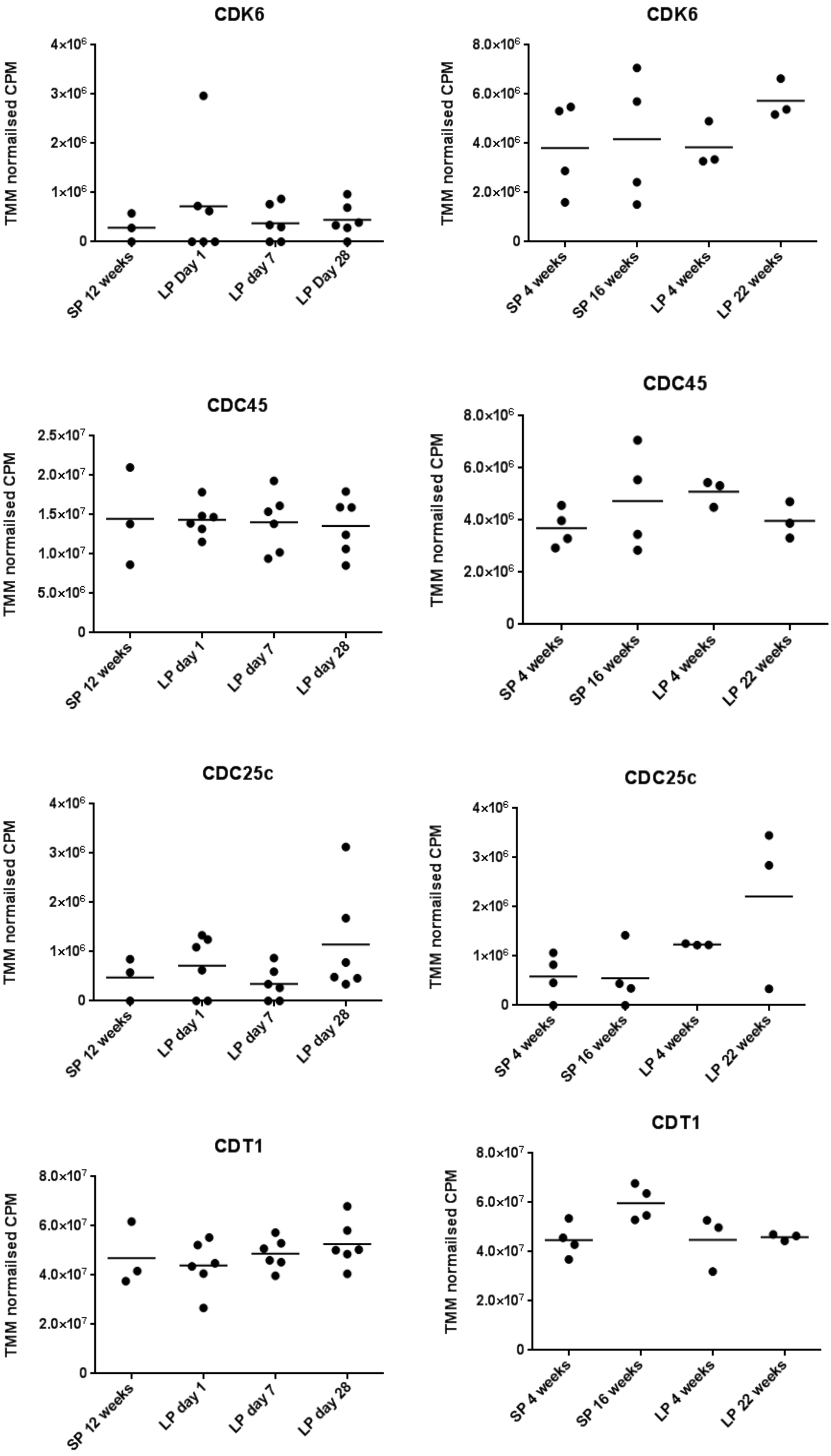

Figure legends

Figure S1: Photoperiodic treatments and the gross morphology relating to Figures 1 to 5

(A). Diagram of the photoperiodic treatments for Exp. 1 to 4. Collection points are represented by green arrows. The red line illustrates the natural photoperiod and the blue lines represent the photoperiod imposed in light controlled rooms.

(B). Diagram of the hypothalamus, optic chiasm, pars tuberalis, pars distalis, pars intermedia and posterior pituitary. Image of a hypothalamic block dissected from a sheep, indicating the transition zone between the PT and PD. Gonadotrophs (LHβ protein) have been previously reported in the PT [S1–S3], however expression was localized to this transition zone.

Figure S2: Characterisation of the cell types in the ovine pars tuberalis (PT) and pars distalis (PD) in a long and short photoperiod relating to Figure 1 & 2

(A). Double immunofluorescence showing an expression of αGSU (Red) with the main pituitary cell types (Green): lactotrophs (PRL), gonadotrophs (LH, FSH), somatotropes (GH), and corticotropes (ACTH). Scale bar=20µm.

(B). Four TSHb antibodies tested, Guinea pig anti-rat (NIDDK NIH) showing staining in the PT and PD, Rabbit anti-rat (NIDDK NIH) showing staining in the PD only, Rabbit anti-bovine (Pierce) showing staining in the PD and PT and Rabbit anti-ovine (Parlow) showing staining in the PD only. This is compatible with the recent discovery of tissue-specific glycosylation of PT-TSH, but not PD-TSH, as essential to maintaining their distinct functions [S4]; therefore it is likely that the post-translational modification of TSHβ [S5] affects the efficacy of the antibodies used. Double immunofluorescence showing an expression of αGSU (Red) with TSHb (Green) within the PD. Scale bar=20µm.

Figure S3: Major cell types of the PT and protein characterisation relating to Figures 1 & 2

(A). Triple immunohistochemistry of the PT and PN, sagittal section. αGSU staining for thyrotrophs (red) or S100 stain for folliculo-stellate cells (red), Lectin stain for blood vessels (green), alphaSMA stain for arterioles (orange). Venuoles are identified as lectin positive, alpha SMA negative Scale bar = 500um. PN – pars nervosa, PT – pars tuberalis. Smaller images for Lectin (red) and alpha-SMA (green) in SP and LP.

(B). Colour profiles and representative images used for the cell counting of TSHb (green), alphaGSU (red), and EYA3 (blue) in long photoperiod. Red arrows show the direction of scanning.

(C). Colour profiles and representative images of CHGA and EYA3, used for the cell counting, showing they are not co-localised. Red arrows show the direction of scanning.

(D). Double immunoflorescence showing expression of αGSU (Red) with CHGA (green) SP and LP in the PT or EYA3 (Red) with CHGA (green) SP and LP in the PT. (Table S3). Scale bar=20µm.

(E). TMM normalised counts from RNA-seq (Exp. 2 & 4) showing that αGSU expression, not significantly altered by photoperiod.

Figure S4: CHGA and EYA3 protein expression with individual prolactin concentrations, transcriptional characterisation and validation of SHH protein expression relating to Figures 2 & 3

(A). Protein expression of EYA3 (red) and CHGA (green) at LP29. Representative images showing that EYA3 and CHGA are not expressed in the same cell are included (5 animals, 6215 cells counted, Table S4). White arrows show EYA3 expressing cells. Scale bars = 50µm. Individual prolactin is also displayed for these animals, indicating a relationship between length of time that an animal exhibited sustained periods of low prolactin concentrations to the extent to which EYA3 and TSHβ protein declined and CHGA increased.

(B). Venn diagrams showing the number of significantly differentially expressed genes and the overlap with other comparisons. SP vs day 1, 7 and 28 are shown in the first venn diagram (Exp. 2). SP4 vs LP22, SP4 vs LP4 and SP4 vs SP16 are shown in the second venn diagram (Exp. 4) and finally SP16 vs LP4, LP4 vs LP22 and SP16 vs LP22 are shown in the last venn diagram (Exp. 4) (Tables S2)

(C). SHH (red) IHC showing co-localised to αGSU (green) expressing cells on long photoperiod. A 100% of SHH cells co-localize with αGSU.

Figure S5: qPCR validation of RNA-seq, cell division marker Ki67 and CHGA co-localisation to granules relating to Figures 3 & 4

(A). qPCR validation of RNA-seq results for TSHb, CHGA, KAL1, SHH, NCAN. RNA-seq LogFC shown in grey and qPCR LogFC shown in white. Error bars represent the standard error calculated as follows: (std error/mean)*log2e

(B). Detection of dividing cells by Ki67 and haematoxylin staining in the sheep pars tuberalis (PT) and (pars nervosa) under short and long photoperiod demonstrating only a minimal number (<0.2%) of single dispersed (arrows) dividing cells at day 1, 7 and 28 into the described photoperiod. Scale bar = 50um. For positive controls, sections spleen (3 – Bouins fixative, 5 – frozen tissue) were used and clearly show mitosis. Scale bar = 200um and 50um. Small images show positive staining of Ki67 in ovary (1 & 2) and spleen (3 & 4). Scale bar = 20um. Image 6 with a double immunofluorescent staining of aGSU (green) and Ki67 (red) in ovine PT shows that the dividing cells in the PT are not αGSU expressing cells. Sheep ovary for p-histone 3 clearly showing mitosis, Scale bar = 200µm. Small images (1-6) indicating p – histone H3 positive cells in ovary in different phase of the cell cycle, Scale bar = 10µm.

(C). IHC showing that CHGA (red) is localized to the granules in LP and SP. Scale bar = 5µm.

Figure S6: Cell cycle genes from RNA-seq relating to Figure 3

TMM normalised counts from the RNA-seq (Exp. 2 & 4) of four cell cycle related genes (CDK6, CDC45, CDC25c and CDT1), across groups SP, LP day 1, day 7 and day 28 (Exp. 2) and LP4, SP4, SP16 and LP22 (Exp. 4). No significant differences identified by EdgeR.

Table S1: Cell counts for TSHβ, EYA3 and αGSU in long photoperiod

N=3 animals, 2 sections from each animal, 2 fields from each section

Sheep no. LP3 - 75						
		Field 1	Field 2	Field 3	Field 4	Total n=4
	TSH	200	159	97	202	658
	GSU	173	193	139	192	697
	TSH + GSU	97	108	72	142	419
	Eya3	204	199	119	145	642
	TSH + Eya3	141	106	75	103	425
	GSU + Eya3	117	147	77	114	455
	GSU + TSH + Eya3	105	91	49	85	330
Sheep no. LP3- 76						
		Field 1	Field 2	Field 3	Field 4	Total n=4
	TSH	192	185	136	144	627
	GSU	236	221	255	186	898
	TSH + GSU	136	136	156	108	536
	Eya3	162	123	182	149	616
	TSH + Eya3	109	47	50	92	298
	GSU + Eya3	123	53	75	109	360
	GSU + TSH + Eya3	82	79	98	53	312
Sheep no. LP3 - 77						
		Field 1	Field 2	Field 3	Field 4	Total n=4
	TSH	175	222	161	271	829
	GSU	220	228	171	219	838
	TSH + GSU	143	159	121	241	667
	Eya3	207	233	163	153	756
	TSH + Eya3	136	147	115	93	491
	GSU + Eya3	151	153	119	113	536
	GSU + TSH + Eya3	119	123	95	92	429

Table S2: Statistically significant differentially expressed genes from the RNA-seq experiments

Exp. 2 comparisons: SP 12 weeks (ZT4) to LP day 1 (ZT4),) SP 12 weeks (ZT4) to LP day 7 (ZT4), SP 12 weeks (ZT4) to LP day 28 (ZT4).

Exp. 4 comparisons: SP 4 weeks (ZT4) to LP 4 weeks (ZT8), SP 4 weeks (ZT4) to SP 16 weeks (ZT4), SP 16 weeks (ZT4) to LP 4 weeks (ZT8), SP 4 weeks (ZT4) to LP 22 weeks (ZT8), LP 4 weeks (ZT8) to LP 22 weeks (ZT8), SP 16 weeks (ZT4) to LP 22 weeks (ZT8).

Supplied as an excel file

Table S3: Cell counts for CHGA and αGSU in long and short photoperiod

N=2 animals, 2 sections from each animal, 2 fields from each section

LP control 1	Area 1	Area 2	Area 3	Area 4	Totals
CHGA	8	21	4	4	37
aGSU	246	261	211	221	939
Co-loc.	8	21	4	4	37
LP control 2	Area 1	Area 2	Area 3	Area 4	Totals
CHGA	18	13	12	8	51
aGSU	233	219	131	133	716
co-loc.	18	13	12	8	51
SP control 1	Area 1	Area 2	Area 3	Area 4	Totals
CHGA	310	186	245	207	948
aGSU	324	185	286	238	1033
Co-loc.	310	186	245	207	948
SP control 2	Area 1	Area 2	Area 3	Area 4	Totals
CHGA	360	268	221	419	1268
aGSU	380	291	280	435	1386
Co-loc.	360	268	221	419	1268

Table S4: Cell counts for refractory animals: TSHβ, CHGA and EYA3

N = 3 animals, 4 sections from each animal, 2 fields of view per section

TSHb and EYA3 in refractory

	Area 1	Area 2	Area 3	Area 4	Area 5	Area 6	Area 7	Area 8	Total number
TSH 327	36	68	44	46	56	48	34	39	371
EYA327	0	0	0	0	0	0	0	0	0
Co-loc	0	0	0	0	0	0	0	0	0
	Area 1	Area 2	Area 3	Area 4	Area 5	Area 6	Area 7	Area 8	Total number
TSHb 519	108	91	81	27	89	101	114	134	745
EYA3 519	2	3	5	2	4	11	4	7	38
co-loc	2	3	5	2	4	11	4	7	1
	Area 1	Area 2	Area 3	Area 4	Area 5	Area 6	Area 7	Area 8	Total number
TSHb 520	57	92	112	98	142	121	84	80	786
EYA3 520	0	0	0	3	7	0	5	6	21
co-loc	0	0	0	3	5	0	3	4	15

CHGA & EYA3 in refractory and controls

	Area 1	Area 2	Area 3	Area 4	Area 5	Area 6	Area 7	Area 8	Total number
CHGA 327	210	179	213	176	199	197	205	180	1559
EYA3 327	0	0	0	0	0	0	0	0	0
CHGA 519	96	63	76	105	81	64	81	80	646
EYA3 519	0	0	0	7	5	11	5	6	34
CHGA 520	39	24	51	52	86	67	49	48	416
EYA3 520	1	0	1	0	4	8	4	3	21
LP control CHGA	27	42	23	26	17	53	23	17	228
LP control EYA3	77	43	64	63	81	81	81	90	580
SP control CHGA	405	375	323	288	316	349	338	337	2731
SP control EYA3	0	0	0	0	0	0	0	0	0

Table S5: Fully annotated heat map for all RNA-seq comparisons

Supplied as an excel file

Table S6: List of genes associated with the term cell proliferation in the RNA-seq data set

Gene symbol	entrez-gene name
BCHE	BCHE : butyrylcholinesterase
CCK	CCK : cholecystokinin
CGA	CGA : glycoprotein hormones, alpha polypeptide
CHRM4	CHRM4 : cholinergic receptor, muscarinic 4
DCT	DCT : dopachrome tautomerase
EGR1	EGR1 : early growth response 1
ESR1	ESR1 : estrogen receptor 1
EZH2	EZH2 : enhancer of zeste 2 polycomb repressive complex 2 subunit
FSHB	FSHB : follicle stimulating hormone, beta polypeptide
GLUL	GLUL : glutamate-ammonia ligase
GPLD1	GPLD1 : glycosylphosphatidylinositol specific phospholipase D1
EPCAM	EPCAM : epithelial cell adhesion molecule
MT3	MT3 : metallothionein 3
NGFR	NGFR : nerve growth factor receptor
NPY	NPY : neuropeptide Y
PGR	PGR : progesterone receptor
PRL	PRL : prolactin
SFRP4	SFRP4 : secreted frizzled-related protein 4
SHH	SHH : sonic hedgehog
TAC1	TAC1 : tachykinin, precursor 1
GPR56	GPR56 : G protein-coupled receptor 56
LRRC17	LRRC17 : leucine rich repeat containing 17
TCFL5	TCFL5 : transcription factor-like 5 (basic helix-loop-helix)
HPSE	HPSE : heparanase
TPX2	TPX2 : TPX2, microtubule-associated
HHLA2	HHLA2 : HERV-H LTR-associating 2
GMNC	GMNC : geminin coiled-coil domain containing
ACVR1C	ACVR1C : activin A receptor, type IC

Supplemental Experimental procedures

Animals

All animal experiments were undertaken in accordance with the Home Office Animals (Scientific Procedures) Act (1986), UK, under a Project License held by A.L. Animals were castrated as lambs on the farm as part of normal agricultural practice, and use of this castrate model allowed studies of seasonal changes in gene expression without the complication of altered background levels of sex steroids.

Scottish blackface male castrate sheep were housed in artificial light dark cycles, either 8:16 h light ⁄ dark cycle for short photoperiod (SP) or 16:8 h light ⁄ dark cycle for long photoperiod (LP). There were 4 different experiments conducted. Exp. 1 was conducted in 2012/2013; animals were housed from October on SP for 10 weeks and switched to LP for 15 weeks, collecting samples at 4 weeks LP and 12 weeks LP at ZT8. Animals were switched back on to SP for 12 weeks, collecting at SP 4 weeks and SP 12 weeks at ZT4. These samples were used for electron microscopy and IHC. Exp. 2 was undertaken in 2009, the animals were housed from October on SP for 12 weeks and then switched to LP. The day on which the photoperiodic switch was applied was designated "day 0". Animals were then culled at day 1, 7 and 28 of LP at ZT4, these samples were used in the LP induction RNA-seq experiment. Exp. 3 was conducted in 2013/2014; animals were housed from October on SP for 12 weeks and then switched to LP, the day on which the photoperiodic switch was applied was designated "day 0". Animals were then culled at day 1, 7 and 28 of LP at ZT4. The remaining animals were maintained on LP for 16 weeks, switching a cohort back to SP and collecting at day 1, 7 and 28, ZT4. These samples were used for the cell division experiments and IHC. Exp. 4 was conducted in 2011/2012; animals were housed from October on SP for 8 weeks, a cohort were maintained on SP up to 16 weeks and the other cohort were switched to LP for 22 weeks and used for the second RNA-seq experiment. Samples were collected in the mid-light phase at SP 4 weeks, SP 16 weeks, LP 4 weeks and LP22 weeks (ZT4 – SP, ZT8 – LP). A cohort of animals was maintained on LP for 29 weeks, prolactin was measured over the course of the experiment and the samples were used for EM and IHC (Exp. 3, ZT4). Finally, archived material was also used as previously described [S6, S7] for additional IHC studies. Figure S1 shows the design of all the experiments.

All animals were killed by an overdose of barbiturate (Euthatal; Rhone Merieux, Essex, UK) administered intravenously. Hypothalamic blocks with the pars tuberalis and pituitary attached were collected for immunohistochemistry, electron microscopy and transcriptomics (Figure S1B).

Prolactin assay and defining "refractory"anmals

Ovine prolactin (oPRL) was measured using a newly developed competitive ELISA using purified oPRL (ovine prolactin NIDDK-oPRL-21; AFP10692C; from Dr. A Parlow, NHPP, Harbor-UCLA Torrance CA, USA) and a highly specific rabbit anti-ovine prolactin (ASM-R50, produced by ASM) used previously in a specific radioimmunoassay [S8]. Three replicates of oPRL (100µg each) were biotinylated using NHS-LC-Biotin (Thermo Pierce; Fisher Scientific UK Ltd, Loughborough, UK). And the resulting biotinylated oPRL preparations were assessed in the ELISA. When test control samples were assayed, the ELISA gave results, which were comparable to the levels previously measured by the original RIA. The ELISA was established on standard 96 well plates (Nunc Maxisorb Immuno Plates; Nunc A/S, Roskilde, Denmark) previously coated with capture antibody (affinity purified donkey anti-rabbit IgG; Jackson ImmunoResearch Laboratories Inc, Newmarket, Suffolk CB8 7SY UK). The rabbit anti-oPRL antiserum (ASM-R50; 1:160,000 dilution) was added followed by addition of samples or standards and biotinylated oPRL, and plates incubated for at least 14h at 4°C. Plates were then washed 5 times before detection of biotinylated oPRL by addition of streptavidin HRP (GE Healthcare UK, Little Chalfont, UK) for a minimum of 30 minutes followed by TMB peroxidase substrate (KPL, Gaithesburg, MD 20878, USA). Colour was allowed to develop for up to 10 min, then stopped by addition of 6% phosphoric acid, and plates were read at 450nm. The Coefficient of Variation for the assay on control plasma samples was <10%.

We calculated the mean prolactin concentration in SP across multiple weeks and animals (n=32) and then calculated the median of the means and added 20% to define a SP-like prolactin concentration (SP prolactin = 41.03). We then defined an animal as "refractory" when their individual prolactin level remained at SP-like concentrations for 3 consecutive weeks. Once an animal met this criterion we noted the first week the animal had a SP-like prolactin value and defined the animal as refractory from that week.

EYA3 antibody

A polyclonal EYA3 antibody was raised in chicken (Cambridge research biochemical, Billingham, UK). The peptide sequence was based on homology with the sheep and human (Acetyl-PEQPVKKAKMQESGEQTLS-[C]-amide). The ovine EYA3 peptide was prepared at >80% purity and conjugated with KLH for immunization. Protein precipitation and isolation of IgY from eggs was performed and ELISA analysis at day 0, 49, 63, 77, 91 and 105 were carried out. Two elutions per antibody were supplied: Glycine and TEA. The glycine eluate was used for IHC.

Immunohistochemistry

Tissues were immersed in Bouin's fixative for 8 hours, transferred to 70% ethanol, then dehydrated and embedded in paraffin wax. Sections (5 µM) were cut, floated onto Superfrost Plus slides (J1800 AMNZ, Thermo scientific), dried at 50°C overnight, then dewaxed and rehydrated.

Frozen sections (8 µM) were collected on to Poly-lysine coated slides and stored in -80°C. Before immuno-detection, sections were fixed for 10 min at room temperature in 4% paraformaldehyde then rinsed in PBS. For localization of dividing cell markers standard single immunofluorescence was performed.

Double immunofluorescence was carried out for co-localization of PRL, LHβ, FSHβ, GH, TSHβ, ACTH, S100 and CHGA antigens with α-GSU. Slides were washed in PBS buffer (P4417, Sigma) between treatments. For LH and CHGA, antigen retrieval [S9] was carried out by pressure cooking for 5 min at full pressure in 0.01 M citrate buffer, pH 6.0. For all sections endogenous peroxidase activity was blocked by incubating sections in 3% (v/v) hydrogen peroxidase in methanol for 30 min. Sections were blocked using 20% normal goat serum, 5% BSA in PBS (NGS/PBS/5%BSA) for 30 min, then incubated in a humidified chamber overnight at 4°C in the first primary antibody diluted in blocking buffer as indicated. Antibody dilutions were as follows: rabbit anti – PRL 1:100K (R51, ASM-HRSU), mouse anti-LHβ 1:50K (kindly gifted by J. Roser, University of California), rabbit anti-FSHβ 1:25K (kindly gifted by S. Lynch, Birmingham), mouse anti-GH 1:500 (gift from Prof. M. Wallis, University of Sussex, Brighton), rabbit anti-bovine TSH 1:2K (kindly gifted by J. Pierce), mouse anti-ACTH 1:100 (NCL-ACTH, Novocastra), rabbit anti-S100α (ab11428, Abcam) 1:500, rabbit anti-CHGA 1:4K (Incstar), and αGSU 1:2K (ASM-HRSU, R20). Slides where then incubated with first secondary antibodies. PRL, FSHβ, TSHβ, S100α, FSHβ and α-GSU slides were incubated with goat anti-rabbit peroxidase (Vector Laboratories PI-1000) diluted 1:500 for 1h. LH, GH, ACTH slides were incubated with goat anti – mouse peroxidase (Abcam, ab6823) diluted 1:500 for 1 h, then followed by TSA (NEL741B00IKT, Perkin Elmer) diluted 1:50 in kit buffer for 10 min in the dark. PRL, FSHβ, TSHβ, S100α and a-GSU slides were performed in 0.01 M citrate buffer (2.5 min in boiling solution in microwave followed by a cool down at room temperature for at least 15 min [S10]. After that all sections were blocked in NGS/PBS/5%BSA for 30 min, and then incubated overnight at 4°C in the second primary antibody, αGSU, diluted 1:2.5K in blocking buffer or in the case of slides labelled with first primary antibody αGSU were incubated with CHGA antibody. All section were then incubated in the second secondary antibody, either goat anti-rabbit or mouse peroxidise IgG diluted 1:500 (Vector Laboratories PI-1000) for 1 hours, followed by TSA (NEL744B00IKT, Perkin Elmer) diluted 1:50 in kit buffer for 10 min in the dark. Control sections were incubated with NGS/PBS/5%BSA in place of the first, second primary or both first and second primary antibodies. For localization of αGSU or S100α with αSMA (mouse anti-αSMA at 1:1K (M0851, Dako)) and Lectin standard double immunofluorescence with an additional stage for third primary and secondary antibodies was performed. For S100 and αSMA detection antigen retrieval was carried out by boiling in 0.01 citrate buffer pH6 for 6.5min in a microwave. First primary antibody dilutions were as follows: rabbit anti – human S100 1:500, rabbit anti – ovine αGSU 1:2K. Slides where then incubated with first secondary antibodies goat anti – rabbit peroxidase, and then followed by TSA (NEL745001KT, Perkin Elmer). The second primary antibody, mouse anti – human αSMA (M0851, Dako) was diluted 1:2K follow by the second secondary antibody, goat anti – mouse peroxidase (ab6823, Abcam) and then TSA (NEL741001KT). The third primary antibody biotin labelledlabelled Lectin, (L3759, Sigma) diluted 1:40 in the serum was detected using Streptavidin Alexa 488 (S11223, Molecular Probes) at 1:200 dilution in PBS. Subsequently, all samples were incubated in DAPI (D9542, Sigma) diluted 1:500 in PBS for 10 min in the dark. Sections were coverslip mounted using PermaFluor^{TM} mounting medium (TA-030-FM, Thermo scientific). All samples were imaged under a Zeiss LSM 710/510 confocal microscope with a 40× oil-immersion objective. The cell counting was done manually using Adobe Photoshop CS6.

Conventional methods were used for the immunohistochemical detection of Ki67 and phosphor-histone H3, markers of dividing cells and PC1. lides were washed in TBS pH=7.4 buffer between treatments. The primary antibodies were diluted at 1:100 for Ki67 and 1:500 for phospho–histone H3 and were detected using biotinylated secondary goat

anti – rabbit in 1:500 dilution and streptavidin-peroxidase conjugate in 1:1000 dilution. Peroxidase activity was detected using a DAB solution. Sections were counterstained with Mayer's haematoxylin, then dehydrated and coverslip mounted using Pertex mounting medium.

In Situ Hybridization (ISH) and Quantification of Signal

The *OaTSHβ* plasmid (XM_004002368.2) was kindly provided by David Hazlerigg. The *OaEya3* plasmid (NM_001161733.1) was cloned as previously described [S6]. Frozen coronal ovine hypothalamic blocks for in-situ hybridization were cut into 16µm sections using a cryostat (CM3050s Leica Microsystems, Ltd., Milton Keynes, UK), and thaw mounted onto poly-l-lysine coated slides (VWR International, Lutterworth, UK). Radiolabelled cRNA riboprobes were prepared by plasmid linearization and transcribed using P^{33} α-UTP (Perkin-Elmer). Fixed sections were hybridized overnight at 60°C with 15 x 10^5 cpm of probe per slide. Hybridization signals were visualised on autoradiographic film (Kodak Biomax MR Films, Kodak, USA) after one week exposure at -80°C. Signal intensity was quantified by densitometry analysis of autoradiographs using the image-Pro Plus 6.0 software (Media Cybernetics, Inc., Marlow, UK) using 3 animals per group.

Tissue processing and electron microscopy (EM)

Hypothalamo-pituitary tissue blocks were fixed by immersion in 3% paraformaldehyde/0.05% paraformaldehyde in 0.1M phosphate buffer (pH 7.2) for 24 hours at room temperature and transferred to a 1:10 dilution of the fixative in 0.1M phosphate buffer for storage at 4°C before processing. Using a scalpel blade, areas from the medial PT and median eminence were cut into 0.5mm^3 pieces which were then stained with osmium (1% in 0.1M phosphate buffer), uranyl acetate (2% w/v in distilled water), dehydrated through increasing concentration of ethanol (70 to 100%), followed by 100% acetone and embedded in Spurr's resin (TAAB laboratory equipment, Aldermarston, UK). Ultrathin sections (50-80 nm) were prepared using a Reichart-Jung Ultracut ultramicrotome and mounted on nickel grids (Agar Scientific Ltd., Stanstead, UK). Sections were then counterstained with lead citrate and uranyl acetate and examined on a JOEL 1010 transmission electron microscope (JOEL USA Inc., Peabody, MA, USA). Sections from 3 animals per group were examined.

Quantitative electron microscopy morphological studies

For analysis of PT cell morphology, twenty micrographs per animal (n=3 sheep per group) of individual PT cells were taken at a magnification of x 5,000. Negatives were scanned into Adobe Photoshop CS2 (Adobe Corp., San Jose, CA, USA) and analysed using Axiovison version 4.5 (Zeiss, Oberkochen, Germany) image analysis software. The analyst was blind to the sample code. The following parameters were calculated: cytoplasmic, nuclear and total cell areas; granule area, granule areal density (granule area divided by cytoplasmic area x100 to express as %) and granule diameter. For measurement of the cell and nuclear areas, margins were drawn around the cell or nucleus respectively and the area was calculated. Cytoplasmic area was determined by subtracting nuclear area from total cell area. Granule number was counted and granule density was calculated by dividing total granule area by cytoplasmic area. Expansion of the rough endoplasmic reticulum (rough ER) and Golgi apparatus was assessed visually and graded on a scale of 0-4 (0, no expansion; 4 the most expansion). These estimates do not provide absolute measurements but do provide a basis for comparison.

Three measurements were made of the external zone of the median eminence using Axiovision from micrographs taken at x2500. 20 micrographs per sheep were analysed as follows. (1) The distance from the ends of nerve terminals to the basal lamina was measured. (2) In an area of 800$µm^2$ the area occupied by tanycytes was measured and expressed as a % of the total area. (3) The % of terminals that made contact with the basal lamina was counted in a 100µm region of external ME. Tanycytes were identified using criteria described by Ganten & Pfaff [S11]. All morphometric values represent the mean ± SEM (n=3 sheep per group). Means were compared by one way analysis of variance (ANOVA) with *post hoc* analysis by the Bonferroni test. $P<0.05$ was considered statistically different.

RNA-seq Assays

The pars tuberalis was dissected to minimise inclusion of transition zone and median eminence, though complete isolation of the PT is difficult to achieve. The PT samples were snap frozen on dry ice and stored at -80C. RNA was extracted from the pars tuberalis using Qiagen's TissueLyser II and RNeasy tissue kit. The quality of the extracted RNA was assessed using the Agilent 2100 Bioanalyser; all RNA integrity numbers (RINs) were above 8, indicating that good quality RNA had been extracted. Poly-A selection was used.

The library preparation protocol was carried out according to the manufacturer's instructions by Edinburgh Genomics. The LP induction experiment (Exp. 2) was sequenced using the Illumina genome analyzer II with 100 nucleotide single end (SE) reads, whereas the photoperiodic effect over longer time periods (Exp. 4) was sequenced on the Illumina Hiseq 2500 with 100 nucleotide paired end (PE) reads. The difference in the sequencing platforms means that we did not directly compare these experiments; we only compared the differentially expressed (DE) gene lists after all statistical processing was complete to avoid platform biases.

The FASTQ files were mapped to the Ensembl release 78 sheep reference genome (Oar_v3.1) using Bowtie [S12]. Approximately 60% of reads generated were uniquely mapped. All data have been submitted to GEO under the accession GSE65901.

Bioinformatics and Gene expression analysis

In order to measure gene expression from mapped RNA-seq data, the BAM files from Bowtie mapping were sorted using SAMtools [S13]. Raw counts per gene were estimated by the Python script HTSeq count (http://www-huber.embl.de/users/anders/HTSeq/) using the Ensembl sheep reference genome 78. The raw counts per gene were used by EdgeR [S14] to estimate differential expression (DE).

EdgeR (Bioconductor release 2.9) uses a pair-wise design to measure differential gene expression. The analysis is based on a negative binomial model that uses over-dispersion estimates to account for biological variability (i.e. sample to sample differences); this is an alternative to the Poisson estimates of biological variability that are often inappropriate [S15]. Counts per million (cpm) were calculated and only genes with 1 cpm in at least 3 samples was included in the analysis. Trimmed mean of M-values (TMM) normalisation of the sequenced libraries was performed to remove effects due to differences in library size [S14–S17]. EdgeR generates a fold change for each gene, p values and the Benjamin-Hochberg false discovery rate (FDR) are calculated to statistically test the measured DE. As in previous studies, no effect size cut-off was set [S18].

Enrichment analysis of GO terms

To assess the biological significance of gene expression changes across all the datasets we combined all the DE genes, removed DE non-coding RNAs, assigned GO terms to each of the genes and then sorted them by shared terms and log fold changes to create a heat-map.

To assess these datasets in a more stringent manner we used cytoscape plug-in ClueGO to perform an enrichment analysis on each individual comparison and then using a built-in algorithm the GO terms were collapsed based on related terms and statistical significance in order to give a simplified network [S19, S20]. Further enrichment analysis was performed by GSEA [S21, S22], consensusPathDB [S23] and DAVID [S24].

qPCR validation

To generate cDNA for qPCR, 2 µg of total RNA was reverse transcribed using the applied biosciences cDNA synthesis kit. The Roche universal probe library designer was used to design primers (https://www.roche-appliedscience.com/servlet/) with sequences obtained from Ensembl. All primers were designed to cross an exon–exon boundary. The specificity of the primers was checked using BLAST (http://www.ncbi.nlm.nih.gov/BLAST/). A reference gene experiment was conducted to identify the most stably expressed genes in the pars tuberalis (data not shown); HPRT1 and YWHAZ were the most stably expressed and were used to normalise the qPCR results. The qPCR

assays were all performed in duplicate using a TaqMan™ ABI PRISM 7500 fast (Applied Biosystems, Foster City, CA, USA) in 96-well plate format. A 20-ml reaction volume was used per well, consisting of: 10 µl Taqman 2× PCR master mix (Universal PCR Mastermix; Applied Biosystems),

0.2 µl each of 20 mM forward and reverse primers, 0.2 µl of 10 mM probe, 0.2 µl distilled water and 9.2 µl of cDNA or water for the negative controls. The amplification was performed as follows: 2 min at 50°C, 10 min at 95°C followed by 40 cycles of 95°C for 15 s and 60°C for 1 min. The efficiency of the assays were between 93 % and 107 % and the R2 values were >0.98. The ΔΔcT method was used to measure expression; "SP 4 weeks" was used as the reference samples from which relative expression was calculated. The data were further corrected by the efficiency of the standard curve for each gene. Log2 fold change relative to "SP 4 weeks" was calculated and compared to the RNA-seq results in order to confirm the expression results. The standard error was calculated for log2 fold change as follows: (std error/mean)*log2e. For qPCR the relative quantification values were used to calculate standard error. For RNA-seq, raw reads converted into relative values were used to calculate standard errors.

References

S1. Pelletier, J., Counis, R., de Reviers, M. M., Moumni, M., and Tillet, Y. (1995). Changes in LHbeta-gene and FSHbeta-gene expression in the ram pars tuberalis according to season and castration. Cell Tissue Res. *281*, 127–33.

S2. Pelletier, J., Counis, R., de Reviers, M. M., and Tillet, Y. (1992). Localization of luteinizing hormone beta-mRNA by in situ hybridization in the sheep pars tuberalis. Cell Tissue Res. *267*, 301–6.

S3. Gross, D. S. (1984). The mammalian hypophysial pars tuberalis: a comparative immunocytochemical study. Gen. Comp. Endocrinol. *56*, 283–98.

S4. Ikegami, K., Liao, X.-H., Hoshino, Y., Ono, H., Ota, W., Ito, Y., Nishiwaki-Ohkawa, T., Sato, C., Kitajima, K., Iigo, M., et al. (2014). Tissue-Specific Posttranslational Modification Allows Functional Targeting of Thyrotropin. Cell Rep. *9*, 801–809.

S5. Weintraub, B. D., Stannard, B. S., Magner, J. A., Ronin, C., Taylor, T., Joshi, L., Constant, R. B., Menezes-Ferreira, M. M., Petrick, P., and Gesundheit, N. (1985). Glycosylation and posttranslational processing of thyroid-stimulating hormone: clinical implications. Recent Prog. Horm. Res. *41*, 577–606.

S6. Dardente, H., Wyse, C. A., Birnie, M. J., Dupré, S. M., Loudon, A. S. I., Lincoln, G. A., and Hazlerigg, D. G. (2010). A molecular switch for photoperiod responsiveness in mammals. Curr. Biol. *20*, 2193–8.

S7. Dupré, S. M., Miedzinska, K., Duval, C. V, Yu, L., Goodman, R. L., Lincoln, G. A., Davis, J. R. E., McNeilly, A. S., Burt, D. D., and Loudon, A. S. I. (2010). Identification of Eya3 and TAC1 as long-day signals in the sheep pituitary. Curr. Biol. *20*, 829–35.

S8. McNeilly, A. S., and Andrews, P. (1974). Purification and characterization of caprine prolactin. J. Endocrinol. *60*, 359–67.

S9. Norton, A. J., Jordan, S., and Yeomans, P. (1994). Brief, high-temperature heat denaturation (pressure cooking): a simple and effective method of antigen retrieval for routinely processed tissues. J. Pathol. *173*, 371–9.

S10. Tóth, Z. E., and Mezey, E. (2007). Simultaneous visualization of multiple antigens with tyramide signal amplification using antibodies from the same species. J. Histochem. Cytochem. *55*, 545–54.

S11. Ganten, D., and Pfaff, D. (1986). Morphology of hypothalamus and its connections. In Current topics in Neuroendocrinology (Berlin: Springer Verlag).

S12. Langmead, B., Trapnell, C., Pop, M., and Salzberg, S. L. (2009). Ultrafast and memory-efficient alignment of short DNA sequences to the human genome. Genome Biol. *10*, R25.

S13. Li, H., Handsaker, B., Wysoker, A., Fennell, T., Ruan, J., Homer, N., Marth, G., Abecasis, G., Durbin, R., and Data, G. P. (2009). The Sequence Alignment/Map format and SAMtools. Bioinformatics *25*, 2078–9.

S14. Robinson, M., Chen, Y., Mccarthy, D., and Smyth, G. K. (2010). edgeR : differential expression analysis of digital gene expression data How to get help Reading data General comments. Most.

S15. Oshlack, A., Robinson, M. D., and Young, M. D. (2010). From RNA-seq reads to differential expression results. Genome Biol. *11*, 220.

S16. Robinson, M. D., and Oshlack, A. (2010). A scaling normalization method for differential expression analysis of RNA-seq data. Genome Biol. *11*, R25.

S17. Robinson, M., Mccarthy, D., Chen, Y., and Smyth, G. K. (2011). edgeR : differential expression analysis of digital gene expression data User ' s Guide.

S18. Wood, S. H., Craig, T., Li, Y., Merry, B., and de Magalhães, J. P. (2013). Whole transcriptome sequencing of the aging rat brain reveals dynamic RNA changes in the dark matter of the genome. Age (Dordr). *35*, 763–76.

S19. Smoot, M. E., Ono, K., Ruscheinski, J., Wang, P.-L., and Ideker, T. (2011). Cytoscape 2.8: new features for data integration and network visualization. Bioinformatics *27*, 431–2.

S20. Bindea, G., Mlecnik, B., Hackl, H., Charoentong, P., Tosolini, M., Kirilovsky, A., Fridman, W.-H., Pagès, F., Trajanoski, Z., and Galon, J. (2009). ClueGO: a Cytoscape plug-in to decipher functionally grouped gene ontology and pathway annotation networks. Bioinformatics *25*, 1091–3.

S21. Subramanian, A., Tamayo, P., Mootha, V. K., Mukherjee, S., Ebert, B. L., Gillette, M. A., Paulovich, A., Pomeroy, S. L., Golub, T. R., Lander, E. S., et al. (2005). Gene set enrichment analysis: a knowledge-based approach for interpreting genome-wide expression profiles. Proc. Natl. Acad. Sci. U. S. A. *102*, 15545–50.

S22. Mootha, V. K., Lindgren, C. M., Eriksson, K.-F., Subramanian, A., Sihag, S., Lehar, J., Puigserver, P., Carlsson, E., Ridderstråle, M., Laurila, E., et al. (2003). PGC-1alpha-responsive genes involved in oxidative phosphorylation are coordinately downregulated in human diabetes. Nat. Genet. *34*, 267–73.

S23. Kamburov, A., Stelzl, U., Lehrach, H., and Herwig, R. (2013). The ConsensusPathDB interaction database: 2013 update. Nucleic Acids Res. *41*, D793–800.

S24. Huang, D. W., Sherman, B. T., and Lempicki, R. a (2009). Systematic and integrative analysis of large gene lists using DAVID bioinformatics resources. Nat. Protoc. *4*, 44–57.
