# Supplementary material for: Binary Switching of Calendar Cells in the Pituitary Defines the Phase of the Circannual Cycle in Mammals
Source: Curr Biol. 2015 Oct 19;25(20):2651–62. doi: 10.1016/j.cub.2015.09.014 (PMC4612467; doi:10.1016/j.cub.2015.09.014)
Supplement: Document S2. Article plus Supplemental Information [file mmc4.pdf]

Article

Current Biology

Binary Switching of Calendar Cells in the Pituitary Defines the Phase of the Circannual Cycle in Mammals

Graphical Abstract

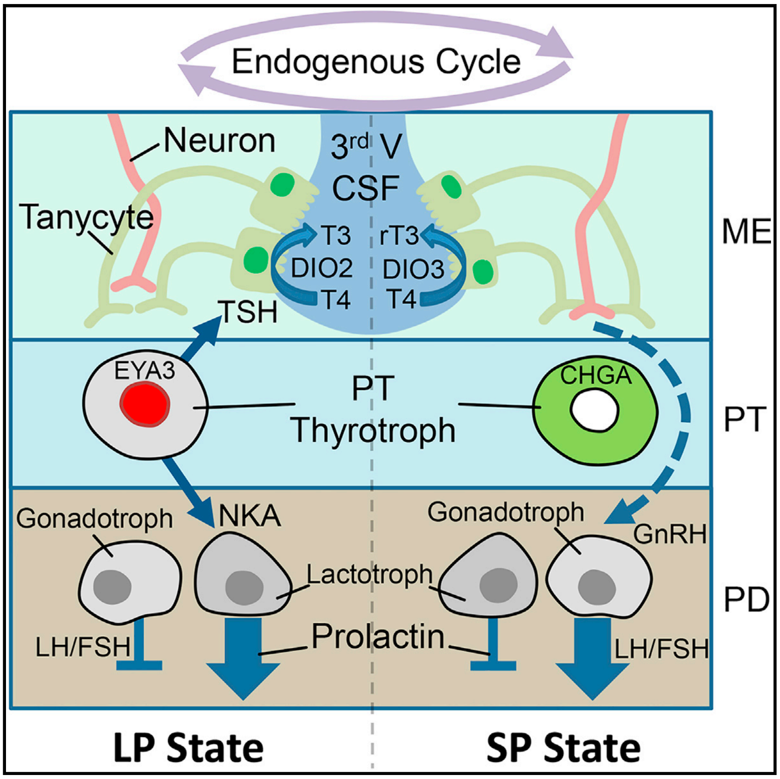

Highlights

- A circannual timer may reside in the pituitary pars tuberalis thyrotroph
- This is defined by a digital switching mechanism controlling EYA3 expression
- The circannual clockwork drives a morphogenic cycle in the PT and hypothalamus
- This involves recapitulation of a developmental program

Authors

Shona H. Wood, Helen C. Christian, Katarzyna Miedzinska, ..., Alan S. McNeilly, David W. Burt, Andrew S.I. Loudon

Correspondence

dave.burt@roslin.ed.ac.uk (D.W.B.), andrew.loudon@manchester.ac.uk (A.S.I.L.)

In Brief

Circannual rhythms have evolved to regulate and time annual changes in physiology. Wood et al. report that the pars tuberalis generates the circannual rhythm in mammals through the digital switching of EYA3 expression. A recapitulated developmental pathway is used by the circannual clock to drive a morphogenic cycle in the PT and hypothalamus.

Accession Numbers

GSE65901

Wood et al., 2015, Current Biology *25*, 2651–2662
October 19, 2015 ©2015 The Authors
http://dx.doi.org/10.1016/j.cub.2015.09.014

Current Biology
Article

Binary Switching of Calendar Cells in the Pituitary Defines the Phase of the Circannual Cycle in Mammals

Shona H. Wood,[1] Helen C. Christian,[2] Katarzyna Miedzinska,[3] Ben R.C. Saer,[1] Mark Johnson,[2] Bob Paton,[3] Le Yu,[3] Judith McNeilly,[4] Julian R.E. Davis,[5] Alan S. McNeilly,[4] David W. Burt,[3,*] and Andrew S.I. Loudon[1,*]
[1]Faculty of Life Sciences, University of Manchester, Manchester M13 9PT, UK
[2]Department of Physiology, Anatomy, and Genetics, Le Gros Clark Building, University of Oxford, South Parks Road, Oxford OX1 3QX, UK
[3]The Roslin Institute and Royal (Dick) School of Veterinary Studies, University of Edinburgh, Roslin, Midlothian EH25 9PRG, UK
[4]MRC Centre for Reproductive Health, Queen's Medical Research Institute, Edinburgh EH16 4TJ, UK
[5]Faculty of Medical and Human Science, University of Manchester, Manchester, M13 9PT, UK
*Correspondence: dave.burt@roslin.ed.ac.uk (D.W.B.), andrew.loudon@manchester.ac.uk (A.S.I.L.)
http://dx.doi.org/10.1016/j.cub.2015.09.014
This is an open access article under the CC BY license (http://creativecommons.org/licenses/by/4.0/).

SUMMARY

Persistent free-running circannual (approximately year-long) rhythms have evolved in animals to regulate hormone cycles, drive metabolic rhythms (including hibernation), and time annual reproduction. Recent studies have defined the photoperiodic input to this rhythm, wherein melatonin acts on thyrotroph cells of the pituitary pars tuberalis (PT), leading to seasonal changes in the control of thyroid hormone metabolism in the hypothalamus. However, seasonal rhythms persist in constant conditions in many species in the absence of a changing photoperiod signal, leading to the generation of circannual cycles. It is not known which cells, tissues, and pathways generate these remarkable long-term rhythmic processes. We show that individual PT thyrotrophs can be in one of two binary states reflecting either a long (EYA3$^+$) or short (CHGA$^+$) photoperiod, with the relative proportion in each state defining the phase of the circannual cycle. We also show that a morphogenic cycle driven by the PT leads to extensive re-modeling of the PT and hypothalamus over the circannual cycle. We propose that the PT may employ a recapitulated developmental pathway to drive changes in morphology of tissues and cells. Our data are consistent with the hypothesis that the circannual timer may reside within the PT thyrotroph and is encoded by a binary switch timing mechanism, which may regulate the generation of circannual neuroendocrine rhythms, leading to dynamic re-modeling of the hypothalamic interface. In summary, the PT-ventral hypothalamus now appears to be a prime structure involved in long-term rhythm generation.

INTRODUCTION

A complex repertoire of adaptive physiological cycles has evolved in vertebrates as a critical survival strategy for life in seasonal environments. These cycles are normally entrained by annual changes in day length (photoperiod), allowing animals to use a predictable changing signal to initiate complex physiological changes over the course of the year [1, 2]. This photoperiodic mechanism is known to entrain an underlying circannual clock, in which endogenous long-term free-running rhythms of physiology and behavior are tuned by an environmental signal. Circannual (approximately year-long) cycles are dominant features of the seasonal biology of mammals; they drive processes such as hibernation, metabolism, fattening, and reproductive activity [1–7] and persist when animals are kept in constant fixed photoperiods for many years [8–10].

The photoperiodic mechanism in vertebrates is known to involve seasonal regulation of thyroid hormones (THs), mediated in mammals via specialized cells (thyrotrophs) in the pituitary pars tuberalis (PT). It is not established whether this TH-input mechanism is also involved in the generation of long-term circannual cycles or whether separate molecular pathways and anatomical sites are involved. To address this, we examined the role of the circadian-controlled transcriptional co-activator EYA3 as the key upstream regulator of photoperiodic responses. Classic early studies [11] proposed that the circadian clock is co-opted for photoperiodic time measurement (external co-incidence hypothesis). It is posited that the core circadian clockwork drives a rhythmic circadian-regulated output gene(s), which then regulates a seasonal reproductive response. EYA3 is a strong candidate in mammals for this critical circadian-regulated gene [12]. A current model proposes that the rhythmic melatonin signal sets a local circadian oscillation in the PT via induction of the clock gene Cry1 [1, 13–15]. On short winter photoperiods (SPs), the peak phase of EYA3 is 12 hr after dark onset, coincident with nocturnal melatonin, which suppresses EYA3 expression [16]. On long photoperiods (LPs), the phase of expression is now coincident with light, and the gene is de-inhibited. This leads to co-activation of the TSHβ promoter in the PT thyrotoph, driving seasonal ependymal de-iodinase enzyme (DIO) signaling and TH metabolism [12]. Two de-iodinase enzyme genes (DIO2 and DIO3) reciprocally regulate to determine the local concentrations of the biologically active form of TH, triodothyronine (T3). TSHβ stimulation of DIO2 on LPs elevates T3 in specific neural sites, and this change has been shown experimentally to activate

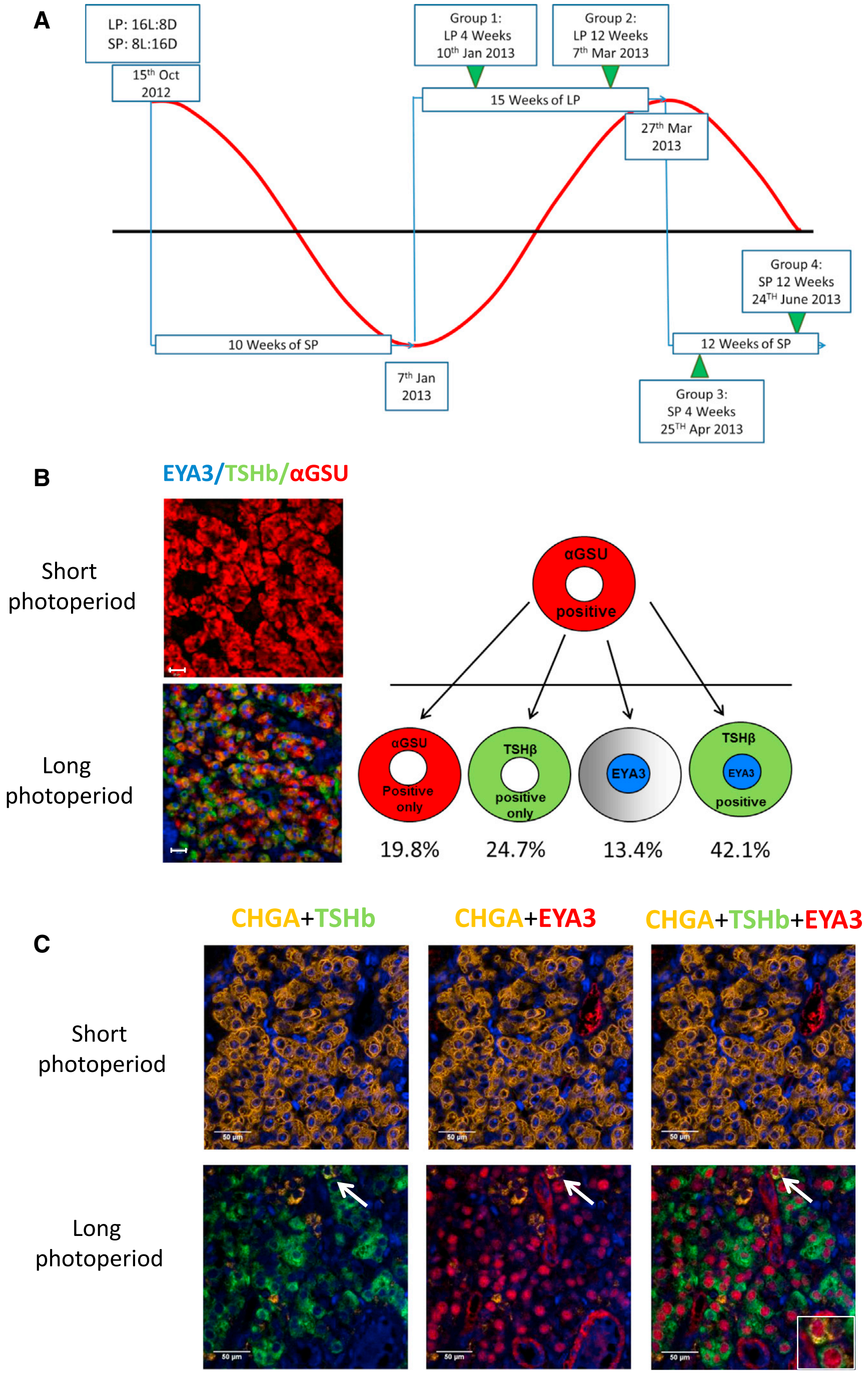

Figure 1. Co-localization of EYA3 and TSHβ to the PT Thyotroph on LPs and Identification of CHGA as a SP Marker

(A) Diagram of the photoperiodic treatment for experiment 1. Short photoperiod (SP): 8 hr light, 16 hr dark; long photoperiod (LP), 16 hr light, 8 hr dark. Tissues were collected at 4 and 12 weeks in SPs and LPs, in the mid-light phase, zeitgeber time (ZT; time after lights on): ZT4 in SPs and ZT8 in LPs. Collection points are represented by green arrows. The red line illustrates the natural photoperiod, and the blue lines represent the photoperiod imposed in light-controlled rooms (Figure S1A).

(B) Triple immunofluorescence showing expression of αGSU (red), TSHβ (green), and EYA3 (blue) in the PT on SPs and LPs (Figure S2). Scale bars, 20 μm. Quantification of EYA3 and TSHβ co-expression (Table S1 and Figure S3B) is shown as a schematic representing the variety of phenotypes that αGSU-expressing cells show in response to LPs.

(C) Triple immunofluorescence showing expression of EYA3 (red), CHGA (yellow), and TSHβ (green) in the PT on SPs and LPs (blue, DAPI) (Figure S3C and Table S3). Scale bars, 50 μm. The white arrow shows a cell co-expressing EYA3, CHGA, and TSHβ; this is one of two cells found in over 17,000 that co-express all three proteins.

See also Figures S1–S3.

a summer-type physiology. The nature of the neuroendocrine cascade triggered by the summer brain T3 has been extensively reviewed recently (in birds and mammals [17, 18], bony fish [19], and seasonal hypothalamic relays [1, 20]), thus linking a proposed circadian mechanism to a seasonal output.

Importantly, we now show that the EYA3/TSHβ relay is not just a “slave” to photoperiod, but may be centrally involved in circannual timing. The PT operates a novel binary switching mechanism in which the relative proportion of individual EYA3-expressing cells reflects the circannual phase. We also reveal long-term morphogenic changes in neuroendocrine tissues and extensive remodeling of developmental programs over the circannual cycle. The PT is thus a prime candidate site in mammals for the generation of circannual rhythms.

RESULTS

Expression of EYA3 and Chromogranin Protein in the PT

We characterized photoperiod-regulated proteins in the PT, using tissues collected from castrate male sheep maintained in controlled light-dark cycles, following exposure to LP or SP conditions (Figures 1A and S1A). To define photoperiod-responsive cell types in the PT, we screened with antibodies raised against all of the major pars distalis (PD) endocrine cell types (Figure S2). In the PT, we only detected the common subunit αGSU, characteristic of thyrotroph cells, despite previous studies reporting a gonadotroph population in the PT [21–25] (Figure S2A). The other major cell type was $S100^+$ cells (folliculostellate [FS] cells; Figure S3A). The PT is heavily vascularized, with arterioles and venules, but this gross structure and morphology did not change with photoperiod (Figure S3A). In the PT, on LPs, TSHβ protein was strongly co-expressed with αGSU-expressing cells (Figure 1B) but was non-detectable on SPs. In contrast, TSHβ protein did not change with photoperiod in the main PD thyrotroph population (Figure S2B).

We raised a novel ovine-specific EYA3 antibody (Supplemental Experimental Procedures), which revealed strong LP-specific co-localization of EYA3 to the αGSU-expressing "putative" PT thyrotroph (ZT4; Figure 1B). This supports a model for EYA3 co-activation of TSHβ expression, specifically within the putative PT thyrotroph [12, 16, 26]. To quantify EYA3 and TSHβ co-expression, we screened >4,000 αGSU^+ cells from LP PT tissue (Table S1). We detected cells with αGSU alone (19.8% of cells), as well as $TSH\beta/\alpha GSU^+$ (24.7%), $EYA3/\alpha GSU^+$ (13.4%), and $TSH\beta/EYA3/\alpha GSU^+$ (42.1%) cells (Figures 1B and S3B). Thus, EYA3 is induced in a subpopulation of putative PT thyrotrophs after 4 weeks of LP exposure. We were unable to detect EYA3 protein in thyrotrophs from SP-derived PT tissue (Figure 1B); however, low-level mRNA expression is detectable on SPs [12, 16].

We next conducted RNA-sequencing (RNA-seq) studies of the PT, comparing tissues between LPs and SPs (Figure S1A, experiments 2 and 4; GEO: GSE65901). This identified a number of genes increased under SPs (Table S2). In order to identify a robust SP marker, we compared from different conditions RNA-seq datasets and identified five consistent SP-marker genes (PAQR6, NPSR1, C1orf110, chromogranin A [CHGA], and SOX14). Only one, CHGA, was highly expressed, showing a 3-fold upregulation with SPs (Table S2). We screened >2,000 PT cells with a CHGA-specific antibody and showed that CHGA protein expression in the PT is exclusive to the putative PT thyrotroph (Figures S3C and S3D and Table S3). Thus, CHGA provides a robust SP marker for the activity of the putative PT thyrotroph. Using CHGA, TSHβ, and EYA3, antibodies we observed that CHGA expression was greatly enhanced after 4-week exposure to SPs and suppressed by LPs (Figure 1C). Of note, αGSU expression was unchanged (Figures S2A and S3E).

Thus, in the PT, EYA3 and CHGA are expressed specifically in putative PT thyrotroph cells and map to LP and SP conditions, respectively.

A Binary Cell-Based Timer over the Circannual Cycle

We next addressed whether these components exhibit long-term dynamic changes independently of photoperiod change. Many vertebrate species, including sheep [9], exhibit cycles of neuroendocrine activity when maintained on prolonged LPs [27, 28]. To achieve this, we kept cohorts of castrate male sheep (Figure S1A, experiment 3; born March/April) on natural photoperiods through to mid-October and then housed them in SP conditions for 12 weeks. Animals were then exposed to LPs for 29 weeks (LP29).

In order to track a robust endocrine output, which maps to the phase of the circannual cycle [7–10, 29], we developed a novel competitive ELISA to assay changing prolactin concentrations (Figure 2A and the Supplemental Experimental Procedures). Reversion to the SP-like state (the LP-refractory response) was defined as a drop in circulating prolactin concentrations to SP levels in an individual animal for 3 weeks or more. By this criterion, 77% of animals were in a LP-refractory state by week 27 (Figure 2A).

Quantification of *EYA3* mRNA expression by in situ hybridization showed a strong 5-fold induction at LP4, a decline to approximately 50% of peak values by LP16, and a reversion of PT expression to SP-like levels by LP29 (Figure 2B). *TSHβ* mRNA shows a similar trend, with a significant decline at LP29 (Figure 2B). These changes reflected the underlying endocrine prolactin rhythm.

We assessed expression of CHGA, TSHβ, and EYA3 protein in putative PT thyrotrophs at LP29, using the prolactin history of each animal to assess its relative circannual phase, and found that all had declined to low SP prolactin concentrations for at least 3 weeks prior to sampling (Figure S2C). The extent to which EYA3 and TSHβ protein declined and CHGA increased to a SP-like pattern appeared to be related to the length of time that an animal exhibited sustained periods of low prolactin concentrations (Figures 2C and S4A and Table S4). Thus, the EYA3 expression pattern reflects the endogenous endocrine phase of individual animals and undergoes dynamic changes as they revert to a winter-like physiology in the LP-refractory state.

Critically, quantification of >17,000 individual cells at SP4, LP4, and LP29 only identified two cells (0.01%) that co-expressed EYA3, TSHβ, and CHGA (Figure 2C and Table S4). As we had already established that αGSU expression was unchanged (Figures S2A and S3E), we considered whether a specialized subset of αGSU-expressing cells might define either the LP or SP phenotype, respectively. However, cell counts revealed that in SPs, virtually all (91.6%) αGSU-expressing cells were $CHGA^+$, whereas in LPs, 65.3% of αGSU-expressing cells were $EYA3^+$. Thus, we propose that the most likely explanation is that within a population of thyrotrophs, individual cells rapidly switch phenotype coding for either the SP ($CHGA^+/EYA3^-$) or LP ($EYA3^+/CHGA^-$) state, with the relative proportion of these cell types changing over the circannual cycle.

Photoperiod Drives a Morphogenic Transcriptional Program in the PT

We next set out to establish the full extent of transcriptional reprogramming within the PT, in response to acute changes after transfer from SP to LP conditions (Figure S1A, experiment 2). There were significant changes in expression at days 1, 7, and 28 of LPs, with an increasing transcriptional response with time (40, 269, and 424 genes, respectively; Table S2). Enrichment analysis using DAVID revealed that secreted signal, cell motion/adhesion, and cell-cell signaling were all enriched at days 1, 7, and 28 (Table S2). Approximately half of all genes changing in each comparison were shared, with an additional unique set of genes specific to each time point (Figure S4B). Therefore, there are continuous dynamic changes during acute LP exposure over 4 weeks.

We next addressed whether these dynamic changes occur over a longer timescale (4 and 16 weeks of SPs or 4 and 22 weeks of LPs; Figure S1A, experiment 4; GEO: GSE65901) and revealed profound transcriptional changes in response to exposure to fixed LPs or SPs (Figure S4B and Table S2). Within each photoperiod, there were marked dynamic changes in expression with an independent set of genes contingent on the duration of exposure (Figure S4B). For example, there were 251 genes differentially expressed (DE) for SP4/SP16 comparisons, with strong enrichment for secreted glycoprotein and cell adhesion (Table S2).

Although each time point was characterized by a unique subset of genes, we noted a marked similarity in function (Table S2).

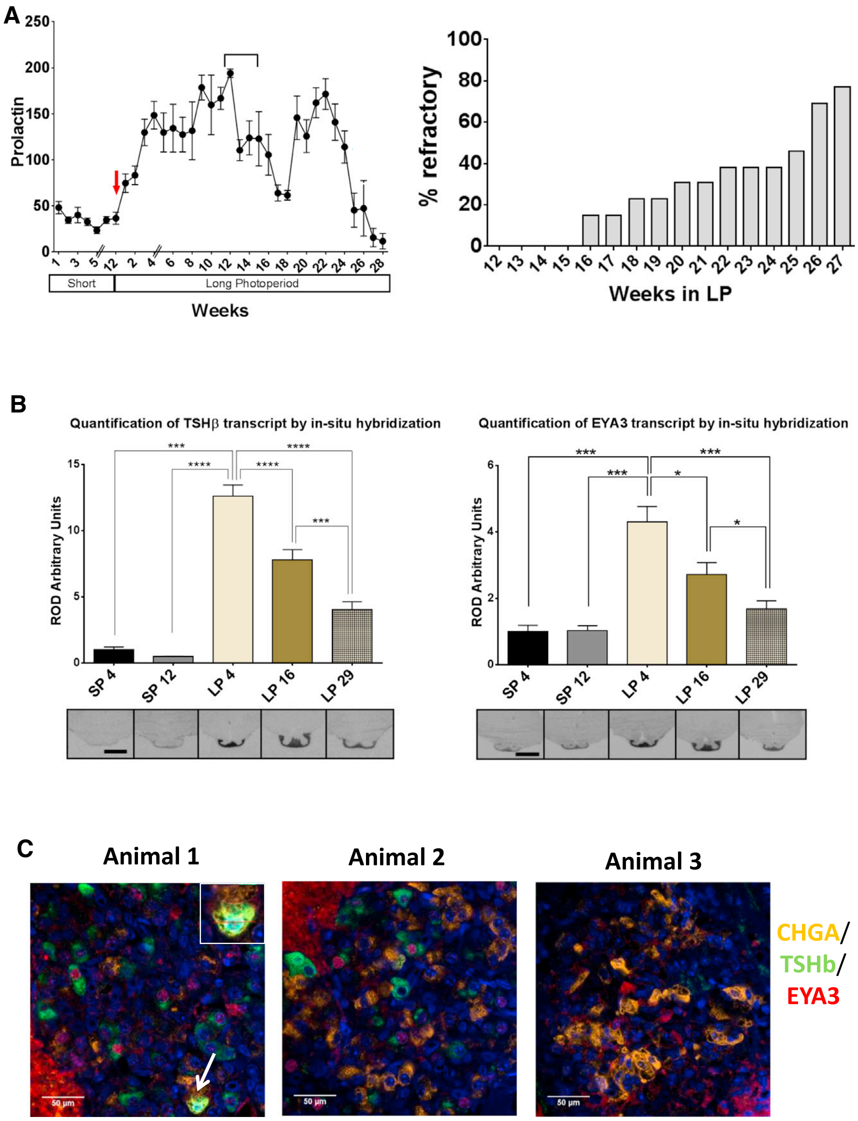

Figure 2. Binary Cell-Based Timing Mechanism over the Circannual Cycle

(A) Prolactin concentrations in plasma for 32 animals in SPs for 12 weeks transitioning into LPs for 4 weeks (red arrow marks this). The first double line in the graph indicates the gap in weeks between sampling during SPs, and the second double line represents a change in the cohort of animals sampled (as the previous group culled at 4 weeks). The dip in prolactin concentration between 13 to 18 weeks may be related to wool shearing (indicated on the graph by a bar spanning the affected period). The percentage of animals that show three consecutive weeks of suppressed prolactin (LP refractory) is shown. Error bars represent the SEM.

(B) In situ hybridization and quantification for TSHβ and EYA3 mRNA at SP4, SP12, LP4, LP16, and LP29. Representative images are shown (n = 3). Error bars represent the SD.

(C) Triple immunohistochemistry showing protein expression of EYA3 (red), TSHβ (green), CHGA (yellow), and DAPI (blue) at LP29 in three individuals (Figures S3C and S4A and Table S4). Scale bars, 50 μm.

See also Figures S2–S4.

To assess this, we combined the DE genes from all comparisons (experiments 2 and 4) and assigned Gene Ontology (GO) terms to create a simplified network of related statistically significant GO terms [30, 31] (Figure 3A) and a heatmap for all comparisons (Table S5). These data emphasize enrichment for development, morphogenesis, cell communication, movement, signaling, and hormones. Finally, we plotted the expression profiles for a select panel of genes falling within our enriched terms, showing dynamic regulation over all the time points. Of note are genes involved in axon guidance (KAL1, SEMA3D [33]) and a morphogen involved in pituitary and nervous system development (sonic hedgehog, SHH) [34] (Figure 3B). We validated these RNA-seq changes using qPCR (Figure S5A) and also show SHH expression in the putative PT thyrotrophs by immunohistochemistry (Figure S4C).

Since our RNA-seq data revealed a consistent pattern of changes implicating cellular re-modeling and development, we tested whether photoperiodic change might initiate cellular division in the PT, using two markers, Ki67 (Figure S5B) and phospho-histone H3 (Figure 3C), to identify de-novo mitotic events (experiment 3). A similar number of a few dividing cells (<0.2%) were detected in each photoperiod condition, but none were co-expressed with αGSU (Figures 3C and S5B), and we were unable to identify significant evidence of mitotic events. We next selected a panel of four well-established cell-cycle-control genes (CDK6, CDC45, CDC25c, and CDT1), but none revealed altered expression with photoperiod (Figure S6). As a further check, we mined the RNA-seq data for DE genes that shared the GO term category "cell proliferation"; in all cases, these genes were associated with dominant parent terms such as development or morphogenesis (Table S6). Thus, our RNA-seq analyses and subsequent histological studies failed to identify direct evidence for de novo seasonal histogenesis in the PT thyrotroph, but we did detect significant enrichment for photoperiod-regulated genes involved in cellular re-modeling. These underwent dynamic changes, both in response to acute changes in photoperiod and after prolonged exposure to both SP and LP conditions. The extent to which the extensive transcriptional changes that we report are driven by a common EYA3-dependent mechanism or other upstream transcriptional activators remains to be determined.

Cellular Re-modeling of the PT and Ventral Hypothalamus

The strong RNA-seq enrichment of developmental and morphogenic pathways suggested that a wider program of continuous re-modeling may occur. We therefore assessed ultrastructure of

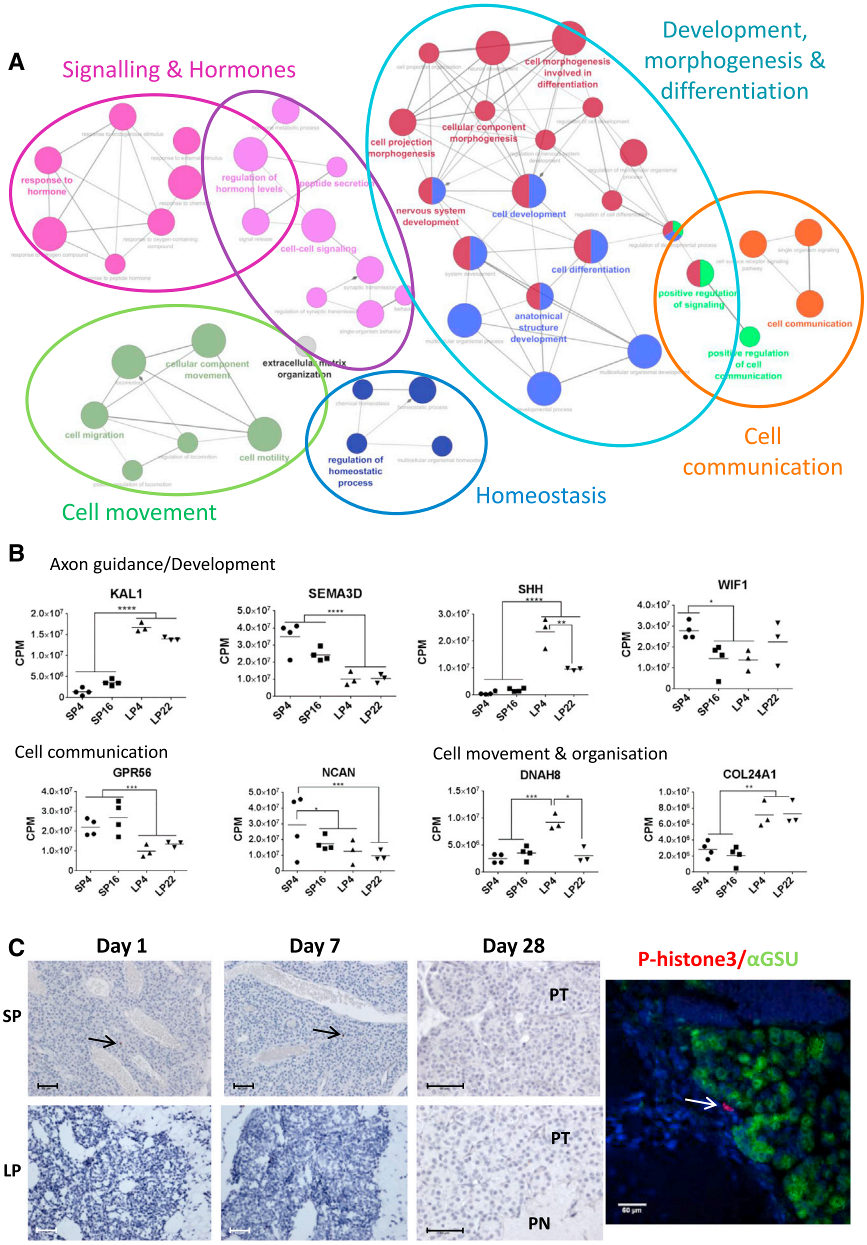

Figure 3. Transcriptional Response to Photoperiod and Histogenesis

(A) A simplified network of related statistically significant GO terms using the Cytoscape add-on ClueGO [30, 31]. The network comparing SP4 versus LP4 is shown. The filled colored circles (nodes) represent each statistically significant parent GO term. The lines (edges) between the nodes show that there are overlapping genes within each term. The colored ovals group these parent GO terms into more generic functional descriptions (Figure S4B).

(B) Graphs of average TMM normalized read counts per million (CPM) [32] from RNA-seq data from SP4, SP16, LP4, and LP22 (experiment 4). KAL1, SEMA3D, SHH, and WIF1 are within the following GO term categories: development, morphogenesis, and differentiation. GPR56 and NCAN are related to cell communication, and DNAH8 and COL2A1 are related to cell movement. False discovery rate (FDR)-corrected p values calculated by EdgeR are indicated as follows: *$p < 0.05$, **$p < 0.005$, ***$p < 0.0005$, and ****$p < 0.0001$ (Figures S4C and S5A).

(C) Detection of dividing cells by phospho-histone H3 (p-histone3) and hematoxylin staining in the sheep pars tuberalis (PT) and pars nervosa (PN) under SP and LP at days 1, 7, and 28. Arrows show dividing cells. Scale bars, 50 μm. Double immunofluorescence staining of αGSU (green), phospho-histone H3 (red), and DAPI (blue) in ovine PT is shown (Figures S5B and S6).

See also Figures S4–S6.

data are in line with the strong enrichment within the PT transcriptome for cell-to-cell communication (Figure 3).

We next assessed PT thyrotroph morphology. There was a significant increase in size after LP exposure, with >2- and 3-fold increases in area and volume, respectively (Figure 4B). However, by LP29, the morphology of the thyrotroph had reverted to a SP-like state. These photoperiodic changes were accompanied by a marked increase in the PT using electron microscopy (EM) at SP4, SP12, LP4, LP12, and LP29 (Figure S1A, experiments 1 and 3). This revealed substantial photoperiod-driven changes in morphology of both thyrotrophs and FS cells in the PT. In SP conditions (SP4), putative PT thyrotrophs were dispersed with relatively few cellular contacts, surrounded by a network of FS cells, forming close cellular contacts (Figure 4A). Exposure to LPs (LP4) caused tissue re-organization, with thyrotrophs forming close cell contacts and dispersal of FS cells. By LP29, the thyrotroph/FS cellular morphology reverted to SP-like morphology, with dispersed thyrotrophs and close FS cell contacts (Figure 4A). To quantify these differences, we scored the number of junctional contacts between FS and thyrotroph cells at SP4, LP4, and LP29 (Figure 4A). In each case, the pattern of changes reverted at LP29 to a SP-like morphology. These RER and Golgi on LPs (Figure 4C) and were inversely correlated with the density of secretory granule density, which was minimal at LP12 (Figure 4D). Staining for CHGA revealed strong co-localization to secretory granules on SPs (Figure S5C); however, immunogold labeling also revealed that CHGA was present in the cytoplasm and granules (data not shown). Again, these morphometric observations are supported by the RNA-seq results, which showed marked LP enrichment of genes involved in ER, Golgi, and vesicle formation (Table S5). Changes in RER, Golgi, secretory granules, and CHGA co-expression indicate an underlying complex and dynamic repertoire of secretory activities in these cells, which is significantly modified by photoperiod history. Importantly, these revert to a SP-like state in LP29 animals. Thus, the PT undergoes dynamic re-modeling over the seasonal cycle.

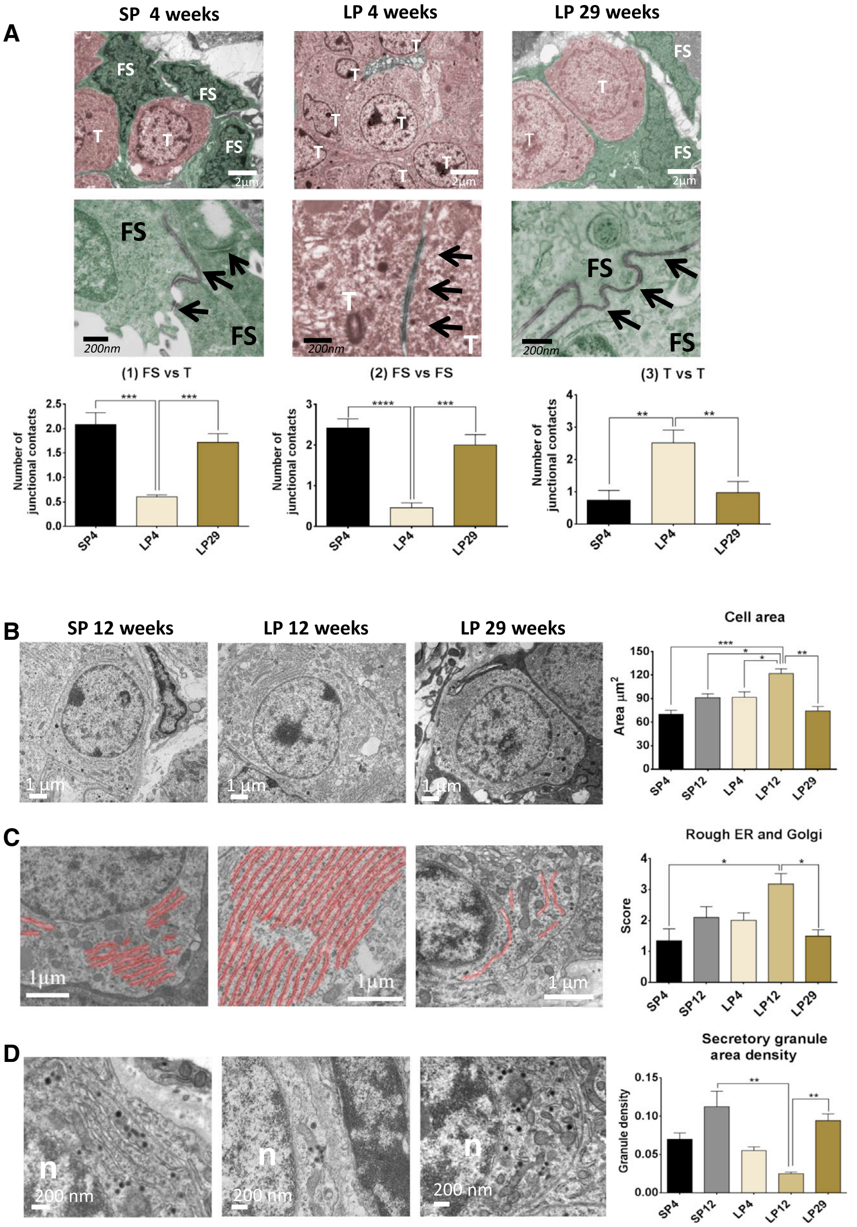

Figure 4. Cellular Remodeling in the PT

(A) EM images for LP4/SP 4 and LP29. FS, folliculostellate cells (green); T, thyrotroph (pink). Arrows indicate intercellular junctions. n = 3. Representative images are shown. Quantification of cell contacts is shown as follows: (1) thyrotroph/FS contacts, (2) FS/FS contacts, and (3) thyrotroph/thyrotroph contacts. Cell contacts were identified on the basis of electron dense morphology between cells at the plasma membrane. The morphology represents a potential mix of junctions—zona adherens, desmosomes, and gap junctions. One-way ANOVA was performed with multiple testing corrections, with adjusted p values as follows: $^*p < 0.05$, $^{**}p < 0.005$, $^{***}p < 0.0005$, and $^{****}p < 0.0001$; n = 3. Error bars represent the SEM.

(B) EM images of a PT thyrotroph at LP12, SP12, and LP29. Quantification of cell size is shown (μm^2). One-way ANOVA was performed with multiple testing corrections, with adjusted p values as follows: $^*p < 0.05$, $^{**}p < 0.005$, $^{***}p < 0.0005$, and $^{****}p < 0.0001$; n = 3. Error bars represent the SEM.

(C) Rough ER (RER) and Golgi false colored red and quantification of RER/Golgi. One-way ANOVA was performed with multiple testing corrections, with adjusted p values as follows: $^*p < 0.05$, $^{**}p < 0.005$, $^{***}p < 0.0005$, and $^{****}p < 0.0001$; n = 3. Error bars represent the SEM.

(D) Dark spots show the granules present and quantification of secretory areal granule density. In all cases, SP4, SP12, LP4, LP12, and LP29 are presented with n = 3; representative images are shown. One-way ANOVA was performed with multiple testing corrections, with adjusted p values as follows: $^*p < 0.05$, $^{**}p < 0.005$, $^{***}p < 0.0005$, and $^{****}p < 0.0001$. Error bars represent the SEM.

See also Figure S5.

The current model for photoperiodic regulation proposes that TSH of PT origin acts on adjacent ependymal cells in the hypothalamus to regulate the activity of the GnRH neurone [1, 35]. Seasonal changes in the neuro-glial interactions in the median eminence (ME) have been reported in the Japanese quail [36], but to date, there are no reports of similar changes in mammals. Vimentin staining revealed an extensive tanycyte network at the ME interface with the PT, which terminates in the basal lamina (Figure 5A). Staining for GnRH revealed a pattern of terminal fields within the tanycyte network. On LPs, there was a marked tanycyte barrier between GnRH terminals and the basal lamina (BL), with strong evident GnRH staining. In contrast, on SPs, GnRH staining extended through to the BL, and the tanycyte barrier was more dispersed. Further studies at the EM level revealed that there was a significant increase in contacts of the nerve terminals with the BL on SPs, whereas on LPs, the long tanycytic processes extended throughout the BL of the ME (Figures 5B and 5C). As a consequence, there was a highly significant reduction in the proportion of nerve terminals that made BL contact on LPs and a reciprocal increase in mean distance from the BL (Figure 5C). By LP29, these phenotypes had fully reverted to a SP state (Figure 5C). Thus, in addition to the substantial changes reported within the PT, we also reveal dynamic extensive re-modeling events at the neural-glial interface over the circannual cycle (Figure 6).

DISCUSSION

We employed the paradigm in a photoperiodic species in which the innate nature of the circannual timing mechanism is revealed by exposure to constant long day length over prolonged periods. Using this model, we addressed what role the photoperiodic relay mechanism might play in the initiation of a circannual rhythm. Two broad possible outcomes can be considered. First, the photoperiodic relay might faithfully record the environmental light cycle but does not contribute to circannual rhythm

generation. Second, the input relay may itself also be part of the circannual timing mechanism, in which case it would be predicted to change spontaneously over the circannual cycle and map closely to the underlying physiology.

We now show that EYA3 protein is strongly induced specifically in PT thyrotrophs by LPs. Using in situ hybridization, we also show that prolonged exposure (6 months) to LPs leads to a marked suppression of both EYA3 and TSHβ mRNA in the PT. Immunohistochemical studies revealed a dramatic reduction in the number of cells expressing both proteins in the PT, the extent of which relates to the history of prolactin secretion in individual photo-refractory animals. Thus, using prolactin as an endocrine marker for the underlying circannual phase [7–10], our data support a model in which the photoperiodic-input mechanism within the PT is involved in the generation of the circannual cycle. The PT is also directly involved in seasonal prolactin regulation via an intra-pituitary mechanism involving the PT production of a paracrine signal independently of neural input [8, 16, 37], the nature of which is known to change spontaneously in photo-refractory animals [38]. Remarkably, hypothalamo-pituitary surgical disconnection (HPD) of the pituitary from the hypothalamus does not disrupt seasonal or circannual control of prolactin rhythms, strongly implicating a non-neural autonomous local mechanism in the regulation of this hormone [8, 9].

Recent studies have revealed dynamic circannual changes in DIO regulation in the ventral hypothalamic tanycyte cells of Soay sheep and Siberian and European hamsters [6, 39, 40] and PT TSHβ mRNA in European hamsters and Soay sheep [6, 39]. As EYA3 is known to regulate TSHβ, this further implicates the PT thyrotroph as being centrally involved in circannual rhythm generation. The changes in EYA3 expression that we report are unlikely to be driven by alterations in the duration of the nocturnal melatonin signal, as this hormone continues to report the prevailing photoperiod, irrespective of circannual phase [10]. Nor is it likely that altered phasing of the core circadian clockwork is involved, as core clock genes (*Per1* and *2*, *Cry1*, *Reverbα*, and *Bmal1*) in the PT do not change phase in photo-refractory sheep [10, 41].

Using CHGA as a short-day marker within the putative PT thyrotrophs, we showed that both the mRNA and protein are strongly induced in response to SPs. CHGA is essential for the formation of dense-core secretory granules and for regulating the secretion of pro-hormones in the gonadotrophs and somatotrophs of the PD [42, 43], and it reflects the physiological state of the SP thyrotroph. Remarkably, we were only able to detect two cells (0.01%) co-expressing TSHβ, EYA3, and CHGA. This suggests that the PT may record photoperiod history in the form of a binary code within individual thyrotroph cells, which rapidly flips from either a LP or SP state, but cannot be in both. Accordingly, the relative proportion of cells in each state may determine the overall activity of the PT, and hence the phase of the circannual cycle, as the PT reverts to a winter-like state after prolonged LP exposure, the proportion of $CHGA^+$ cells increases as EYA3 and TSHβ decline. Whether the two processes are causally linked (i.e., decline in EYA3 directly initiates expression of CHGA) is not yet established. We are unaware of similar models driving seasonal biology in other animals; however, mathematical modeling has predicted a switching phenotype in response to photoperiod [44]. Furthermore, studies of vernalization mechanisms in plants propose a similar cell-based binary switching mechanism, which provides a memory of winter cold exposure and involves epigenetic control of a flowering repressor [45].

It is attractive to speculate that the dynamic continuous transcriptomic changes observed are driven by the changing ratios of cells expressing CHGA (SP) and EYA3 (LP). A conceptually similar mechanism exists in prolactin gene regulation within the rat anterior pituitary gland, in which the overall tissue response is generated by the activity of individual stochastically switching units [46].

A recent hypothesis has proposed that the circannual cycle may be generated by an underlying seasonal histogenesis in the PT, involving the reactivation of a latent stem cell population and an annual regenerative cycle [47–49]. We were unable to find evidence for significant de novo cell division. However, earlier studies have reported low level changes in cell division in the PT in response to photoperiod change; one reported an increase in division in LPs [49], but the other reported a decrease [48]. The latter study reported that these dividing cells were $CD45^+$, suggesting that infiltrating immune cells may be the source of these dividing cells [48]. In addition, a recent study identified a very small number of dividing cells in the mouse PT [50]. In our study, we were unable to assess the extent to which histogenesis might be involved in circannual rhythm generation, but the very small numbers of dividing cells detected were certainly not thyrotrophs. In contrast, our morphological studies revealed extensive re-modeling within the PT and the thyrotrophs over the circannual cycle, with the SP state being characterized by elevated CHGA, secretory granule formation, and storage of pro-hormones [51], and the LP state by a secretory phenotype (i.e., increases in RER). Changes in the morphology and exocytotic activity in the PT-specific thyrotrophs have been reported in seasonal Djungarian hamsters and hibernating hedgehogs [52–54]. Thus, the PT may employ a recapitulated developmental pathway to drive changes in morphology of tissues and cells, which does not necessarily extend to activation of a latent stem cell population. Similar mechanisms have been documented in anterior pituitary cells, with hyperplasic changes in response to hormone stimulus, independently of cell division [55]. In this regard, other members of the EYA family are known to be key developmental regulators of the retina and other tissues [56–58], with EYA3 being co-opted for photoperiodic time measurement in the PT.

Our data suggest that the PT may establish morphogenic gradients, which extend to the ME, leading to retrograde signaling, and seasonal re-modeling of neuronal terminals and the associated tanycyte end feet. We identified enrichment for genes involved in establishing morphogenic gradients for GnRH neuron guidance in the developing brain in the PT (KAL1 and SEMA3D) [33]. In this model, the PT may both regulate DIO signaling in the hypothalamus and also drive changes in neuroendocrine morphology. In laboratory rodents, the plexin/semphorin (SEMA) signaling system is tightly controlled by gonadal steroids, leading to extensive re-modeling of tanycyte/GnRH neurones over the oestrous cycle [59, 60]. Alterations in the access of GnRH nerve terminals to the basal lamina of the ME have been reported in hamsters maintained in constant darkness [61]. Seasonal changes in GnRH accessibility to the have been shown in Japanese quail [36] and are of a similar magnitude to

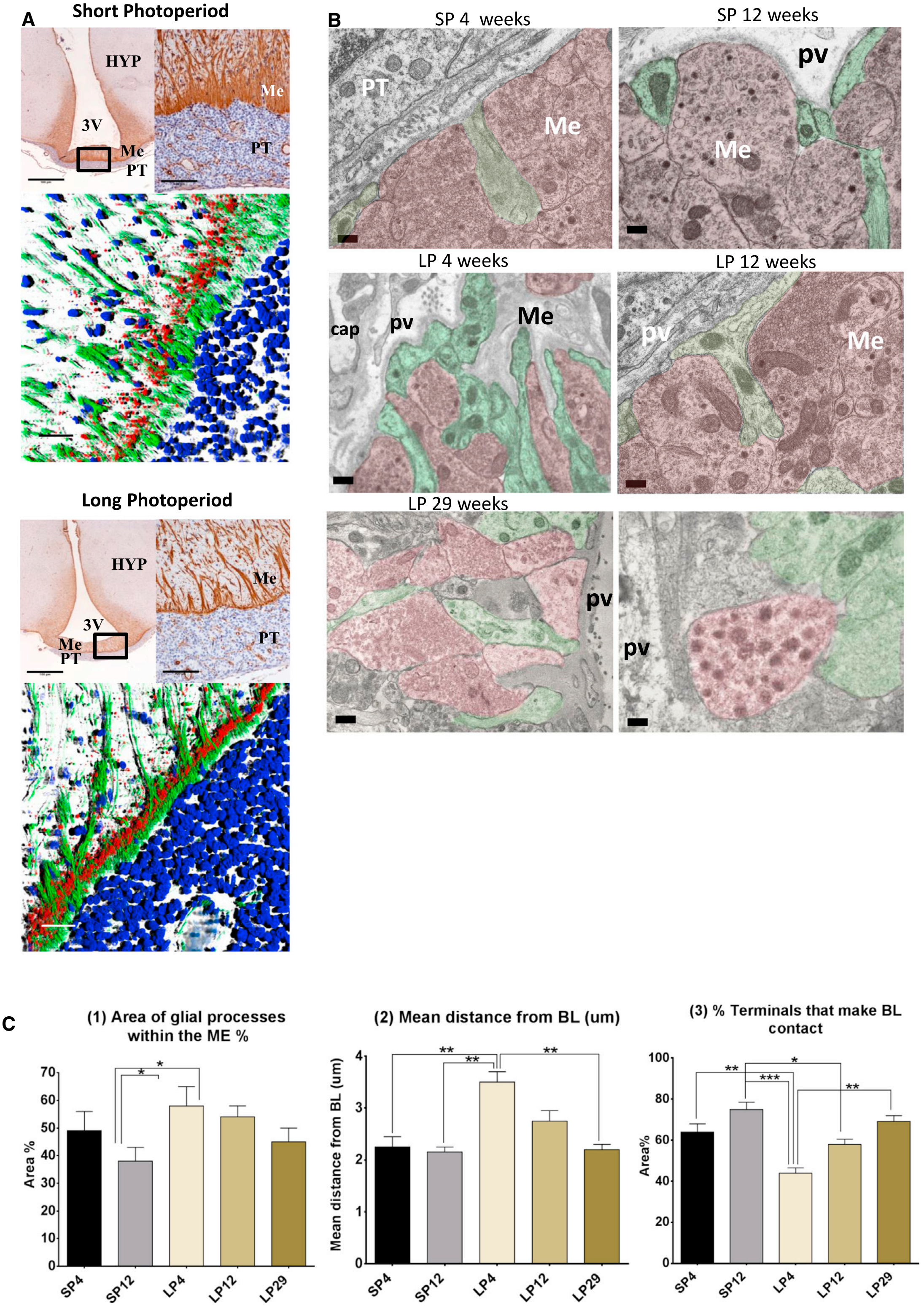

Figure 5. Remodeling of the Neural-Glial Interface of the Median Eminence

(A) Vimentin immunostaining for tanycytes (brown) of coronal section of the sheep mediobasal hypothalamus (top). Scale bars, 100 μm and 20 μm, respectively. PT, pars tuberalis; Me, median eminence; 3V, third ventricle; HYP, hypothalamus. 3D render series of IHC images showing GnRH (red), vimentin (green), and DAPI (blue) in SPs and LPs are also shown (bottom). Scale bar, 50 μm.

(legend continued on next page)

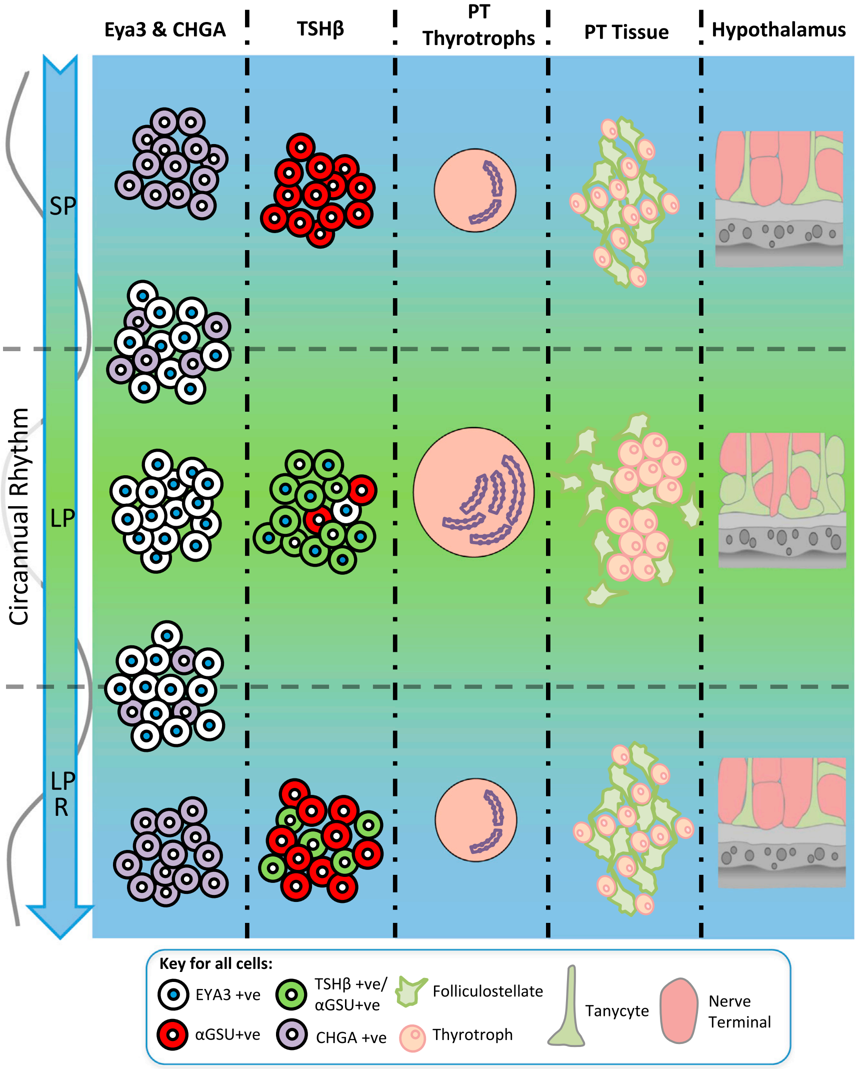

Figure 6. Summary of the Changes in the PT and Median Eminence throughout a Circannual Cycle

The model proposes that an endogenous timer switches EYA3 expression in the PT thyrotroph cells, driving TSH and hypothalamic thyroid hormone metabolism independently of melatonin. Individual PT thyrotroph cells are either in a long ($EYA3^{+}$) or short ($CHGA^{+}$) state, and the relative proportion of these binary-state cells determines the phase of the circannual cycle. Re-modeling of the morphology of the PT sees changes in cell size and RER. Within the PT, networks of either thyrotrophs (LP) or FS cells (SP) form. Re-modeling of the hypothalamic interface in the ME leads to encasement of neuronal synapses by tanycyte end feet in the non-breeding season (LP), suggesting a physical mechanism for control of GnRH secretion. Collectively, this suggests that the PT thyrotroph operates as a calendar cell, generating long-term neuroendocrine rhythms in both the hypothalamus and pituitary gland.

those we report here, but, in contrast to our data, this occurred on opposing photoperiods (i.e., tanycyte enclosure of neuronal synapses occurred on SPs in quail, rather than on LPs in sheep). Since the TSH/DIO/T3 hormone pathway is activated by LPs in a broadly similar manner in both seasonal birds and mammals [4, 62] irrespective of phase of breeding season, it would appear that local thyroid hormone changes cannot directly account for these differences. Intriguingly, this suggests that remodeling of the neuroendocrine synapse may be linked to the phase of the reproductive cycle rather than the prevailing photoperiod. Since our studies were undertaken in castrated animals, changes in circulating sex steroids are unlikely to be involved.

We propose that the PT thyrotroph acts as a seasonal calendar cell with the capacity to generate long-term rhythms in mammals, driving both hypothalamic and pituitary endocrine circuits (Figure 6). Our studies cannot exclude the possibility that other structures (i.e., ependymal tanycytes) might also be involved. However, we implicate the same molecular mechanisms involved in the photoperiodic readout in the generation of long-term circannual cycles. The discovery of a role for thyrotroph cells in seasonal photoperiodism in birds and salmonid fishes [19, 63] suggests that this cell type may have a hitherto-unexpected evolutionarily conserved general role both in seasonal endocrine control and circannual rhythm generation across the vertebrate class. In summary, the PT-ventral hypothalamus now appears to be a prime structure involved in long-term rhythm generation.

EXPERIMENTAL PROCEDURES

All studies involving animals were licensed by the appropriate UK regulatory authority (Animals, Scientific Procedures Act, 1986), under a project license held by A.L. and approved by the local ethics committee. Scottish blackface sheep were housed in artificial light/dark cycles—either an 8:16 hr light/dark cycle for SPs or a 16:8 hr light/dark cycle for LPs. All animals were killed by an overdose of barbiturate (Euthatal; Rhone Merieux) administered intravenously, and sampling time points varied; see the Supplemental Experimental Procedures and figures for details. Hypothalamic blocks with the PT and pituitary attached were collected for immunohistochemistry, EM, and transcriptomics. Bioinformatic analysis was performed using Bowtie, SAMtools, HTSeq

(B) EM images of ME nerve terminals (pink) and tanycytes (green) in SP4, SP12, LP4, LP12, and LP29. pv, perivascular space; cap, capillary. Scale bar, 200 nm. n = 3; representative images are shown.

(C) Quantification of the (1) percentage area of glial process (tanycytic end foot) within the ME, (2) mean distance of the nerve terminal from the basal lamina (μm), and (3) percentage of nerve terminals in contact with basal lamina. One-way ANOVA was performed with multiple testing corrections, with adjusted p values as follows: $^{*}p < 0.05$, $^{**}p < 0.005$, $^{***}p < 0.0005$, and $^{****}p < 0.0001$; n = 3. Error bars represent the SEM.

See also Figure S1.

count, EdgeR, GSEA, DAVID, and Cytoscape. Ovine prolactin (oPRL) was measured using a newly developed competitive ELISA using purified oPRL (NIDDK-oPRL-21, AFP10692C; from Dr. A. Parlow, National Hormone and Peptide Program, Harbor-UCLA) and a highly specific rabbit anti-ovine prolactin (ASM-R50; produced by ASM) used previously in the specific radioimmunoassay [64]. In situ hybridization was performed as previously described [12]. The *OaTSHβ* plasmid (NCBI Gene: XM_004002368.2) was kindly provided by David Hazlerigg. The *OaEya3* plasmid (NCBI Gene: NM_001161733.1) was cloned as previously described [12]. Full details are provided in the Supplemental Experimental Procedures.

ACCESSION NUMBERS

The accession number for the RNA-seq data reported in this paper is GEO: GSE65901.

SUPPLEMENTAL INFORMATION

Supplemental Information includes Supplemental Experimental Procedures, six figures, and six tables and can be found with this article online at http://dx.doi.org/10.1016/j.cub.2015.09.014.

AUTHOR CONTRIBUTIONS

S.W. designed the experiments, collected samples, performed bioinformatic analysis and qPCR, and prepared the manuscript. H.C. performed the EM and analysis. K.M. performed IHC and analysis. B.S. collected samples and performed the in situ hybridization. M.J. performed EM and analysis. B.P. performed RNA preparation and sequencing. L.Y. performed bioinformatic analysis. J.M. developed the novel prolactin assay and prepared antibodies. J.D. designed experiments and revised the manuscript. A.M. collected samples, designed experiments, developed the prolactin assay, and revised the manuscript. D.B. designed experiments and revised the manuscript. A.L. designed experiments, collected samples, and prepared the manuscript.

ACKNOWLEDGMENTS

Many past and current members of the Manchester/Edinburgh team have made contribution to discussions and offered advice, including David Bechtold, Sandrine Dupre, Louise Ince, Richard Kuo, and Alex West. In addition, the authors thank the staff at the Marshall Building, Roslin, Edinburgh for expert care of their research animals and Edinburgh Genomics. The work was supported by grants from the Biotechnology and Biological Sciences Research Council UK awarded to A.L. (BB/K003119/1) and D.B. (BB/K000764/1).

Received: May 29, 2015
Revised: August 11, 2015
Accepted: September 4, 2015
Published: September 24, 2015

REFERENCES

1. Wood, S., and Loudon, A. (2014). Clocks for all seasons: unwinding the roles and mechanisms of circadian and interval timers in the hypothalamus and pituitary. J. Endocrinol. *222*, R39–R59.
2. Ebling, F.J.P., and Barrett, P. (2008). The regulation of seasonal changes in food intake and body weight. J. Neuroendocrinol. *20*, 827–833.
3. Woodfill, C.J., Wayne, N.L., Moenter, S.M., and Karsch, F.J. (1994). Photoperiodic synchronization of a circannual reproductive rhythm in sheep: identification of season-specific time cues. Biol. Reprod. *50*, 965–976.
4. Dardente, H., Hazlerigg, D.G., and Ebling, F.J.P. (2014). Thyroid hormone and seasonal rhythmicity. Front. Endocrinol. (Lausanne) *5*, 19.
5. Monecke, S., Sage-Ciocca, D., Wollnik, F., and Pévet, P. (2013). Photoperiod can entrain circannual rhythms in pinealectomized European hamsters. J. Biol. Rhythms *28*, 278–290.
6. Sáenz de Miera, C., Monecke, S., Bartzen-Sprauer, J., Laran-Chich, M.-P., Pévet, P., Hazlerigg, D.G., and Simonneaux, V. (2014). A circannual clock drives expression of genes central for seasonal reproduction. Curr. Biol. *24*, 1500–1506.
7. Duncan, M.J. (2007). Circannual prolactin rhythms: calendar-like timer revealed in the pituitary gland. Trends Endocrinol. Metab. *18*, 259–260.
8. Lincoln, G.A., and Clarke, I.J. (1994). Photoperiodically-induced cycles in the secretion of prolactin in hypothalamo-pituitary disconnected rams: evidence for translation of the melatonin signal in the pituitary gland. J. Neuroendocrinol. *6*, 251–260.
9. Lincoln, G.A., Clarke, I.J., Hut, R.A., and Hazlerigg, D.G. (2006). Characterizing a mammalian circannual pacemaker. Science *314*, 1941–1944.
10. Lincoln, G.A., Johnston, J.D., Andersson, H., Wagner, G., and Hazlerigg, D.G. (2005). Photorefractoriness in mammals: dissociating a seasonal timer from the circadian-based photoperiod response. Endocrinology *146*, 3782–3790.
11. Bünning, E. (1936). Die endogene Tagesperiodik als Grundlage der photoperiodischen Reaktion. Ber. Dtsch. Bot. Ges. *54*, 590–608.
12. Dardente, H., Wyse, C.A., Birnie, M.J., Dupré, S.M., Loudon, A.S.I., Lincoln, G.A., and Hazlerigg, D.G. (2010). A molecular switch for photoperiod responsiveness in mammals. Curr. Biol. *20*, 2193–2198.
13. Dupré, S.M., Burt, D.W., Talbot, R., Downing, A., Mouzaki, D., Waddington, D., Malpaux, B., Davis, J.R.E., Lincoln, G.A., and Loudon, A.S.I. (2008). Identification of melatonin-regulated genes in the ovine pituitary pars tuberalis, a target site for seasonal hormone control. Endocrinology *149*, 5527–5539.
14. Hanon, E.A., Routledge, K., Dardente, H., Masson-Pévet, M., Morgan, P.J., and Hazlerigg, D.G. (2010). Effect of photoperiod on the thyroid-stimulating hormone neuroendocrine system in the European hamster (Cricetus cricetus). J. Neuroendocrinol. *22*, 51–55.
15. West, A., Dupré, S.M., Yu, L., Paton, I.R., Miedzinska, K., McNeilly, A.S., Davis, J.R., Burt, D.W., and Loudon, A.S. (2013). Npas4 is activated by melatonin, and drives the clock gene Cry1 in the ovine pars tuberalis. Mol. Endocrinol. *27*, 979–989.
16. Dupré, S.M., Miedzinska, K., Duval, C.V., Yu, L., Goodman, R.L., Lincoln, G.A., Davis, J.R.E., McNeilly, A.S., Burt, D.D., and Loudon, A.S.I. (2010). Identification of Eya3 and TAC1 as long-day signals in the sheep pituitary. Curr. Biol. *20*, 829–835.
17. Yasuo, S., Watanabe, M., Nakao, N., Takagi, T., Follett, B.K., Ebihara, S., and Yoshimura, T. (2005). The reciprocal switching of two thyroid hormone-activating and -inactivating enzyme genes is involved in the photoperiodic gonadal response of Japanese quail. Endocrinology *146*, 2551–2554.
18. Hanon, E.A., Lincoln, G.A., Fustin, J.-M., Dardente, H., Masson-Pévet, M., Morgan, P.J., and Hazlerigg, D.G. (2008). Ancestral TSH mechanism signals summer in a photoperiodic mammal. Curr. Biol. *18*, 1147–1152.
19. Nakane, Y., Ikegami, K., Iigo, M., Ono, H., Takeda, K., Takahashi, D., Uesaka, M., Kimijima, M., Hashimoto, R., Arai, N., et al. (2013). The saccus vasculosus of fish is a sensor of seasonal changes in day length. Nat. Commun. *4*, 2108.
20. Bolborea, M., and Dale, N. (2013). Hypothalamic tanycytes: potential roles in the control of feeding and energy balance. Trends Neurosci. *36*, 91–100.
21. Pelletier, J., Counis, R., de Reviers, M.M., Moumni, M., and Tillet, Y. (1995). Changes in LHbeta-gene and FSHbeta-gene expression in the ram pars tuberalis according to season and castration. Cell Tissue Res. *281*, 127–133.
22. Pelletier, J., Counis, R., de Reviers, M.M., and Tillet, Y. (1992). Localization of luteinizing hormone beta-mRNA by in situ hybridization in the sheep pars tuberalis. Cell Tissue Res. *267*, 301–306.
23. Gross, D.S. (1984). The mammalian hypophysial pars tuberalis: a comparative immunocytochemical study. Gen. Comp. Endocrinol. *56*, 283–298.

24. Skinner, D.C., and Robinson, J.E. (1996). The pars tuberalis of the ewe: no effect of season or ovariectomy on the distribution, density or presence of immunoreactive cells. Cell Tissue Res. *284*, 117–123.

25. Skinner, D.C., and Robinson, J.E. (1995). Melatonin-binding sites in the gonadotroph-enriched zona tuberalis of ewes. J. Reprod. Fertil. *104*, 243–250.

26. Masumoto, K., Ukai-Tadenuma, M., Kasukawa, T., Nagano, M., Uno, K.D., Tsujino, K., Horikawa, K., Shigeyoshi, Y., and Ueda, H.R. (2010). Acute induction of Eya3 by late-night light stimulation triggers TSHβ expression in photoperiodism. Curr. Biol. *20*, 2199–2206.

27. Gwinner, E. (1981). [Circannual rhythms in animals and their photoperiodic synchronization]. Naturwissenschaften *68*, 542–551.

28. Gwinner, E., and Dittami, J. (1990). Endogenous reproductive rhythms in a tropical bird. Science *249*, 906–908.

29. Brinklow, B.R., and Loudon, A.S. (1993). Evidence for a circannual rhythm of reproduction and prolactin secretion in a seasonally breeding macropodid marsupial, the Bennett's wallaby (Macropus rufogriseus rufogriseus). J. Reprod. Fertil. *98*, 625–630.

30. Smoot, M.E., Ono, K., Ruscheinski, J., Wang, P.-L., and Ideker, T. (2011). Cytoscape 2.8: new features for data integration and network visualization. Bioinformatics *27*, 431–432.

31. Bindea, G., Mlecnik, B., Hackl, H., Charoentong, P., Tosolini, M., Kirilovsky, A., Fridman, W.-H., Pagès, F., Trajanoski, Z., and Galon, J. (2009). ClueGO: a Cytoscape plug-in to decipher functionally grouped gene ontology and pathway annotation networks. Bioinformatics *25*, 1091–1093.

32. Robinson, M., Mccarthy, D., Chen, Y., and Smyth, G. K. (2011). edgeR: differential expression analysis of digital gene expression data user's guide. http://www.bioconductor.org/packages/release/bioc/vignettes/edgeR/inst/doc/edgeRUsersGuide.pdf

33. Messina, A., and Giacobini, P. (2013). Semaphorin signaling in the development and function of the gonadotropin hormone-releasing hormone system. Front. Endocrinol. (Lausanne) *4*, 133.

34. Liu, F., Placzek, M., and Xu, H. (2013). Axon guidance effect of classical morphogens Shh and BMP7 in the hypothalamo-pituitary system. Neurosci. Lett. *553*, 104–109.

35. Follett, B.K. (2014). "Seasonal changes in the neuroendocrine system": some reflections. Front. Neuroendocrinol. *37*, 3–12.

36. Yamamura, T., Hirunagi, K., Ebihara, S., and Yoshimura, T. (2004). Seasonal morphological changes in the neuro-glial interaction between gonadotropin-releasing hormone nerve terminals and glial endfeet in Japanese quail. Endocrinology *145*, 4264–4267.

37. Morgan, P.J., Webster, C.A., Mercer, J.G., Ross, A.W., Hazlerigg, D.G., MacLean, A., and Barrett, P. (1996). The ovine pars tuberalis secretes a factor(s) that regulates gene expression in both lactotropic and nonlactotropic pituitary cells. Endocrinology *137*, 4018–4026.

38. Stirland, J.A., Johnston, J.D., Cagampang, F.R., Morgan, P.J., Castro, M.G., White, M.R., Davis, J.R., and Loudon, A.S. (2001). Photoperiodic regulation of prolactin gene expression in the Syrian hamster by a pars tuberalis-derived factor. J. Neuroendocrinol. *13*, 147–157.

39. Sáenz de Miera, C., Hanon, E.A., Dardente, H., Birnie, M., Simonneaux, V., Lincoln, G.A., and Hazlerigg, D.G (2013). Circannual variation in thyroid hormone deiodinases in a short-day breeder. J. Neuroendocrinol. *25*, 412–421.

40. Herwig, A., de Vries, E.M., Bolborea, M., Wilson, D., Mercer, J.G., Ebling, F.J.P., Morgan, P.J., and Barrett, P. (2013). Hypothalamic ventricular ependymal thyroid hormone deiodinases are an important element of circannual timing in the Siberian hamster (Phodopus sungorus). PLoS ONE *8*, e62003.

41. Carr, A.J.F., Johnston, J.D., Semikhodskii, A.G., Nolan, T., Cagampang, F.R.A., Stirland, J.A., and Loudon, A.S.I. (2003). Photoperiod differentially regulates circadian oscillators in central and peripheral tissues of the Syrian hamster. Curr. Biol. *13*, 1543–1548.

42. Kim, T., Tao-Cheng, J.-H., Eiden, L.E., and Loh, Y.P. (2001). Chromogranin A, an "on/off" switch controlling dense-core secretory granule biogenesis. Cell *106*, 499–509.

43. Proudman, J.A., Clerens, S., van den Bergh, G., Garrett, W.M., Verhaert, P.D., Vandesande, F., and Berghman, L.R. (2003). Immunohistochemical localization of chromogranin A in gonadotrophs and somatotrophs of the turkey and chicken pituitary. Gen. Comp. Endocrinol. *132*, 293–303.

44. Ebenhöh, O., and Hazlerigg, D. (2013). Modelling a molecular calendar: the seasonal photoperiodic response in mammals. Chaos Solitons Fractals *50*, 39–47.

45. Song, J., Irwin, J., and Dean, C. (2013). Remembering the prolonged cold of winter. Curr. Biol. *23*, R807–R811.

46. Harper, C.V., Finkenstädt, B., Woodcock, D.J., Friedrichsen, S., Semprini, S., Ashall, L., Spiller, D.G., Mullins, J.J., Rand, D.A., Davis, J.R.E., and White, M.R. (2011). Dynamic analysis of stochastic transcription cycles. PLoS Biol. *9*, e1000607.

47. Hazlerigg, D.G., and Lincoln, G.A. (2011). Hypothesis: cyclical histogenesis is the basis of circannual timing. J. Biol. Rhythms *26*, 471–485.

48. Hazlerigg, D.G., Wyse, C.A., Dardente, H., Hanon, E.A., and Lincoln, G.A. (2013). Photoperiodic variation in CD45-positive cells and cell proliferation in the mediobasal hypothalamus of the Soay sheep. Chronobiol. Int. *30*, 548–558.

49. Migaud, M., Batailler, M., Pillon, D., Franceschini, I., and Malpaux, B. (2011). Seasonal changes in cell proliferation in the adult sheep brain and pars tuberalis. J. Biol. Rhythms *26*, 486–496.

50. Fredrich, M., Christ, E., Derouiche, A., and Korf, H.-W. (2015). Impact of melatonin on zeitgeber time-dependent changes in cell proliferation and apoptosis in the adult murine hypothalamic-hypophyseal system. Neuroendocrinology. Published online May 29, 2015. http://dx.doi.org/10.1159/000433440.

51. Montero-Hadjadje, M., Elias, S., Chevalier, L., Benard, M., Tanguy, Y., Turquier, V., Galas, L., Yon, L., Malagon, M.M., Driouich, A., et al. (2009). Chromogranin A promotes peptide hormone sorting to mobile granules in constitutively and regulated secreting cells: role of conserved N- and C-terminal peptides. J. Biol. Chem. *284*, 12420–12431.

52. Merks, T., Schulze-Bonhage, A., and Wittkowski, W. (1993). Photoperiod-dependent changes in exocytotic activity in the hypophyseal pars tuberalis of the Djungarian hamster, Phodopus sungorus. Cell Tissue Res. *273*, 287–291.

53. Wittkowski, W., Hewing, M., Hoffmann, K., Bergmann, M., and Fechner, J. (1984). Influence of photoperiod on the ultrastructure of the hypophysial pars tuberalis of the Djungarian hamster, Phodopus sungorus. Cell Tissue Res. *238*, 213–216.

54. Rütten, A., Hewing, M., and Wittkowski, W. (1988). Seasonal ultrastructural changes of the hypophyseal pars tuberalis in the hedgehog (Erinaceus europaeus L.). Acta Anat. (Basel) *133*, 217–223.

55. Nolan, L.A., and Levy, A. (2009). Prolonged oestrogen treatment does not correlate with a sustained increase in anterior pituitary mitotic index in ovariectomized Wistar rats. J. Endocrinol. *200*, 301–309.

56. Xu, P.X., Cheng, J., Epstein, J.A., and Maas, R.L. (1997). Mouse Eya genes are expressed during limb tendon development and encode a transcriptional activation function. Proc. Natl. Acad. Sci. USA *94*, 11974–11979.

57. Kozmik, Z., Holland, N.D., Kreslova, J., Oliveri, D., Schubert, M., Jonasova, K., Holland, L.Z., Pestarino, M., Benes, V., and Candiani, S. (2007). Pax-Six-Eya-Dach network during amphioxus development: conservation in vitro but context specificity in vivo. Dev. Biol. *306*, 143–159.

58. Salzer, C.L., Elias, Y., and Kumar, J.P. (2010). The retinal determination gene eyes absent is regulated by the EGF receptor pathway throughout development in Drosophila. Genetics *184*, 185–197.

59. Parkash, J., Messina, A., Langlet, F., Cimino, I., Loyens, A., Mazur, D., Gallet, S., Balland, E., Malone, S.A., Pralong, F., et al. (2015). Semaphorin7A

regulates neuroglial plasticity in the adult hypothalamic median eminence. Nat. Commun. *6*, 6385.

60. Prevot, V., Bellefontaine, N., Baroncini, M., Sharif, A., Hanchate, N.K., Parkash, J., Campagne, C., and de Seranno, S. (2010). Gonadotrophin-releasing hormone nerve terminals, tanycytes and neurohaemal junction remodelling in the adult median eminence: functional consequences for reproduction and dynamic role of vascular endothelial cells. J. Neuroendocrinol. *22*, 639–649.

61. Kameda, Y., Arai, Y., and Nishimaki, T. (2003). Ultrastructural localization of vimentin immunoreactivity and gene expression in tanycytes and their alterations in hamsters kept under different photoperiods. Cell Tissue Res. *314*, 251–262.

62. Yoshimura, T. (2013). Thyroid hormone and seasonal regulation of reproduction. Front. Neuroendocrinol. *34*, 157–166.

63. Nakao, N., Ono, H., Yamamura, T., Anraku, T., Takagi, T., Higashi, K., Yasuo, S., Katou, Y., Kageyama, S., Uno, Y., et al. (2008). Thyrotrophin in the pars tuberalis triggers photoperiodic response. Nature *452*, 317–322.

64. McNeilly, A.S., and Andrews, P. (1974). Purification and characterization of caprine prolactin. J. Endocrinol. *60*, 359–367.

Current Biology

Supplemental Information

Binary Switching of Calendar Cells in the Pituitary Defines the Phase of the Circannual Cycle in Mammals

Shona H. Wood, Helen C. Christian, Katarzyna Miedzinska, Ben R.C. Saer, Mark Johnson, Bob Paton, Le Yu, Judith McNeilly, Julian R.E. Davis, Alan S. McNeilly, David W. Burt, and Andrew S.I. Loudon

Figure S1: Photoperiodic treatments and the gross morphology

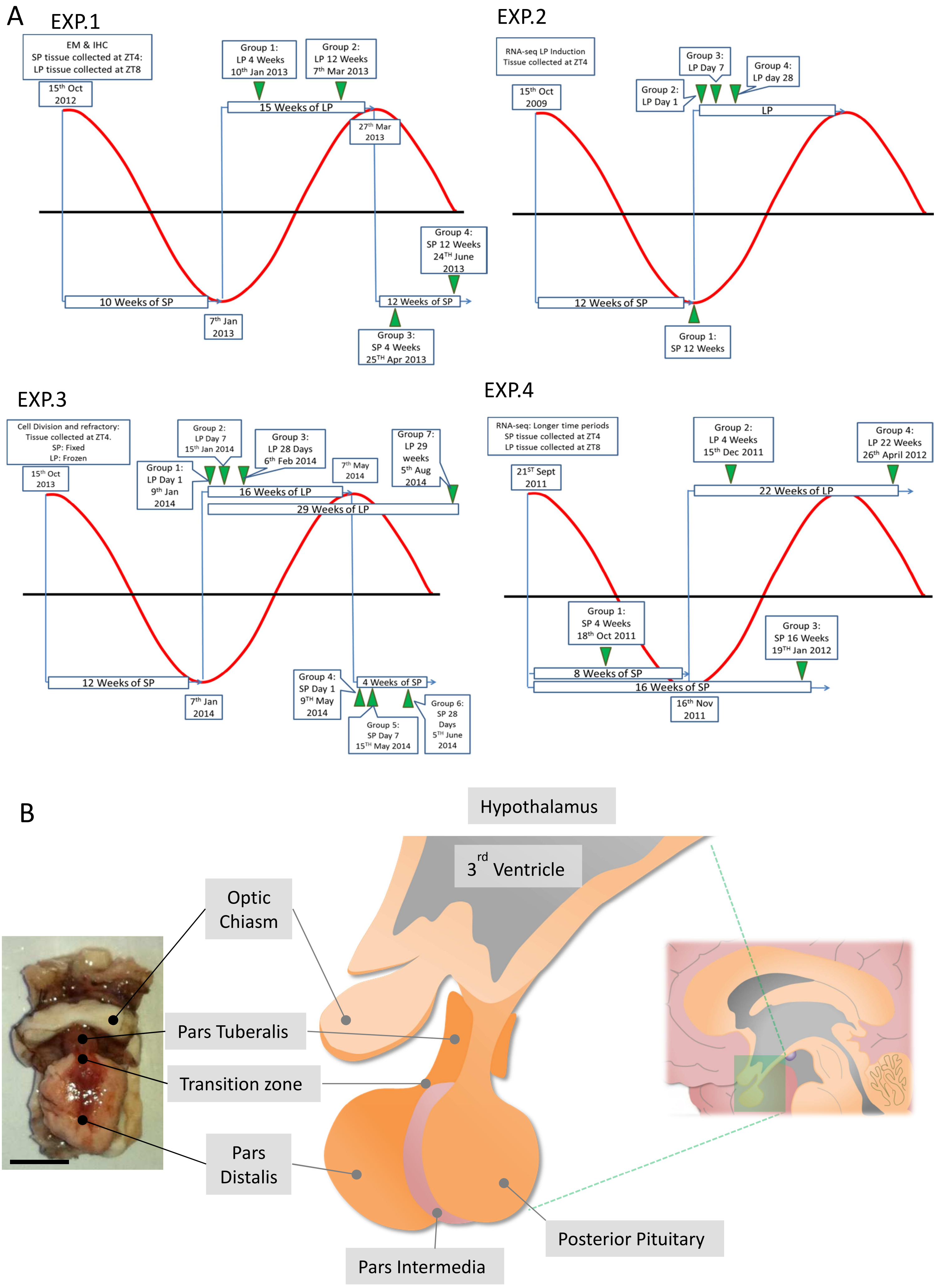

Figure S2: Characterisation of the cell types in the ovine pars tuberalis (PT) and pars distalis (PD) in a long and short photoperiod

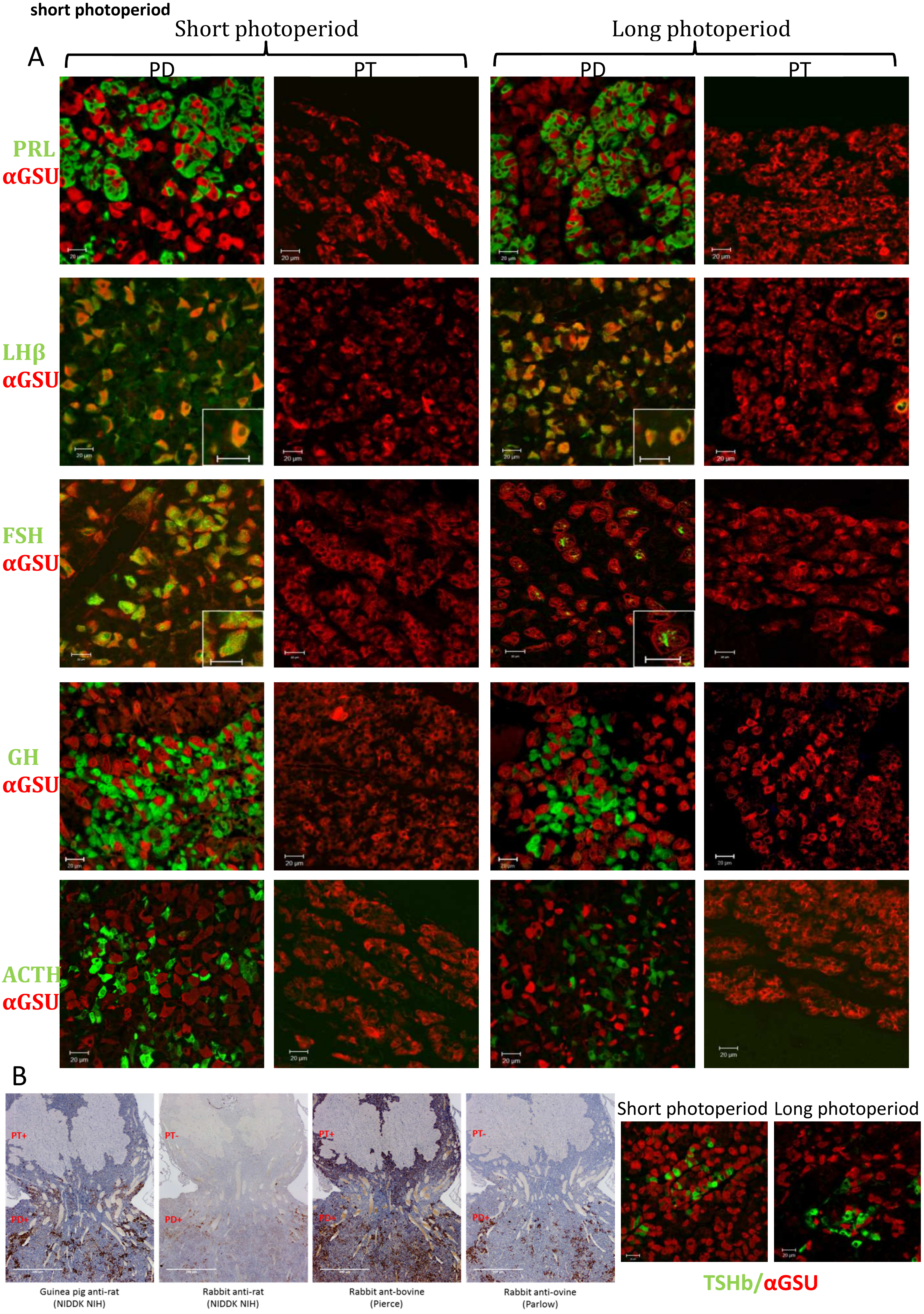

Figure S3: Major cell types of the PT and protein characterization

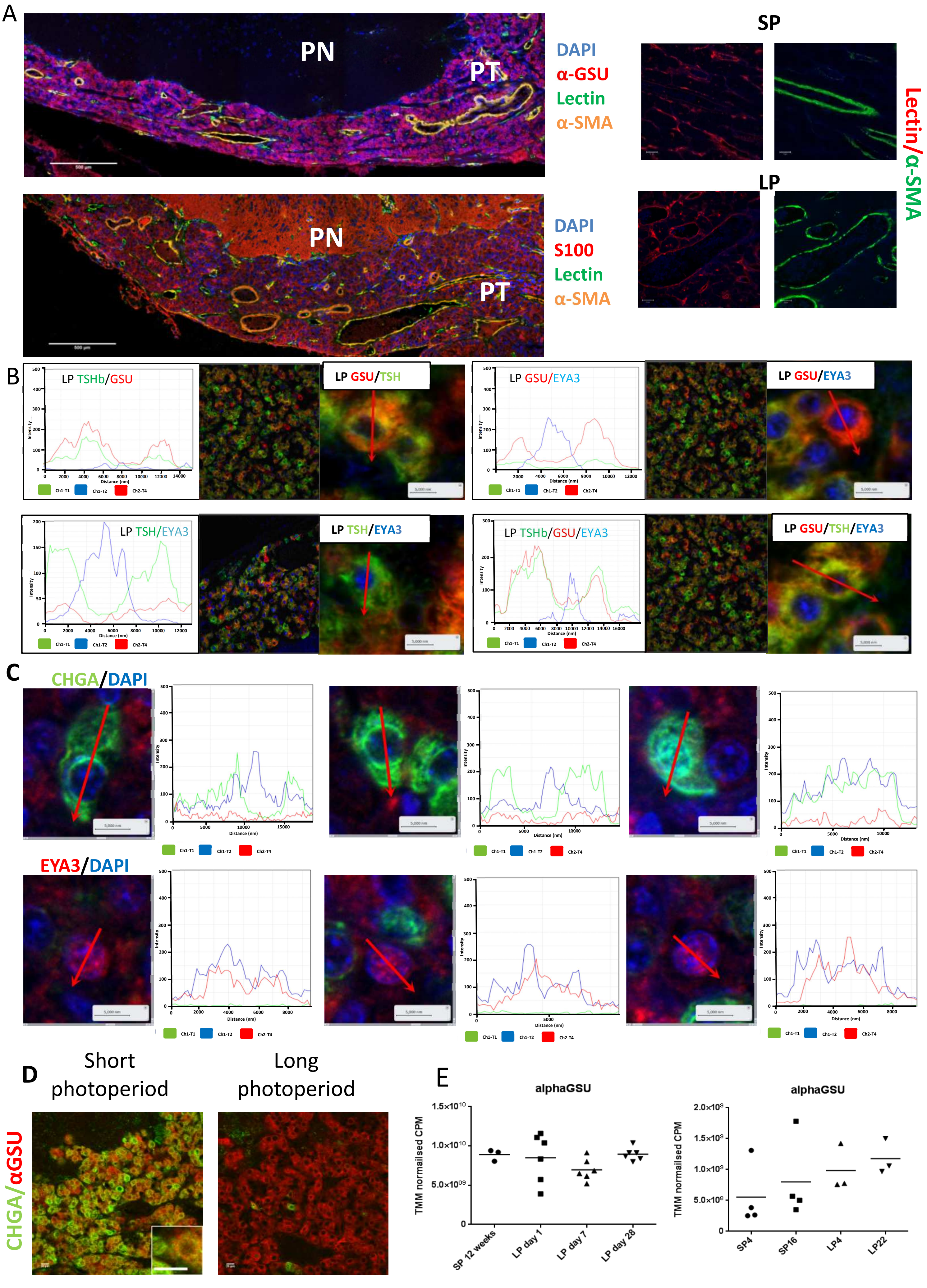

Figure S4: CHGA and EYA3 protein expression with individual prolactin concentrations, transcriptional characterisation and validation of SHH protein expression

A

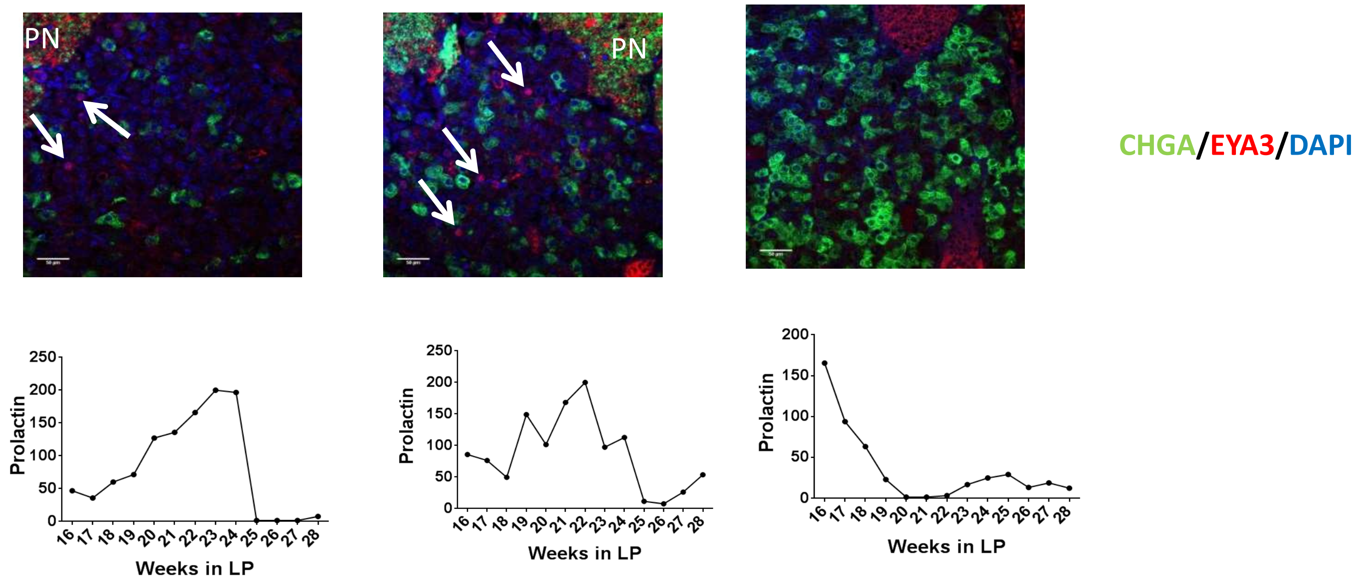

B

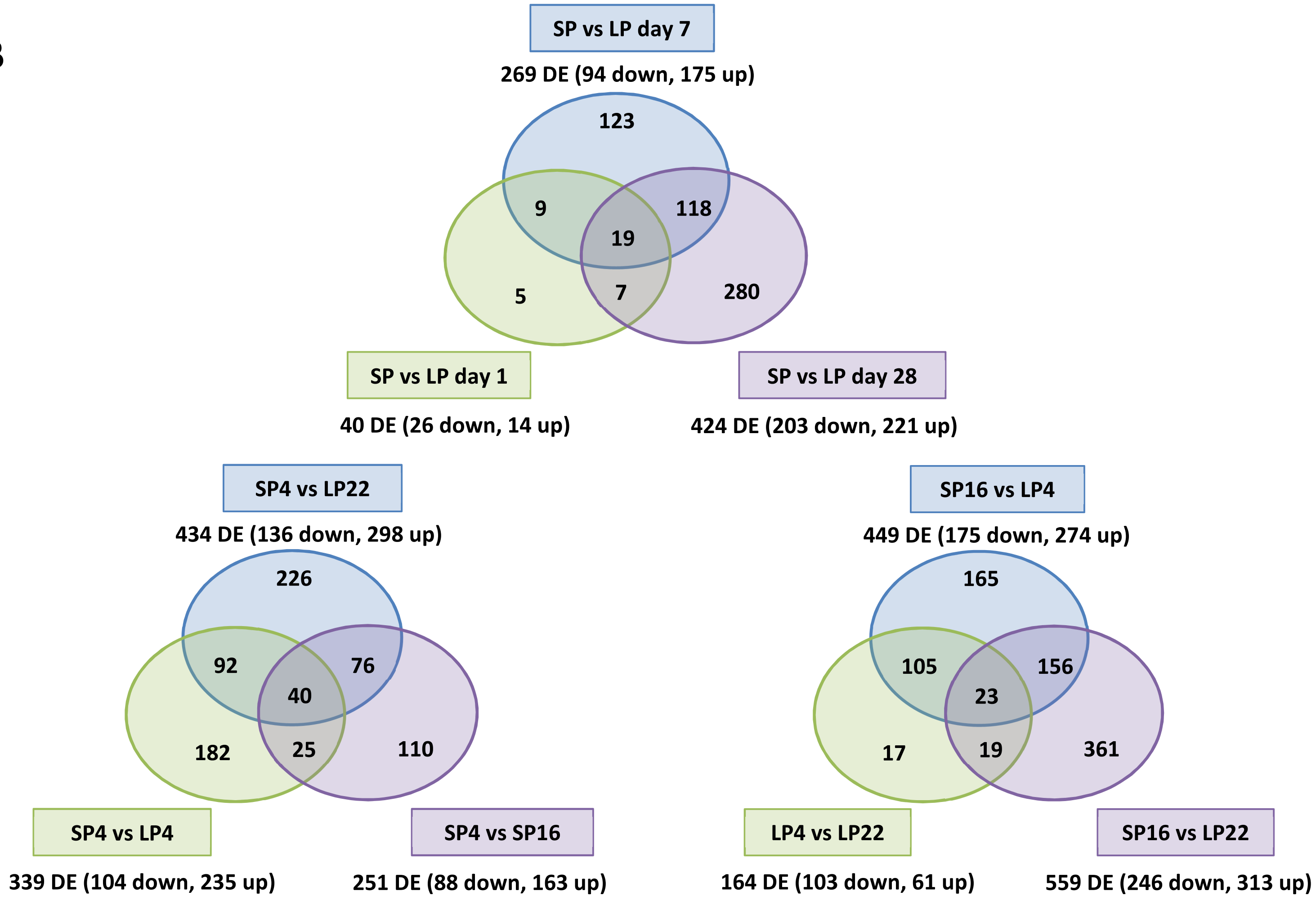

C

SHH(Green) and aGSU in PT long photoperiod

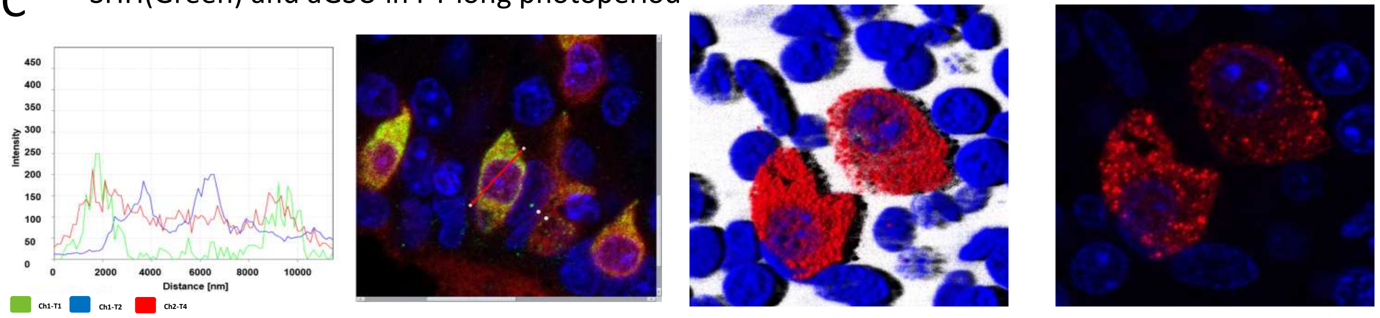

Figure S5: qPCR validation of RNA-seq, cell division marker Ki67 and CHGA co-localisation to granules

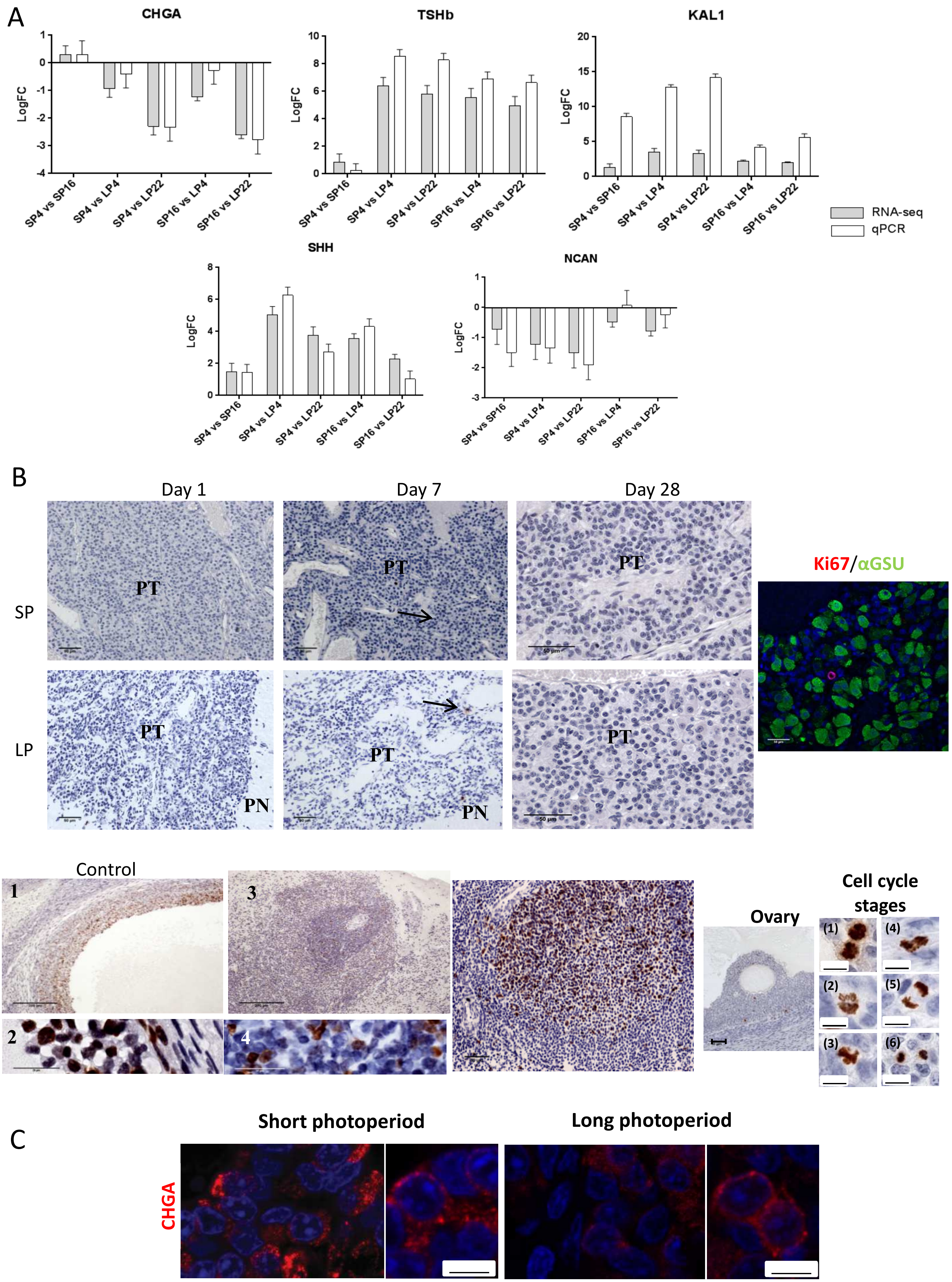

Figure S6: Cell cycle genes from RNA-seq

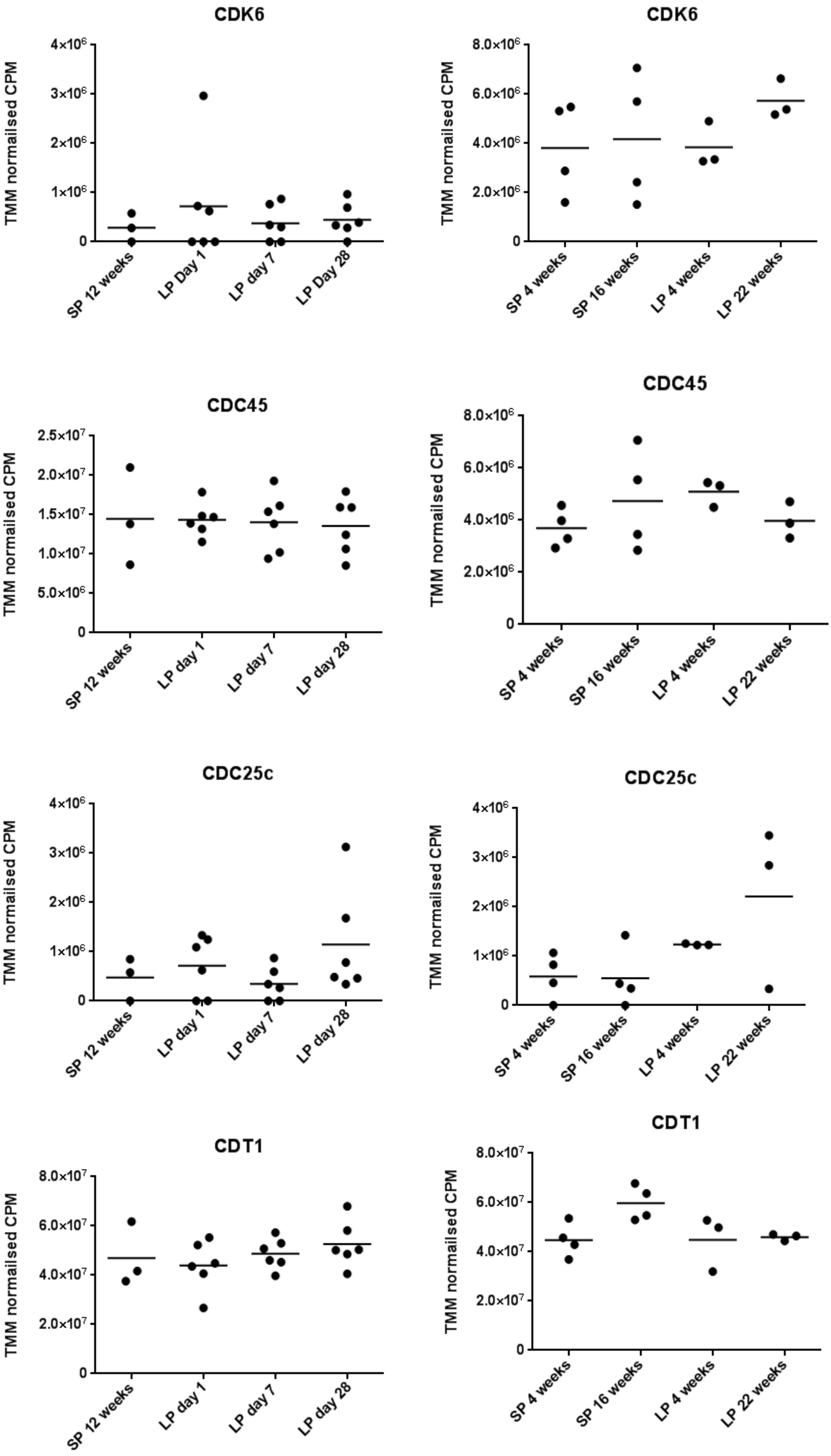

Figure legends

Figure S1: Photoperiodic treatments and the gross morphology relating to Figures 1 to 5

(A). Diagram of the photoperiodic treatments for Exp. 1 to 4. Collection points are represented by green arrows. The red line illustrates the natural photoperiod and the blue lines represent the photoperiod imposed in light controlled rooms.

(B). Diagram of the hypothalamus, optic chiasm, pars tuberalis, pars distalis, pars intermedia and posterior pituitary. Image of a hypothalamic block dissected from a sheep, indicating the transition zone between the PT and PD. Gonadotrophs (LHβ protein) have been previously reported in the PT [S1–S3], however expression was localized to this transition zone.

Figure S2: Characterisation of the cell types in the ovine pars tuberalis (PT) and pars distalis (PD) in a long and short photoperiod relating to Figure 1 & 2

(A). Double immunofluorescence showing an expression of αGSU (Red) with the main pituitary cell types (Green): lactotrophs (PRL), gonadotrophs (LH, FSH), somatotropes (GH), and corticotropes (ACTH). Scale bar=20µm.

(B). Four TSHb antibodies tested, Guinea pig anti-rat (NIDDK NIH) showing staining in the PT and PD, Rabbit anti-rat (NIDDK NIH) showing staining in the PD only, Rabbit anti-bovine (Pierce) showing staining in the PD and PT and Rabbit anti-ovine (Parlow) showing staining in the PD only. This is compatible with the recent discovery of tissue-specific glycosylation of PT-TSH, but not PD-TSH, as essential to maintaining their distinct functions [S4]; therefore it is likely that the post-translational modification of TSHβ [S5] affects the efficacy of the antibodies used. Double immunofluorescence showing an expression of αGSU (Red) with TSHb (Green) within the PD. Scale bar=20µm.

Figure S3: Major cell types of the PT and protein characterisation relating to Figures 1 & 2

(A). Triple immunohistochemistry of the PT and PN, sagittal section. αGSU staining for thyrotrophs (red) or S100 stain for folliculo-stellate cells (red), Lectin stain for blood vessels (green), alphaSMA stain for arterioles (orange). Venuoles are identified as lectin positive, alpha SMA negative Scale bar = 500um. PN – pars nervosa, PT – pars tuberalis. Smaller images for Lectin (red) and alpha-SMA (green) in SP and LP.

(B). Colour profiles and representative images used for the cell counting of TSHb (green), alphaGSU (red), and EYA3 (blue) in long photoperiod. Red arrows show the direction of scanning.

(C). Colour profiles and representative images of CHGA and EYA3, used for the cell counting, showing they are not co-localised. Red arrows show the direction of scanning.

(D). Double immunoflorescence showing expression of αGSU (Red) with CHGA (green) SP and LP in the PT or EYA3 (Red) with CHGA (green) SP and LP in the PT. (Table S3). Scale bar=20µm.

(E). TMM normalised counts from RNA-seq (Exp. 2 & 4) showing that αGSU expression, not significantly altered by photoperiod.

Figure S4: CHGA and EYA3 protein expression with individual prolactin concentrations, transcriptional characterisation and validation of SHH protein expression relating to Figures 2 & 3

(A). Protein expression of EYA3 (red) and CHGA (green) at LP29. Representative images showing that EYA3 and CHGA are not expressed in the same cell are included (5 animals, 6215 cells counted, Table S4). White arrows show EYA3 expressing cells. Scale bars = 50µm. Individual prolactin is also displayed for these animals, indicating a relationship between length of time that an animal exhibited sustained periods of low prolactin concentrations to the extent to which EYA3 and TSHβ protein declined and CHGA increased.

(B). Venn diagrams showing the number of significantly differentially expressed genes and the overlap with other comparisons. SP vs day 1, 7 and 28 are shown in the first venn diagram (Exp. 2). SP4 vs LP22, SP4 vs LP4 and SP4 vs SP16 are shown in the second venn diagram (Exp. 4) and finally SP16 vs LP4, LP4 vs LP22 and SP16 vs LP22 are shown in the last venn diagram (Exp. 4) (Tables S2)

(C). SHH (red) IHC showing co-localised to αGSU (green) expressing cells on long photoperiod. A 100% of SHH cells co-localize with αGSU.

Figure S5: qPCR validation of RNA-seq, cell division marker Ki67 and CHGA co-localisation to granules relating to Figures 3 & 4

(A). qPCR validation of RNA-seq results for TSHb, CHGA, KAL1, SHH, NCAN. RNA-seq LogFC shown in grey and qPCR LogFC shown in white. Error bars represent the standard error calculated as follows: (std error/mean)*log2e

(B). Detection of dividing cells by Ki67 and haematoxylin staining in the sheep pars tuberalis (PT) and (pars nervosa) under short and long photoperiod demonstrating only a minimal number (<0.2%) of single dispersed (arrows) dividing cells at day 1, 7 and 28 into the described photoperiod. Scale bar = 50um. For positive controls, sections spleen (3 – Bouins fixative, 5 – frozen tissue) were used and clearly show mitosis. Scale bar = 200um and 50um. Small images show positive staining of Ki67 in ovary (1 & 2) and spleen (3 & 4). Scale bar = 20um. Image 6 with a double immunofluorescent staining of aGSU (green) and Ki67 (red) in ovine PT shows that the dividing cells in the PT are not αGSU expressing cells. Sheep ovary for p-histone 3 clearly showing mitosis, Scale bar = 200µm. Small images (1-6) indicating p – histone H3 positive cells in ovary in different phase of the cell cycle, Scale bar = 10µm.

(C). IHC showing that CHGA (red) is localized to the granules in LP and SP. Scale bar = 5µm.

Figure S6: Cell cycle genes from RNA-seq relating to Figure 3

TMM normalised counts from the RNA-seq (Exp. 2 & 4) of four cell cycle related genes (CDK6, CDC45, CDC25c and CDT1), across groups SP, LP day 1, day 7 and day 28 (Exp. 2) and LP4, SP4, SP16 and LP22 (Exp. 4). No significant differences identified by EdgeR.

Table S1: Cell counts for TSHβ, EYA3 and αGSU in long photoperiod

N=3 animals, 2 sections from each animal, 2 fields from each section

Sheep no. LP3 - 75						
		Field 1	Field 2	Field 3	Field 4	Total n=4
	TSH	200	159	97	202	658
	GSU	173	193	139	192	697
	TSH + GSU	97	108	72	142	419
	Eya3	204	199	119	145	642
	TSH + Eya3	141	106	75	103	425
	GSU + Eya3	117	147	77	114	455
	GSU + TSH + Eya3	105	91	49	85	330
Sheep no. LP3- 76						
		Field 1	Field 2	Field 3	Field 4	Total n=4
	TSH	192	185	136	144	627
	GSU	236	221	255	186	898
	TSH + GSU	136	136	156	108	536
	Eya3	162	123	182	149	616
	TSH + Eya3	109	47	50	92	298
	GSU + Eya3	123	53	75	109	360
	GSU + TSH + Eya3	82	79	98	53	312
Sheep no. LP3 - 77						
		Field 1	Field 2	Field 3	Field 4	Total n=4
	TSH	175	222	161	271	829
	GSU	220	228	171	219	838
	TSH + GSU	143	159	121	241	667
	Eya3	207	233	163	153	756
	TSH + Eya3	136	147	115	93	491
	GSU + Eya3	151	153	119	113	536
	GSU + TSH + Eya3	119	123	95	92	429

Table S2: Statistically significant differentially expressed genes from the RNA-seq experiments

Exp. 2 comparisons: SP 12 weeks (ZT4) to LP day 1 (ZT4),) SP 12 weeks (ZT4) to LP day 7 (ZT4), SP 12 weeks (ZT4) to LP day 28 (ZT4).

Exp. 4 comparisons: SP 4 weeks (ZT4) to LP 4 weeks (ZT8), SP 4 weeks (ZT4) to SP 16 weeks (ZT4), SP 16 weeks (ZT4) to LP 4 weeks (ZT8), SP 4 weeks (ZT4) to LP 22 weeks (ZT8), LP 4 weeks (ZT8) to LP 22 weeks (ZT8), SP 16 weeks (ZT4) to LP 22 weeks (ZT8).

Supplied as an excel file

Table S3: Cell counts for CHGA and αGSU in long and short photoperiod

N=2 animals, 2 sections from each animal, 2 fields from each section

LP control 1	Area 1	Area 2	Area 3	Area 4	Totals
CHGA	8	21	4	4	37
aGSU	246	261	211	221	939
Co-loc.	8	21	4	4	37
LP control 2	Area 1	Area 2	Area 3	Area 4	Totals
CHGA	18	13	12	8	51
aGSU	233	219	131	133	716
co-loc.	18	13	12	8	51
SP control 1	Area 1	Area 2	Area 3	Area 4	Totals
CHGA	310	186	245	207	948
aGSU	324	185	286	238	1033
Co-loc.	310	186	245	207	948
SP control 2	Area 1	Area 2	Area 3	Area 4	Totals
CHGA	360	268	221	419	1268
aGSU	380	291	280	435	1386
Co-loc.	360	268	221	419	1268

Table S4: Cell counts for refractory animals: TSHβ, CHGA and EYA3

N = 3 animals, 4 sections from each animal, 2 fields of view per section

TSHb and EYA3 in refractory

	Area 1	Area 2	Area 3	Area 4	Area 5	Area 6	Area 7	Area 8	Total number
TSH 327	36	68	44	46	56	48	34	39	371
EYA327	0	0	0	0	0	0	0	0	0
Co-loc	0	0	0	0	0	0	0	0	0
	Area 1	Area 2	Area 3	Area 4	Area 5	Area 6	Area 7	Area 8	Total number
TSHb 519	108	91	81	27	89	101	114	134	745
EYA3 519	2	3	5	2	4	11	4	7	38
co-loc	2	3	5	2	4	11	4	7	1
	Area 1	Area 2	Area 3	Area 4	Area 5	Area 6	Area 7	Area 8	Total number
TSHb 520	57	92	112	98	142	121	84	80	786
EYA3 520	0	0	0	3	7	0	5	6	21
co-loc	0	0	0	3	5	0	3	4	15

CHGA & EYA3 in refractory and controls

	Area 1	Area 2	Area 3	Area 4	Area 5	Area 6	Area 7	Area 8	Total number
CHGA 327	210	179	213	176	199	197	205	180	1559
EYA3 327	0	0	0	0	0	0	0	0	0
CHGA 519	96	63	76	105	81	64	81	80	646
EYA3 519	0	0	0	7	5	11	5	6	34
CHGA 520	39	24	51	52	86	67	49	48	416
EYA3 520	1	0	1	0	4	8	4	3	21
LP control CHGA	27	42	23	26	17	53	23	17	228
LP control EYA3	77	43	64	63	81	81	81	90	580
SP control CHGA	405	375	323	288	316	349	338	337	2731
SP control EYA3	0	0	0	0	0	0	0	0	0

Table S5: Fully annotated heat map for all RNA-seq comparisons

Supplied as an excel file

Table S6: List of genes associated with the term cell proliferation in the RNA-seq data set

Gene symbol	entrez-gene name
BCHE	BCHE : butyrylcholinesterase
CCK	CCK : cholecystokinin
CGA	CGA : glycoprotein hormones, alpha polypeptide
CHRM4	CHRM4 : cholinergic receptor, muscarinic 4
DCT	DCT : dopachrome tautomerase
EGR1	EGR1 : early growth response 1
ESR1	ESR1 : estrogen receptor 1
EZH2	EZH2 : enhancer of zeste 2 polycomb repressive complex 2 subunit
FSHB	FSHB : follicle stimulating hormone, beta polypeptide
GLUL	GLUL : glutamate-ammonia ligase
GPLD1	GPLD1 : glycosylphosphatidylinositol specific phospholipase D1
EPCAM	EPCAM : epithelial cell adhesion molecule
MT3	MT3 : metallothionein 3
NGFR	NGFR : nerve growth factor receptor
NPY	NPY : neuropeptide Y
PGR	PGR : progesterone receptor
PRL	PRL : prolactin
SFRP4	SFRP4 : secreted frizzled-related protein 4
SHH	SHH : sonic hedgehog
TAC1	TAC1 : tachykinin, precursor 1
GPR56	GPR56 : G protein-coupled receptor 56
LRRC17	LRRC17 : leucine rich repeat containing 17
TCFL5	TCFL5 : transcription factor-like 5 (basic helix-loop-helix)
HPSE	HPSE : heparanase
TPX2	TPX2 : TPX2, microtubule-associated
HHLA2	HHLA2 : HERV-H LTR-associating 2
GMNC	GMNC : geminin coiled-coil domain containing
ACVR1C	ACVR1C : activin A receptor, type IC

Supplemental Experimental procedures

Animals

All animal experiments were undertaken in accordance with the Home Office Animals (Scientific Procedures) Act (1986), UK, under a Project License held by A.L. Animals were castrated as lambs on the farm as part of normal agricultural practice, and use of this castrate model allowed studies of seasonal changes in gene expression without the complication of altered background levels of sex steroids.

Scottish blackface male castrate sheep were housed in artificial light dark cycles, either 8:16 h light ⁄ dark cycle for short photoperiod (SP) or 16:8 h light ⁄ dark cycle for long photoperiod (LP). There were 4 different experiments conducted. Exp. 1 was conducted in 2012/2013; animals were housed from October on SP for 10 weeks and switched to LP for 15 weeks, collecting samples at 4 weeks LP and 12 weeks LP at ZT8. Animals were switched back on to SP for 12 weeks, collecting at SP 4 weeks and SP 12 weeks at ZT4. These samples were used for electron microscopy and IHC. Exp. 2 was undertaken in 2009, the animals were housed from October on SP for 12 weeks and then switched to LP. The day on which the photoperiodic switch was applied was designated "day 0". Animals were then culled at day 1, 7 and 28 of LP at ZT4, these samples were used in the LP induction RNA-seq experiment. Exp. 3 was conducted in 2013/2014; animals were housed from October on SP for 12 weeks and then switched to LP, the day on which the photoperiodic switch was applied was designated "day 0". Animals were then culled at day 1, 7 and 28 of LP at ZT4. The remaining animals were maintained on LP for 16 weeks, switching a cohort back to SP and collecting at day 1, 7 and 28, ZT4. These samples were used for the cell division experiments and IHC. Exp. 4 was conducted in 2011/2012; animals were housed from October on SP for 8 weeks, a cohort were maintained on SP up to 16 weeks and the other cohort were switched to LP for 22 weeks and used for the second RNA-seq experiment. Samples were collected in the mid-light phase at SP 4 weeks, SP 16 weeks, LP 4 weeks and LP22 weeks (ZT4 – SP, ZT8 – LP). A cohort of animals was maintained on LP for 29 weeks, prolactin was measured over the course of the experiment and the samples were used for EM and IHC (Exp. 3, ZT4). Finally, archived material was also used as previously described [S6, S7] for additional IHC studies. Figure S1 shows the design of all the experiments.

All animals were killed by an overdose of barbiturate (Euthatal; Rhone Merieux, Essex, UK) administered intravenously. Hypothalamic blocks with the pars tuberalis and pituitary attached were collected for immunohistochemistry, electron microscopy and transcriptomics (Figure S1B).

Prolactin assay and defining "refractory"anmals

Ovine prolactin (oPRL) was measured using a newly developed competitive ELISA using purified oPRL (ovine prolactin NIDDK-oPRL-21; AFP10692C; from Dr. A Parlow, NHPP, Harbor-UCLA Torrance CA, USA) and a highly specific rabbit anti-ovine prolactin (ASM-R50, produced by ASM) used previously in a specific radioimmunoassay [S8]. Three replicates of oPRL (100µg each) were biotinylated using NHS-LC-Biotin (Thermo Pierce; Fisher Scientific UK Ltd, Loughborough, UK). And the resulting biotinylated oPRL preparations were assessed in the ELISA. When test control samples were assayed, the ELISA gave results, which were comparable to the levels previously measured by the original RIA. The ELISA was established on standard 96 well plates (Nunc Maxisorb Immuno Plates; Nunc A/S, Roskilde, Denmark) previously coated with capture antibody (affinity purified donkey anti-rabbit IgG; Jackson ImmunoResearch Laboratories Inc, Newmarket, Suffolk CB8 7SY UK). The rabbit anti-oPRL antiserum (ASM-R50; 1:160,000 dilution) was added followed by addition of samples or standards and biotinylated oPRL, and plates incubated for at least 14h at 4°C. Plates were then washed 5 times before detection of biotinylated oPRL by addition of streptavidin HRP (GE Healthcare UK, Little Chalfont, UK) for a minimum of 30 minutes followed by TMB peroxidase substrate (KPL, Gaithesburg, MD 20878, USA). Colour was allowed to develop for up to 10 min, then stopped by addition of 6% phosphoric acid, and plates were read at 450nm. The Coefficient of Variation for the assay on control plasma samples was <10%.

We calculated the mean prolactin concentration in SP across multiple weeks and animals (n=32) and then calculated the median of the means and added 20% to define a SP-like prolactin concentration (SP prolactin = 41.03). We then defined an animal as "refractory" when their individual prolactin level remained at SP-like concentrations for 3 consecutive weeks. Once an animal met this criterion we noted the first week the animal had a SP-like prolactin value and defined the animal as refractory from that week.

EYA3 antibody

A polyclonal EYA3 antibody was raised in chicken (Cambridge research biochemical, Billingham, UK). The peptide sequence was based on homology with the sheep and human (Acetyl-PEQPVKKAKMQESGEQTLS-[C]-amide). The ovine EYA3 peptide was prepared at >80% purity and conjugated with KLH for immunization. Protein precipitation and isolation of IgY from eggs was performed and ELISA analysis at day 0, 49, 63, 77, 91 and 105 were carried out. Two elutions per antibody were supplied: Glycine and TEA. The glycine eluate was used for IHC.

Immunohistochemistry

Tissues were immersed in Bouin's fixative for 8 hours, transferred to 70% ethanol, then dehydrated and embedded in paraffin wax. Sections (5 µM) were cut, floated onto Superfrost Plus slides (J1800 AMNZ, Thermo scientific), dried at 50°C overnight, then dewaxed and rehydrated.

Frozen sections (8 µM) were collected on to Poly-lysine coated slides and stored in -80°C. Before immuno-detection, sections were fixed for 10 min at room temperature in 4% paraformaldehyde then rinsed in PBS. For localization of dividing cell markers standard single immunofluorescence was performed.

Double immunofluorescence was carried out for co-localization of PRL, LHβ, FSHβ, GH, TSHβ, ACTH, S100 and CHGA antigens with α-GSU. Slides were washed in PBS buffer (P4417, Sigma) between treatments. For LH and CHGA, antigen retrieval [S9] was carried out by pressure cooking for 5 min at full pressure in 0.01 M citrate buffer, pH 6.0. For all sections endogenous peroxidase activity was blocked by incubating sections in 3% (v/v) hydrogen peroxidase in methanol for 30 min. Sections were blocked using 20% normal goat serum, 5% BSA in PBS (NGS/PBS/5%BSA) for 30 min, then incubated in a humidified chamber overnight at 4°C in the first primary antibody diluted in blocking buffer as indicated. Antibody dilutions were as follows: rabbit anti – PRL 1:100K (R51, ASM-HRSU), mouse anti-LHβ 1:50K (kindly gifted by J. Roser, University of California), rabbit anti-FSHβ 1:25K (kindly gifted by S. Lynch, Birmingham), mouse anti-GH 1:500 (gift from Prof. M. Wallis, University of Sussex, Brighton), rabbit anti-bovine TSH 1:2K (kindly gifted by J. Pierce), mouse anti-ACTH 1:100 (NCL-ACTH, Novocastra), rabbit anti-S100α (ab11428, Abcam) 1:500, rabbit anti-CHGA 1:4K (Incstar), and αGSU 1:2K (ASM-HRSU, R20). Slides where then incubated with first secondary antibodies. PRL, FSHβ, TSHβ, S100α, FSHβ and α-GSU slides were incubated with goat anti-rabbit peroxidase (Vector Laboratories PI-1000) diluted 1:500 for 1h. LH, GH, ACTH slides were incubated with goat anti – mouse peroxidase (Abcam, ab6823) diluted 1:500 for 1 h, then followed by TSA (NEL741B00IKT, Perkin Elmer) diluted 1:50 in kit buffer for 10 min in the dark. PRL, FSHβ, TSHβ, S100α and a-GSU slides were performed in 0.01 M citrate buffer (2.5 min in boiling solution in microwave followed by a cool down at room temperature for at least 15 min [S10]. After that all sections were blocked in NGS/PBS/5%BSA for 30 min, and then incubated overnight at 4°C in the second primary antibody, αGSU, diluted 1:2.5K in blocking buffer or in the case of slides labelled with first primary antibody αGSU were incubated with CHGA antibody. All section were then incubated in the second secondary antibody, either goat anti-rabbit or mouse peroxidise IgG diluted 1:500 (Vector Laboratories PI-1000) for 1 hours, followed by TSA (NEL744B00IKT, Perkin Elmer) diluted 1:50 in kit buffer for 10 min in the dark. Control sections were incubated with NGS/PBS/5%BSA in place of the first, second primary or both first and second primary antibodies. For localization of αGSU or S100α with αSMA (mouse anti-αSMA at 1:1K (M0851, Dako)) and Lectin standard double immunofluorescence with an additional stage for third primary and secondary antibodies was performed. For S100 and αSMA detection antigen retrieval was carried out by boiling in 0.01 citrate buffer pH6 for 6.5min in a microwave. First primary antibody dilutions were as follows: rabbit anti – human S100 1:500, rabbit anti – ovine αGSU 1:2K. Slides where then incubated with first secondary antibodies goat anti – rabbit peroxidase, and then followed by TSA (NEL745001KT, Perkin Elmer). The second primary antibody, mouse anti – human αSMA (M0851, Dako) was diluted 1:2K follow by the second secondary antibody, goat anti – mouse peroxidase (ab6823, Abcam) and then TSA (NEL741001KT). The third primary antibody biotin labelledlabelled Lectin, (L3759, Sigma) diluted 1:40 in the serum was detected using Streptavidin Alexa 488 (S11223, Molecular Probes) at 1:200 dilution in PBS. Subsequently, all samples were incubated in DAPI (D9542, Sigma) diluted 1:500 in PBS for 10 min in the dark. Sections were coverslip mounted using PermaFluor™ mounting medium (TA-030-FM, Thermo scientific). All samples were imaged under a Zeiss LSM 710/510 confocal microscope with a 40× oil-immersion objective. The cell counting was done manually using Adobe Photoshop CS6.

Conventional methods were used for the immunohistochemical detection of Ki67 and phosphor-histone H3, markers of dividing cells and PC1. lides were washed in TBS pH=7.4 buffer between treatments. The primary antibodies were diluted at 1:100 for Ki67 and 1:500 for phospho–histone H3 and were detected using biotinylated secondary goat

anti – rabbit in 1:500 dilution and streptavidin-peroxidase conjugate in 1:1000 dilution. Peroxidase activity was detected using a DAB solution. Sections were counterstained with Mayer's haematoxylin, then dehydrated and coverslip mounted using Pertex mounting medium.

In Situ Hybridization (ISH) and Quantification of Signal

The *OaTSHβ* plasmid (XM_004002368.2) was kindly provided by David Hazlerigg. The *OaEya3* plasmid (NM_001161733.1) was cloned as previously described [S6]. Frozen coronal ovine hypothalamic blocks for in-situ hybridization were cut into 16μm sections using a cryostat (CM3050s Leica Microsystems, Ltd., Milton Keynes, UK), and thaw mounted onto poly-l-lysine coated slides (VWR International, Lutterworth, UK). Radiolabelled cRNA riboprobes were prepared by plasmid linearization and transcribed using P^{33} α-UTP (Perkin-Elmer). Fixed sections were hybridized overnight at 60°C with 15 x 10^5 cpm of probe per slide. Hybridization signals were visualised on autoradiographic film (Kodak Biomax MR Films, Kodak, USA) after one week exposure at -80°C. Signal intensity was quantified by densitometry analysis of autoradiographs using the image-Pro Plus 6.0 software (Media Cybernetics, Inc., Marlow, UK) using 3 animals per group.

Tissue processing and electron microscopy (EM)

Hypothalamo-pituitary tissue blocks were fixed by immersion in 3% paraformaldehyde/0.05% paraformaldehyde in 0.1M phosphate buffer (pH 7.2) for 24 hours at room temperature and transferred to a 1:10 dilution of the fixative in 0.1M phosphate buffer for storage at 4°C before processing. Using a scalpel blade, areas from the medial PT and median eminence were cut into 0.5mm^3 pieces which were then stained with osmium (1% in 0.1M phosphate buffer), uranyl acetate (2% w/v in distilled water), dehydrated through increasing concentration of ethanol (70 to 100%), followed by 100% acetone and embedded in Spurr's resin (TAAB laboratory equipment, Aldermarston, UK). Ultrathin sections (50-80 nm) were prepared using a Reichart-Jung Ultracut ultramicrotome and mounted on nickel grids (Agar Scientific Ltd., Stanstead, UK). Sections were then counterstained with lead citrate and uranyl acetate and examined on a JOEL 1010 transmission electron microscope (JOEL USA Inc., Peabody, MA, USA). Sections from 3 animals per group were examined.

Quantitative electron microscopy morphological studies

For analysis of PT cell morphology, twenty micrographs per animal (n=3 sheep per group) of individual PT cells were taken at a magnification of x 5,000. Negatives were scanned into Adobe Photoshop CS2 (Adobe Corp., San Jose, CA, USA) and analysed using Axiovison version 4.5 (Zeiss, Oberkochen, Germany) image analysis software. The analyst was blind to the sample code. The following parameters were calculated: cytoplasmic, nuclear and total cell areas; granule area, granule areal density (granule area divided by cytoplasmic area x100 to express as %) and granule diameter. For measurement of the cell and nuclear areas, margins were drawn around the cell or nucleus respectively and the area was calculated. Cytoplasmic area was determined by subtracting nuclear area from total cell area. Granule number was counted and granule density was calculated by dividing total granule area by cytoplasmic area. Expansion of the rough endoplasmic reticulum (rough ER) and Golgi apparatus was assessed visually and graded on a scale of 0-4 (0, no expansion; 4 the most expansion). These estimates do not provide absolute measurements but do provide a basis for comparison.

Three measurements were made of the external zone of the median eminence using Axiovision from micrographs taken at x2500. 20 micrographs per sheep were analysed as follows. (1) The distance from the ends of nerve terminals to the basal lamina was measured. (2) In an area of 800μm^2 the area occupied by tanycytes was measured and expressed as a % of the total area. (3) The % of terminals that made contact with the basal lamina was counted in a 100μm region of external ME. Tanycytes were identified using criteria described by Ganten & Pfaff [S11]. All morphometric values represent the mean ± SEM (n=3 sheep per group). Means were compared by one way analysis of variance (ANOVA) with *post hoc* analysis by the Bonferroni test. P<0.05 was considered statistically different.

RNA-seq Assays

The pars tuberalis was dissected to minimise inclusion of transition zone and median eminence, though complete isolation of the PT is difficult to achieve. The PT samples were snap frozen on dry ice and stored at -80C. RNA was extracted from the pars tuberalis using Qiagen's TissueLyser II and RNeasy tissue kit. The quality of the extracted RNA was assessed using the Agilent 2100 Bioanalyser; all RNA integrity numbers (RINs) were above 8, indicating that good quality RNA had been extracted. Poly-A selection was used.

The library preparation protocol was carried out according to the manufacturer's instructions by Edinburgh Genomics. The LP induction experiment (Exp. 2) was sequenced using the Illumina genome analyzer II with 100 nucleotide single end (SE) reads, whereas the photoperiodic effect over longer time periods (Exp. 4) was sequenced on the Illumina Hiseq 2500 with 100 nucleotide paired end (PE) reads. The difference in the sequencing platforms means that we did not directly compare these experiments; we only compared the differentially expressed (DE) gene lists after all statistical processing was complete to avoid platform biases.

The FASTQ files were mapped to the Ensembl release 78 sheep reference genome (Oar_v3.1) using Bowtie [S12]. Approximately 60% of reads generated were uniquely mapped. All data have been submitted to GEO under the accession GSE65901.

Bioinformatics and Gene expression analysis

In order to measure gene expression from mapped RNA-seq data, the BAM files from Bowtie mapping were sorted using SAMtools [S13]. Raw counts per gene were estimated by the Python script HTSeq count (http://www-huber.embl.de/users/anders/HTSeq/) using the Ensembl sheep reference genome 78. The raw counts per gene were used by EdgeR [S14] to estimate differential expression (DE).

EdgeR (Bioconductor release 2.9) uses a pair-wise design to measure differential gene expression. The analysis is based on a negative binomial model that uses over-dispersion estimates to account for biological variability (i.e. sample to sample differences); this is an alternative to the Poisson estimates of biological variability that are often inappropriate [S15]. Counts per million (cpm) were calculated and only genes with 1 cpm in at least 3 samples was included in the analysis. Trimmed mean of M-values (TMM) normalisation of the sequenced libraries was performed to remove effects due to differences in library size [S14–S17]. EdgeR generates a fold change for each gene, p values and the Benjamin-Hochberg false discovery rate (FDR) are calculated to statistically test the measured DE. As in previous studies, no effect size cut-off was set [S18].

Enrichment analysis of GO terms

To assess the biological significance of gene expression changes across all the datasets we combined all the DE genes, removed DE non-coding RNAs, assigned GO terms to each of the genes and then sorted them by shared terms and log fold changes to create a heat-map.

To assess these datasets in a more stringent manner we used cytoscape plug-in ClueGO to perform an enrichment analysis on each individual comparison and then using a built-in algorithm the GO terms were collapsed based on related terms and statistical significance in order to give a simplified network [S19, S20]. Further enrichment analysis was performed by GSEA [S21, S22], consensusPathDB [S23] and DAVID [S24].

qPCR validation

To generate cDNA for qPCR, 2 µg of total RNA was reverse transcribed using the applied biosciences cDNA synthesis kit. The Roche universal probe library designer was used to design primers (https://www.roche-appliedscience.com/servlet/) with sequences obtained from Ensembl. All primers were designed to cross an exon–exon boundary. The specificity of the primers was checked using BLAST (http://www.ncbi.nlm.nih.gov/BLAST/). A reference gene experiment was conducted to identify the most stably expressed genes in the pars tuberalis (data not shown); HPRT1 and YWHAZ were the most stably expressed and were used to normalise the qPCR results. The qPCR

assays were all performed in duplicate using a TaqMan™ ABI PRISM 7500 fast (Applied Biosystems, Foster City, CA, USA) in 96-well plate format. A 20-ml reaction volume was used per well, consisting of: 10 µl Taqman 2× PCR master mix (Universal PCR Mastermix; Applied Biosystems),

0.2 µl each of 20 mM forward and reverse primers, 0.2 µl of 10 mM probe, 0.2 µl distilled water and 9.2 µl of cDNA or water for the negative controls. The amplification was performed as follows: 2 min at 50°C, 10 min at 95°C followed by 40 cycles of 95°C for 15 s and 60°C for 1 min. The efficiency of the assays were between 93 % and 107 % and the R2 values were >0.98. The ΔΔcT method was used to measure expression; "SP 4 weeks" was used as the reference samples from which relative expression was calculated. The data were further corrected by the efficiency of the standard curve for each gene. Log2 fold change relative to "SP 4 weeks" was calculated and compared to the RNA-seq results in order to confirm the expression results. The standard error was calculated for log2 fold change as follows: (std error/mean)*log2e. For qPCR the relative quantification values were used to calculate standard error. For RNA-seq, raw reads converted into relative values were used to calculate standard errors.

References

S1. Pelletier, J., Counis, R., de Reviers, M. M., Moumni, M., and Tillet, Y. (1995). Changes in LHbeta-gene and FSHbeta-gene expression in the ram pars tuberalis according to season and castration. Cell Tissue Res. *281*, 127–33.

S2. Pelletier, J., Counis, R., de Reviers, M. M., and Tillet, Y. (1992). Localization of luteinizing hormone beta-mRNA by in situ hybridization in the sheep pars tuberalis. Cell Tissue Res. *267*, 301–6.

S3. Gross, D. S. (1984). The mammalian hypophysial pars tuberalis: a comparative immunocytochemical study. Gen. Comp. Endocrinol. *56*, 283–98.

S4. Ikegami, K., Liao, X.-H., Hoshino, Y., Ono, H., Ota, W., Ito, Y., Nishiwaki-Ohkawa, T., Sato, C., Kitajima, K., Iigo, M., et al. (2014). Tissue-Specific Posttranslational Modification Allows Functional Targeting of Thyrotropin. Cell Rep. *9*, 801–809.

S5. Weintraub, B. D., Stannard, B. S., Magner, J. A., Ronin, C., Taylor, T., Joshi, L., Constant, R. B., Menezes-Ferreira, M. M., Petrick, P., and Gesundheit, N. (1985). Glycosylation and posttranslational processing of thyroid-stimulating hormone: clinical implications. Recent Prog. Horm. Res. *41*, 577–606.

S6. Dardente, H., Wyse, C. A., Birnie, M. J., Dupré, S. M., Loudon, A. S. I., Lincoln, G. A., and Hazlerigg, D. G. (2010). A molecular switch for photoperiod responsiveness in mammals. Curr. Biol. *20*, 2193–8.

S7. Dupré, S. M., Miedzinska, K., Duval, C. V, Yu, L., Goodman, R. L., Lincoln, G. A., Davis, J. R. E., McNeilly, A. S., Burt, D. D., and Loudon, A. S. I. (2010). Identification of Eya3 and TAC1 as long-day signals in the sheep pituitary. Curr. Biol. *20*, 829–35.

S8. McNeilly, A. S., and Andrews, P. (1974). Purification and characterization of caprine prolactin. J. Endocrinol. *60*, 359–67.

S9. Norton, A. J., Jordan, S., and Yeomans, P. (1994). Brief, high-temperature heat denaturation (pressure cooking): a simple and effective method of antigen retrieval for routinely processed tissues. J. Pathol. *173*, 371–9.

S10. Tóth, Z. E., and Mezey, E. (2007). Simultaneous visualization of multiple antigens with tyramide signal amplification using antibodies from the same species. J. Histochem. Cytochem. *55*, 545–54.

S11. Ganten, D., and Pfaff, D. (1986). Morphology of hypothalamus and its connections. In Current topics in Neuroendocrinology (Berlin: Springer Verlag).

S12. Langmead, B., Trapnell, C., Pop, M., and Salzberg, S. L. (2009). Ultrafast and memory-efficient alignment of short DNA sequences to the human genome. Genome Biol. *10*, R25.

S13. Li, H., Handsaker, B., Wysoker, A., Fennell, T., Ruan, J., Homer, N., Marth, G., Abecasis, G., Durbin, R., and Data, G. P. (2009). The Sequence Alignment/Map format and SAMtools. Bioinformatics *25*, 2078–9.

S14. Robinson, M., Chen, Y., Mccarthy, D., and Smyth, G. K. (2010). edgeR : differential expression analysis of digital gene expression data How to get help Reading data General comments. Most.

S15. Oshlack, A., Robinson, M. D., and Young, M. D. (2010). From RNA-seq reads to differential expression results. Genome Biol. *11*, 220.

S16. Robinson, M. D., and Oshlack, A. (2010). A scaling normalization method for differential expression analysis of RNA-seq data. Genome Biol. *11*, R25.

S17. Robinson, M., Mccarthy, D., Chen, Y., and Smyth, G. K. (2011). edgeR : differential expression analysis of digital gene expression data User ’ s Guide.

S18. Wood, S. H., Craig, T., Li, Y., Merry, B., and de Magalhães, J. P. (2013). Whole transcriptome sequencing of the aging rat brain reveals dynamic RNA changes in the dark matter of the genome. Age (Dordr). *35*, 763–76.

S19. Smoot, M. E., Ono, K., Ruscheinski, J., Wang, P.-L., and Ideker, T. (2011). Cytoscape 2.8: new features for data integration and network visualization. Bioinformatics *27*, 431–2.

S20. Bindea, G., Mlecnik, B., Hackl, H., Charoentong, P., Tosolini, M., Kirilovsky, A., Fridman, W.-H., Pagès, F., Trajanoski, Z., and Galon, J. (2009). ClueGO: a Cytoscape plug-in to decipher functionally grouped gene ontology and pathway annotation networks. Bioinformatics *25*, 1091–3.

S21. Subramanian, A., Tamayo, P., Mootha, V. K., Mukherjee, S., Ebert, B. L., Gillette, M. A., Paulovich, A., Pomeroy, S. L., Golub, T. R., Lander, E. S., et al. (2005). Gene set enrichment analysis: a knowledge-based approach for interpreting genome-wide expression profiles. Proc. Natl. Acad. Sci. U. S. A. *102*, 15545–50.

S22. Mootha, V. K., Lindgren, C. M., Eriksson, K.-F., Subramanian, A., Sihag, S., Lehar, J., Puigserver, P., Carlsson, E., Ridderstråle, M., Laurila, E., et al. (2003). PGC-1alpha-responsive genes involved in oxidative phosphorylation are coordinately downregulated in human diabetes. Nat. Genet. *34*, 267–73.

S23. Kamburov, A., Stelzl, U., Lehrach, H., and Herwig, R. (2013). The ConsensusPathDB interaction database: 2013 update. Nucleic Acids Res. *41*, D793–800.

S24. Huang, D. W., Sherman, B. T., and Lempicki, R. a (2009). Systematic and integrative analysis of large gene lists using DAVID bioinformatics resources. Nat. Protoc. *4*, 44–57.
